# When the Most Potent Combination of Antibiotics Selects for the Greatest Bacterial Load: The Smile-Frown Transition

**DOI:** 10.1371/journal.pbio.1001540

**Published:** 2013-04-23

**Authors:** Rafael Pena-Miller, David Laehnemann, Gunther Jansen, Ayari Fuentes-Hernandez, Philip Rosenstiel, Hinrich Schulenburg, Robert Beardmore

**Affiliations:** 1 Biosciences, Geoffrey Pope Building, University of Exeter, United Kingdom; 2 Evolutionary Ecology and Genetics, Zoological Institute, CAU Kiel, Kiel, Germany; 3 Institute for Clinical Molecular Biology, CAU Kiel, Kiel, Germany; The Pennsylvania State University, United States of America

## Abstract

Conventional wisdom holds that the best way to treat infection with antibiotics is to ‘hit early and hit hard’. A favoured strategy is to deploy two antibiotics that produce a stronger effect in combination than if either drug were used alone. But are such synergistic combinations necessarily optimal? We combine mathematical modelling, evolution experiments, whole genome sequencing and genetic manipulation of a resistance mechanism to demonstrate that deploying synergistic antibiotics can, in practice, be the worst strategy if bacterial clearance is not achieved after the first treatment phase. As treatment proceeds, it is only to be expected that the strength of antibiotic synergy will diminish as the frequency of drug-resistant bacteria increases. Indeed, antibiotic efficacy decays exponentially in our five-day evolution experiments. However, as the theory of competitive release predicts, drug-resistant bacteria replicate fastest when their drug-susceptible competitors are eliminated by overly-aggressive treatment. Here, synergy exerts such strong selection for resistance that an antagonism consistently emerges by day 1 and the initially most aggressive treatment produces the greatest bacterial load, *a fortiori* greater than if just one drug were given. Whole genome sequencing reveals that such rapid evolution is the result of the amplification of a genomic region containing four drug-resistance mechanisms, including the *acrAB* efflux operon. When this operon is deleted in genetically manipulated mutants and the evolution experiment repeated, antagonism fails to emerge in five days and antibiotic synergy is maintained for longer. We therefore conclude that unless super-inhibitory doses are achieved and maintained until the pathogen is successfully cleared, synergistic antibiotics can have the opposite effect to that intended by helping to increase pathogen load where, and when, the drugs are found at sub-inhibitory concentrations.

## Abstract 2

Finding the most potent combinations of antibiotics in the lab can be a challenge if antibiotic interactions are not robust to evolutionary adaptation.

## Author Summary

We take an evolutionary approach to a problem from the medical sciences in seeking to understand how our knowledge of rapid bacterial evolution should shape the way we treat pathogens with antibiotic drugs. We pay particular attention to combinations of different drugs that are purposefully used to produce potent therapies. Textbook orthodoxy in medicine and pharmacology states one should hit the pathogen hard with the drug and then prolong the treatment to be certain of clearing it from the host; how effective this approach is remains the subject of discussion. If the textbooks are correct, a combination of two antibiotics that prevents bacterial growth more than if just one drug were used should provide a better treatment strategy. Testing alternatives like these, however, is difficult to do in vivo or in the clinic, so we examined these ideas in laboratory conditions where treatments can be carefully controlled and the optimal combination therapy easily determined by measuring bacterial densities at every moment for each treatment trialled. Studying drug concentrations where antibiotic synergy can be guaranteed, we found that treatment duration was crucial. The most potent combination therapy on day 1 turned out to be the worst of all the therapies we tested by the middle of day 2, and by day 5 it barely inhibited bacterial growth; by contrast, the drugs did continue to impair growth if administered individually.

## Introduction

Our arsenal of antimicrobials boasts a wide diversity of drugs and we continue to invest in the search for new ones [1]. Yet bacteria adapt so readily to their ambient environment that all antibiotics in clinical use have bacteria that resist them [2],[3]. A *Staphylococcus aureus* infection traced *in vivo* yielded over thirty *de novo* mutations from a 12-week therapy, each mutation conferring an increase in drug resistance [4]. With such a rapidly evolving foe and antibiotic discovery programmes waning substantially [3], determining optimisation principles that maintain the efficacy of the antibiotic repertoire already in our possession represents one of the keenest challenges confronting the scientific community.

And yet drug-resistance evolution has been called ‘conceptually uninteresting’ [5]. This view is the result of assuming a fixed timeline: a pathogen is treated with antibiotics, resistance traits emerge, sweep through the population and fix. The more efficient the drug, the greater selection for resistance and the sooner resistance fixes. The only mitigating action we can take is hit early, hit hard and kill drug-susceptible cells before they accumulate, so the old argument goes [6].

Bacteria are hardest hit by multi-drug combinations. Developed for over 70 years [1],[7],[8], combinations are key in our fight against microbes [9], viruses [10] and cancers [11]. Combinations said to be synergistic, where two drugs hit the pathogen much harder than each drug alone, are highly prized [1],[12],[13]. Indeed, the rapid deployment of synergistic antibiotics should, according to the same logic, be the fastest way of clearing a bacterium.

**Figure 1:**
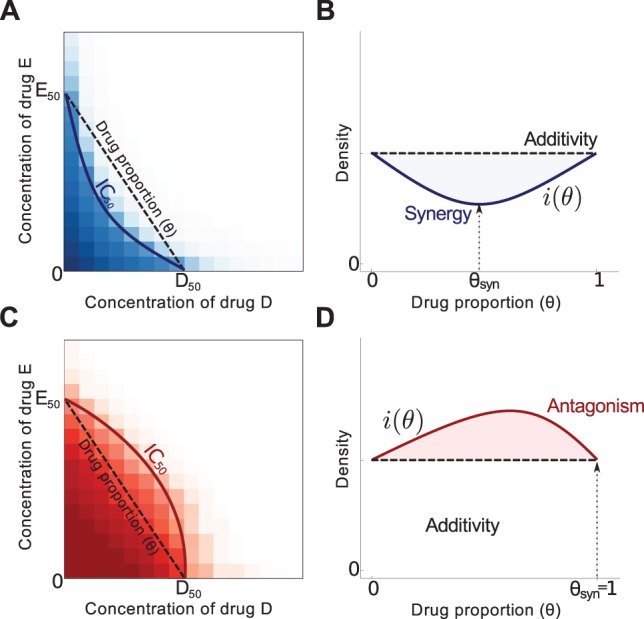
The drug interaction profile, *i*(*θ)*, as defined in Materials and Methods. The drug interaction profile is closely related to the two ‘checkerboard’ diagrams shown in (a) and (c). In a checkerboard, the concentration of both drugs is given on the *x* and *y* axes, bacterial growth inhibition (or population density or some other fitness measure) is then plotted on the *z* axis. The contour of all concentrations that reduce this measure by half is an *isobole* here denoted *IC*_50_ and figures (a) and (c) show two checkerboard plots viewed from above. Basal concentrations of both drugs that achieve the same inhibitory effect in this illustration are *D*_50_ and *E*_50_, *θ* then parameterises the equidosage line between these two values. The fitness measure evaluated along this line is shown in (b) and (d) and we define the degree of interaction based on this curve, this is *i*(*θ*). We say the interaction is *synergistic* when the drug proportion that minimises *i*(*θ*) satisfies 0<*θ*<1 as in (b), we denote the resulting value by *θ*_syn_. In (d) we observe *θ*_syn_ = 0 or *θ*_syn_ = 1, in this case the drugs are said to be *antagonistic* as *i*(*θ*) is maximised by some drug combination and minimised by the monotherapies.

To make our discussion more precise we say that a pair of bacteriostatic antibiotics of equal efficacy is synergistic if a 50-50 weighted combination of both drugs inhibits growth more than the two single-drug treatments when measured over one day of bacterial growth [8],[14]–[16]. (Strictly speaking, we ask this for all (*θ,(1−θ*))-combinations where *θ* is any value between 0 and 100%, not just 50-50, as shown in Figure 1.) With this definition we can formulate a null hypothesis, *H_0_*: *a synergistic drug combination also inhibits growth synergistically if the treatment lasts longer than a day*. Put differently, if the 50-50 combination treatment is more efficient than both single-drug monotherapies on the first day of treatment, it should also be more efficient on subsequent days to be deemed synergistic.

Any *in vitro* test of *H_0_* necessitates the use of antibiotic concentrations that support measurable population densities, the treatments we can use to test it are, as a result, necessarily constrained to a sub-inhibitory dosing regime. We must therefore question how relevant this study can be to antibiotic use *in vivo*, we argue that it is relevant for the following reasons. Drug interactions are often determined by one-day checkerboards and isoboles [17], like those illustrated in Figure 1, but by their very nature checkerboards only provide insight into the interaction inside the sub-inhibitory regime as isoboles can only be calculated if cells grow. Moreover, drug concentrations can sweep downwards from their highest values to sub-inhibitory concentrations during treatment ([18], Figure 1), repeatedly so for intermittent dosing regimens [19],[20]. The different diffusivities small antibiotic molecules exhibit in different tissue can create substantial inhomogeneities in concentration [21] resulting in a potential spatiotemporal mosaic of selection for resistance [18],[22] whereby treatment can reduce pathogen load in some, but not all, organs [23]. Indeed, spatial diffusion itself creates concentration gradients with rapid, super-exponential decay away from a point source. It is therefore essential to understand how antibiotic combinations mediate resistance at all dosages within this mosaic, including sub-inhibitory, particularly as resistance is known to be selected for at very low concentrations, well below the minimal inhibitory concentration [24].

**Figure 2:**
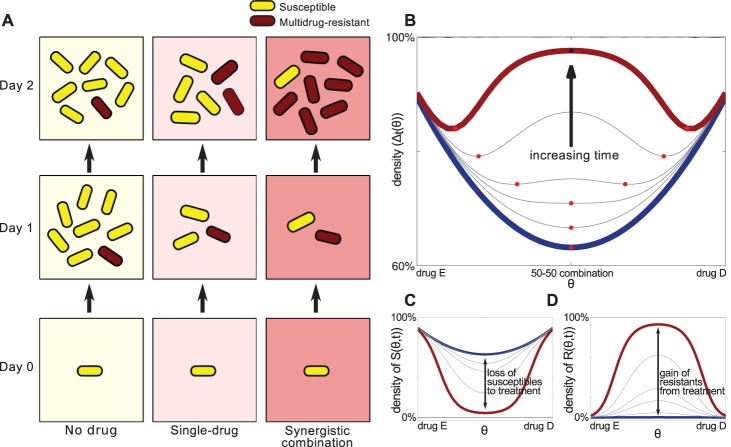
Smile-frown transition: a verbal argument and a toy mathematical model. (a) Synergistic drugs suppress drug-susceptible sub-populations (yellow cells) more than single-drug therapies however, this eliminates competitors of the drug-resistant red cells who grow more rapidly than the yellow cells would have done at weaker synergies. Thus greater synergy can increase population densities. (b) Solving Equation 1a–b and plotting population density against drug proportion shows that a short-term synergistic combination (blue) can maximise densities later (red). The red dots show the path of the optimal combination, note this idealised model is symmetric about *θ* = 1/2 but empirical data will not be. (c and d) The densities of drug-susceptible cells (*S* on the vertical axis in (c)) and resistants (*R* on the vertical axis in (d)) are shown at different times where, again, the blue line denotes a treatment of short duration and the red line denotes a longer treatment. The arrow in (c) represents the loss of *S* that occurs because of the drug whereas the arrow in (d) represents the analogous gain in *R*. For longer treatments the latter more than compensates for the former and by summing the red and blue lines in (b) and (c), respectively, we obtain the red and blue curves showing population density, Δ = *S*+*R*, in (a).

Now, we argue that treatments with the greatest short-term efficacy do not necessarily lead to the lowest bacterial densities later. A simple construction accounting for both density-dependent and frequency-dependent selection on drug resistance suffices to explain why. Consider three scenarios with two drugs, ‘A’ and ‘B’. A bacterium is either unchallenged by antibiotics, challenged with drug A only (or drug B only) or else treated with the optimally synergistic combination of both, as in Figure 2(a). The no-drug treatment sees the cells grow, to carrying capacity say, without selecting for drug-resistant phenotypes. The synergistic combination inhibits drug-susceptible cells optimally, better than the two monotherapies, and so, by the end of day 1, the lowest bacterial load of all is observed in this treatment. However, suppose some cells exhibit genetic or epigenetic adaptation conferring resistance; such cells may even have been present in low frequencies at the start of treatment. It is now in the synergistic line that drug-resistant phenotypes fare best as they have fewer competitors for the extracellular metabolites needed for growth.

To clarify how this might arise, imagine a population of bacteria with two subpopulations of drug-susceptible and resistant cells and suppose extracellular metabolites are shared equally among all the growing cells. As the growth of susceptibles is suppressed more at greater synergies, more metabolites become available for resistant cells in those treatments. However, resistant cells necessarily grow faster than susceptible cells do when the drugs are present, with a greater fitness difference at greater synergies. Thus the total population density can be increased by the synergy even when the number of drug-susceptible cells present is reduced. Now, if resistant cells are absent or at low frequencies at the beginning of treatment, the exposure to antibiotics must be long enough to allow the resistants to achieve densities comparable to the susceptibles and so the treatment duration then needs to be long enough for the claim in the previous sentence to be true. This process is illustrated in Figure 2.

This idea, known as ‘competitive release’ [25] has been tested in treatments of malaria *in vivo* using mice [5] where higher drug concentrations have been shown to select for higher parasite load but competitive release makes new predictions for antibiotic therapy, for combinations in particular. First, the optimal combination is not robust: the best way of deploying a drug pair depends on how long the treatment lasts. Second, and as a result, the favoured property of antibiotic synergy is not necessarily robust to adaptations that confer drug resistance. Not only will synergy decay with time, it can be lost completely and replaced with an antagonism because more potent combinations have paradoxically selected for larger bacterial load. Thus the theory of competitive release is not consistent with our null hypothesis and provides an evolutionary rationale for rejecting it.

A toy mathematical model captures the verbal argument completely and shows that synergy loss can be viewed as a form of tipping point. Imagine a bacterial population consisting of cells susceptible to both antibiotics at density *S(t)*, where *t* is time. Suppose there is a completely resistant phenotype, *R(t)*, and *μ* is the mean rate in a random Poisson process by which susceptible cells gain resistance. The dimensionless variable *θ* between zero and one controls the drug combination and k(*θ*) = 1+*θ*(1−*θ*) measures the efficiency of each combination at drug concentrations (*A*,*B*) = (*A_0_θ*, *B_0_*(1−*θ*)). Here *A_0_* and *B_0_* are normalising concentrations, chosen so that each drug achieves equal inhibitory effect at a defined time. Note that *k*(*θ*)is maximised when *θ* = 1/2. This value represents a 50-50 combination therapy whereby (*A*,*B*) = (*A_0_*/2, *B_0_*/2).

The toy model is the following logistic growth equation modified to include antibiotics:
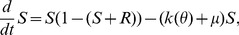
(1a)

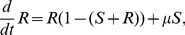
(1b)where 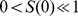 and *R*(0) = 0. We therefore begin with susceptible cells but no resistant ones. Figure 2(b) shows the population densities that result from this model, Δ*_t_*(*θ*) = *S*(*θ,t*)+*R*(*θ,t*), plotted as a function of *θ* for increasing values of time *t*.

For short times (Equation 1a–b) exhibits synergy because density is suppressed most by the combination where *θ* = 1/2, so the plot of Δ_t_(*θ*) has the convex, U-shaped ‘smile’ shown in blue in Figure 2(b). At later times, but only provided *μ*>0, the shape of the density profile changes and now density is *greatest* for the 50-50 combination and lowest for the ‘monotherapies’, where *θ* = 0 and *θ* = 1. So the plot of Δ*_t_*(*θ*) now exhibits a near-concave, W-shaped ‘frown’ consistent with antagonism having its maximal value at *θ* = 1/2, as shown in red in Figure 2(b). *Density is now maximised where before it was minimised*. We call the resulting passage from synergy to antagonism the ‘smile-frown transition’, referring to it on occasion as ‘synergy inversion’ because the convex, synergistic profile is inverted to form a near-concave, antagonistic one; this is a different notion of synergy inversion to the one in [26].

If we set *μ* = 0, thus preventing the modelled population from adapting to the drug, it then follows that Δ_t_(*θ*) has a synergistic profile at all times. In this case the 50-50 combination, represented by the value *θ* = 1/2, is the optimal combination for all times as it minimises population density, irrespective of treatment duration.

We tested the veracity of these theoretical predictions using an evolutionary functional genomics approach that combined evolution experiments using *Escherichia coli*, a genomic analysis, the genetic manipulation of an identified candidate resistance mechanism and quantitative mathematical modelling. This approach highlights the molecular mechanism that causes the synergy loss predicted by theory, whereas the theory alludes to the generality of the empirical results that we now describe.

## Results

### Evolution of a Family of Combination Treatments: Experimental Design

The above predictions are best tested *in vitro* where the drug interactions are well-understood and can be rigorously controlled. We therefore cultured *E. coli* K12 (MC4100) over a five-day period using a serial dilution protocol and sixteen different combination treatments of erythromycin (ERY, a macrolide) and doxycycline (DOX, a tetracycline), two bacteriostatic translational inhibitors with an established synergy [14]. The bacteria are first cultured for 24 h in liquid growth medium containing antibiotics at concentrations described below and, at the end of the 24 h period, a random sample of the bacteria is transferred using a standard plate replicator to inoculate fresh growth medium. This process is repeated to create a treatment lasting several days.

**Figure 3:**
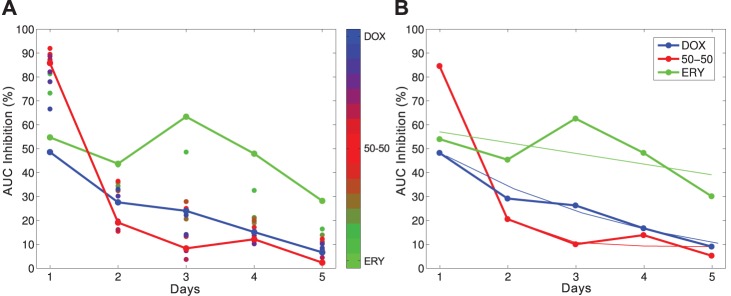
Dynamics of the optimal treatment: the greater the early inhibition, the faster efficacy decays and so the greater the resulting bacterial density. (a) Using an area under the curve (AUC) inhibition measure expressed as a percentage of growth without antibiotics, by design a combination of ERY and DOX is optimal on day 1 (red line, ‘50-50’) but an ERY monotherapy is optimal by day two (after the crossing points of the lines; c.f. Figure 4). The path of three extreme therapies are shown as lines, coloured dots represent the remaining thirteen treatments colour-coded from green (ERY) to blue (DOX). (b) Exponentially decaying datafits are superimposed upon three treatments from (a).

We began by choosing a pair of normalising, or ‘basal’, antibiotic concentrations, *D*_50_ and *E*_50_, in such a way that each DOX-only and ERY-only monotherapy achieved a 50% reduction in density when measured at 24 h relative to a zero-drug control (the basal concentrations *D*_50_ and *E*_50_ are the IC_50_ values of each drug). Each of the sixteen different treatments may therefore be described by a single pair of concentrations
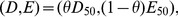
(2)where *θ* is the relative drug proportion. When combined in a 50-50 ratio at these doses, where *θ* = 1/2, a 90% reduction in bacterial growth at 24 h is achieved, greater than the 50% reduction achieved by each monotherapy (the data in Figure 3(a) (Day 1) supports this). We implemented 14 different combination treatments and two monotherapies at those basal doses with *θ* ranging in discrete values from 0 and 1/15 to 14/15 and then 1 (19 replicates per treatment; see Section 3.2 in Text S1).

The fixed drug proportion, *θ*, that minimises bacterial density from the sixteen implemented and determined empirically by culturing the bacteria for 24 h will be denoted by *θ*_syn_ in the following. This value between zero and one denotes the maximally synergistic combination treatment obtained after fixing the basal drug concentrations, as shown in Figure 1(b). The time-dependent optimal combination will be denoted *θ*_opt_ (*T*) (see Materials and Methods) and this value represents the combination of ERY and DOX that minimises density for a treatment of duration *T* hours. It follows by design that *θ*_opt_ (*T*) = *θ*_syn_ if *T* is small, less than 24 h, say.

**Figure 4:**
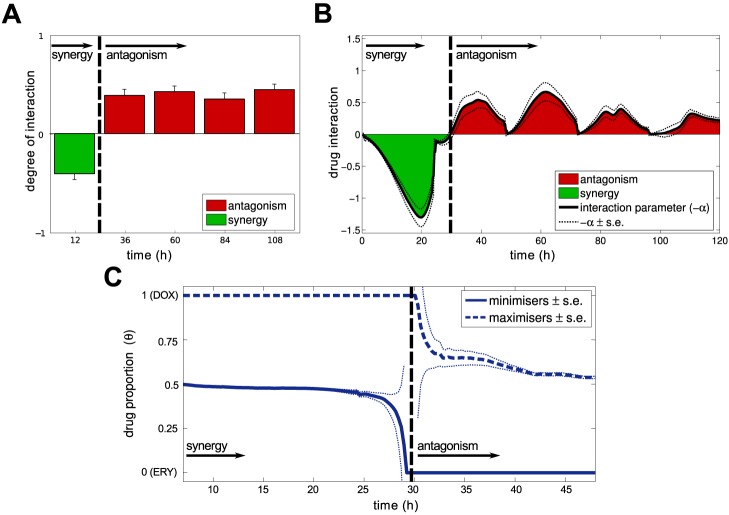
Drug interaction profiles are dynamic. (a) The degree of interaction, *I*(*T*) defined in Materials and Methods, is shown at different times from 12 h to 108 h: *I*(*T*) is negative for *T*≤24 h denoting synergy, but is positive for all *T*≥36 h denoting antagonism (vertical bars are s.e., 19 replicates). (b) A finer interaction measure than that used in (a), the degree of interaction obtained using the *α*-test defined in Materials and Methods produces a locus of drug interaction measures as a function of time. Consistent with (a), this measure changes sign, indicating a change of interaction near 30 h (note: −*α* is plotted). (c) The smile-frown transition resembles a phase transition when applying the *α*-test to *i*(*θ*,*T*) derived from MC4100 density data: the grey line shows the optimal drug combination that minimises *i*(*θ*,*T*), the red line shows the maximising combination. As the drug interaction profile ‘inverts’, the short-term optimal therapy shifts over a very short period to become the worst therapy beyond approximately 30 h. (The *y*-axis varies from *θ* = 0 (denoting an ERY-monotherapy) to *θ* = 1 (for a DOX-monotherapy), s.e. is shown as a pair of dashed lines.)

After calibrating concentrations *D* and *E* so that each drug has equal effect, so *θ*_syn_≈1/2 in practise as Figure 4(c) shows, the short-term optimal treatment is a 50-50 combination of both ERY and DOX. As a reflection of this, the day 1 data in Figure 3(a) then shows the 50% growth reduction obtained for each monotherapy, the 90% reduction for the maximally synergistic 50-50 combination in addition to the growth reduction for all the other combinations we tested. We can now test our null hypothesis by asking whether the drug combination that is optimal on day 1, 50-50 by design, is also optimal on subsequent days. [Disp-formula pbio.1001540.e001] Equation 1 makes a clear prediction: the best therapy on day 1 will be the *worst* later.

### Smile-Frown Transition: An Empirical Test

**Figure 5:**
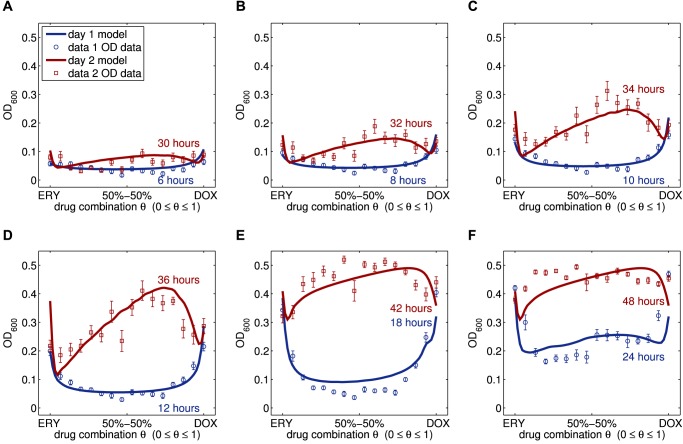
The smile-frown transition in empirical data and modelled bacterial densities. Shown are empirical and modelled bacterial densities (dots and lines, respectively) for 16 different drug proportions, denoted by *θ* and ranging from 0 (denoting ERY) to 1 (denoting DOX) on the horizontal axis. Population densities, measured as optical densities, are plotted against drug proportion are shown here in a panel of six time points with each blue and red datum 24 hours apart. The data was obtained using *E. coli* K12 (MC4100) challenged by erythromycin and doxycycline. The smile-frown transition described in the text occurs near 30 h at which point drug synergism is replaced by an antagonism. The model assumes multi-drug efflux is the only resistance mechanism and interpolates the discrete dataset to produce a series of continuous interaction profiles, as shown.

The first day's data exhibits synergism with the lowest short-term bacterial densities found for near 50-50 combinations of ERY and DOX, so *θ*_syn_≈1/2, this can be seen in Figure 5 (shown in blue). However, the subsequent population dynamics beyond day 1 lead to us to reject *H_0_* for Figure 5 (in red) shows they are consistent with the theory of competitive release and exhibit the smile-frown transition before 36 h have elapsed, as we now explain.

Consistent with the predictions of [Disp-formula pbio.1001540.e001] Equation 1, Figure 4(a) illustrates how the degree of interaction, *I*(*T*), defined in Materials and Methods, shifts from synergy (where *I*(*T*)<0; *t*-test, *df* = 19, *t*≈−6.13, *p*<0.0001,) to antagonism (where *I*(*T*)>0; *t*-test, *df* = 19, *t*≈6.83, *p*<0.0001) between 12 h and 36 h. The degree of interaction thereafter remains positive, denoting antagonism, until the end of the experiment. This is shown with more detail in Figure 4(b) where the dynamics of the interaction profile are shown on an hour-by-hour basis; this illustrates that the interaction changes at about 30 h.

Examining the apparent change in drug interaction more closely in Figure 5, at 12 h the interaction profile is synergistic (*α*-test, α = 0.61±0.05>0, *df* = 13, *t*≈11.22, *p*<10^−7^, *θ*_opt_(12*h*) = 0.49±0.01≈*θ*_syn_; see Materials and Methods and Section 4.3 in Text S1 for a description of the *α*-test) but combination treatments for which *θ*≈2/3 (estimated robustly using the *α*-test described in Materials and Methods as 0.65±0.04) yield the *highest* observed population densities by 36 h. As a result, the optimal combination has changed within two days from a 50-50 combination to an ERY monotherapy because the interaction profile is now antagonistic (*α*-test, α = −0.44±0.14<0, *df* = 13, *t*≈−3.05, *p*<0.0093, *θ*_opt_(36*h*)≈0≠*θ*_syn_; Section 4.3 in Text S1).

These data were produced for optical density measures of bacterial growth, but analogous results are obtained using different notions of fitness. Using an area under the curve measure of growth inhibition that accounts for both population sizes and growth rates (Section 4.2 in Text S1) Figure 3(a) shows drug efficacy approaches zero most rapidly for near 50-50 combination treatments. The same figure shows the optimal treatment has shifted in this measure too, to the ERY monotherapy within two days. For completeness, the smile-frown transition is also seen if we use colony-forming units to measure bacterial population densities (Section 7.4 in Text S1).

**Figure 6:**
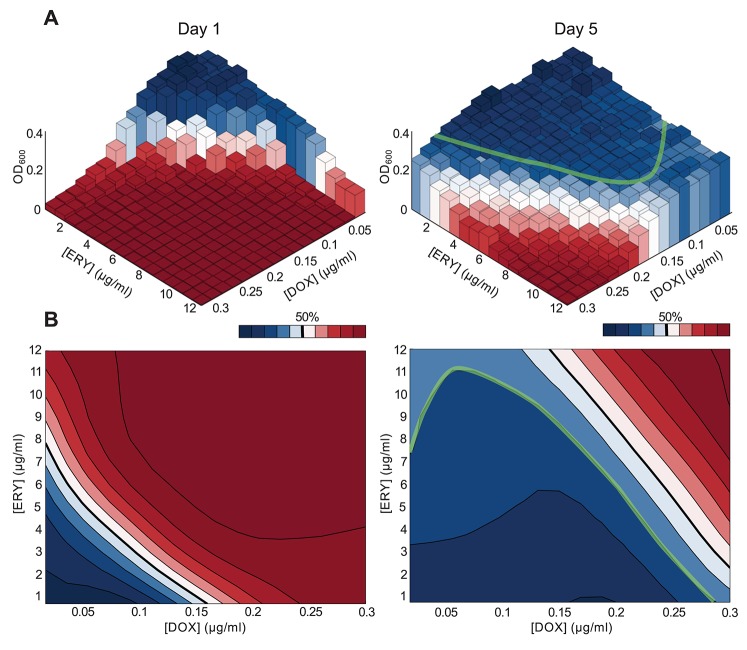
Drug checkerboards and isobolograms. (a) Empirical dose-response checkerboards show population density data on the *z*-axis versus drug concentration on the x and y-axes. This data was obtained by culturing *E. coli* sampled from the highly synergistic 50-50 environment at days one and five (the treatment with 4.8 µg/ml ERY and 0.08 µg/ml DOX), it corroborates the known synergism on day 1 and indicates the appearance of a more complex interaction by day 5. Note, 50% inhibition relative to the zero-drug control population is indicated by white blocks; (right) the 70% isobole is highlighted as a green line, indicating an interaction where one drug appears to suppress the other. (b) Isoboles (lines of equal inhibitory effect) are shown based on a numerical filter of the data from (a) (the fitting algorithm and code are described in [50]). Black lines correspond to isoboles in intervals of 10% inhibition, the darkest red areas illustrate increasing drug concentrations with inhibition towards 100%, the darkest blue areas denote inhibition closest to 0%. The white region denotes 50% inhibition.

As a further test for loss of synergy, dose-response checkerboards and isobolograms were produced using bacteria sampled from the highly synergistic *θ*≈8/15 treatment at the beginning of days one and five, both are shown in Figure 6. The earlier checkerboard is consistent with synergism whereas the latter checkerboard shows a progressing wave of increased resistance, with synergy at higher drug concentrations and a mixed interaction apparent at lower concentrations. Figure 6(a, right) shows isoboles at 60% inhibition that are suggestive of a suppressive interaction by day 4 in which doxycycline reduces the inhibitory effect of erythromycin. The white isobole of 50% inhibition in Figure 6(b) shows a shift from day 1 to 5 that indicates increased resistance of the population to both ERY and DOX (for controls that the antibiotics do not degrade significantly when stored at 4°C for several days see Section 3.2 in Text S1).

### Stabilising Synergy: A Genomic Prediction from a Mathematical Model

Having established the rapid loss of optimality of the most synergistic combination treatment, at which point the latter becomes the worst treatment of all, it is essential we understand the genetic basis of this change. So we first performed a test to determine whether increased drug resistance was the result of epigenetic adaptation (Section 3.2 in Text S1).

Samples each of the initially most synergistic drug treatment and the control treatment without drug were taken from the end of day 5 and cultured without antibiotics for a further 24 h. The resulting populations were then all subjected to the most synergistic drug combination for another 24 h. Consistent with a likely genetic basis to drug-resistance adaptation, samples from the short-term synergistic treatment still displayed greater AUC inhibition when measured relative to the no-drug control (Wilcoxon signed rank test, *W* = 92, *N* = 10, *p*<0.001).

Knowing such rapid adaptation has a genetic basis, our goal was to exploit the resistance mechanism and understand what organismal function, if suitably manipulated, could maintain antibiotic synergy for longer and so ensure the smile-frown transition does not occur so rapidly.

**Figure 7:**
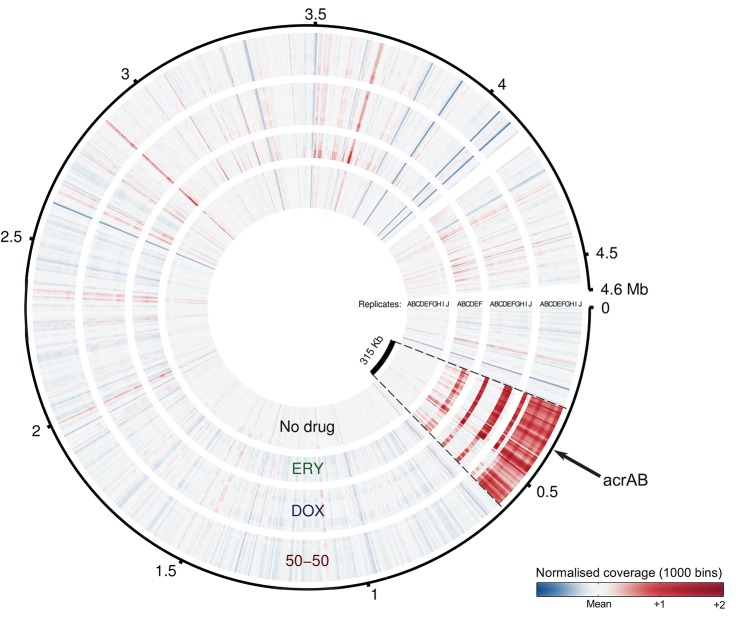
Coverage plots highlight the suspected duplication: a 2× increase in coverage suggests a gene duplication. A 315 Kb region of the *E. coli* K12 (MC4100) genome contains the *acrAB* operon and is highlighted in red. The region was not duplicated for treatments with no antibiotic (‘No drug’), it was duplicated for monotherapies (both ‘ERY’ and ‘DOX’) but was duplicated most often for combination treatments with the greatest synergy (‘50-50’). The outer ring (black line) indicates genome position, grey blocks encompass the different replicates of each treatment (replicates are marked with an alphabetic label) and the reddest regions are most likely to have been duplicated.

We therefore conducted a whole-genome sequencing study of independent biological replicates of both monotherapies and of the maximally synergistic treatment sampled at the end of day 5. The analysis revealed single nucleotide polymorphisms (SNPs) in most replicates modifying physiology, metabolism and drug resistance, including treatments with SNPs in *marRAB* and *acrR* (see Table 1, Figure 7, and Section 5.3 in Text S1). Indeed, the *mar* regulon is known to control a range of stress-responses in *E. coli* [27] including the multidrug efflux system *acrAB-tolC* [28].

**Table 1:** Overview of single nucleotide polymorphisms in the genomes of *E. coli* K12 (MC4100) that evolved within five days in erythromycin, doxycycline treatments or in a 50-50 combination of both.

Treatment	Gene	Polymorphic Sites	Frequency in Replicates	Annotation
**Doxycycline**	*marR*	7	0.5	Repressor of *marRAB* operon (controls antibiotic resistance and oxidative stress genes)
	*mdaB*	1	0.1	NADPH quinone reductase
	*agaS*	1	0.1	Tagatose-6-phosphate ketose/aldose isomerase
	*ascF*	1	0.1	Phosphotransferase system IIC components (carbohydrate transport)
	*eco*	1	0.1	Serine protease inhibitor
**Erythromycin**	*acrR*	1	0.2	*acrRAB* antibiotic transporter operon
	*ycbZ*	2	0.6	ATP-dependent protease
**50-50 combination**	*rcnA*	1	0.1	Membrane protein conferring nickel and cobalt resistance
	*evgS*	1	0.1	Hybrid sensory histidine kinase in two-component regulatory system

The number of polymorphic sites indicates how many independent nucleotide positions in the gene carry a SNP in at least one replicate, the frequency reflects the number of replicates where a polymorphism in the gene was found. The table only shows SNPs unique to the three treatments.

Rapid increases in resistance to antibiotics can occur when regions of the genome containing resistance genes are duplicated and whole-genome sequencing was proposed as a method to detect such duplications [29],[30]. Our analysis revealed 90% of the independent replicates in the most synergistic combination treatment had the same 315 Kb fragment duplicated, a region containing several efflux pumps including *acr* (Table 2, Section 5.4 in Text S1). The duplication was found in monotherapies too, but only in 30–40% of those treatments (3/10 replicates for DOX-only and 2/6 for ERY-only).

**Table 2:** Several antibiotic-binding and resistance genes are found in the 315 Kb genomic region duplicated most frequently in the 50-50 combination treatment, including the following genes and their annotations.

Start Position	End Position	Gene	Annotation
297113	298270	*ampH*	*β*-Lactam binding protein AmpH
370854	372626	*mdlA*	Putative multidrug transporter membrane/ATP-binding components
3383237	386386	*acrB*	Multidrug efflux protein
360871	363225	*lon*	DNA-binding ATP-dependent protease La
386409	387602	*acrA*	Multidrug efflux protein
387744	388391	*acrR*	Regulates the *acrAB* operon
72619	374400	*mdlB*	Putative multidrug transporter membrane/ATP-binding components
405459	406679	*fsr*	Putative fosmidomycin efflux system
470298	470630	*emrE*	Member of the SMR family of transporters. In *E. coli* this provides resistance against positively charged compounds including ethidium bromide and erythromycin; proton-dependent transporter exchanging protons for compound translocation (multidrug efflux protein).
564735	565946	*dacA*	Penicillin-binding protein; removes C-terminal D-alanyl residues from sugar-peptide cell wall
567184	568296	*mrdB*	Cell wall shape-determining protein
568299	570200	*mrdA*	Penicillin-binding protein

The duplication was therefore observed significantly more for the 50-50 combination treatment than in the ERY monotherapy (Fisher's exact test, *P*<0.035) and the DOX monotherapy (Fisher's exact test, *P*<0.02). In all 14 replicates where a duplication was detected, it was located between positions 274,201 bp and 589,900 bp. This region contains 293 genes, among which are 12 antibiotic resistance or binding genes, 32 transporter genes and 31 transposon-related genes (Appendix B in Text S1). Cross-resistance to antibiotics not used in the protocol is likely as three known multi-drug efflux systems and ampicillin degradation proteins are encoded within the duplicated region (Section 5.4 in Text S1 and Appendix B in Text S1). Such consistent, parallel evolution towards a 315 Kb duplication in all but one replicate of the 50-50 combination treatment strongly suggests, therefore, that genetic amplification of a multi-drug efflux pump is the adaptation that confers the multi-drug resistance phenotype we observe.

To test the stronger hypothesis that a drug efflux system could be responsible for synergy loss and the smile-frown transition, we first developed a system-specific, physico-genetics theoretical model (detailed in Section 6.4 of Text S1) in which cells may express a gene whose product can pump both antibiotics from the cell with no fitness or ATP cost. We assume the drugs have different affinities for the pump and the model encodes three phenotypes: drug-sensitive cells that do not express the efflux system, less sensitive cells that do and a third phenotype then possesses an additional efflux gene and expresses both. Figure 5 shows that the model successfully captures the first 48 h of data predicting that the rapid inversion of synergy that we observe empirically is consistent with the up-regulation and duplication of efflux genes.

Generalising this mathematical framework, we can show that the short-term optimal combination, represented by *θ*_syn_, and the time-dependent optimal combination *θ*_opt_(*T*) are close in general for a time that depends on the convexity of the drug interaction profile (Section 8.2 in Text S1). The two quantities are related as follows:
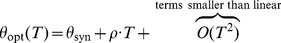
(3)where *T* is treatment duration, *ρ* is the divergence rate between the optimal treatment and maximal synergy; *ρ* may be positive or negative depending on how the bacteria adapt to each drug. The times
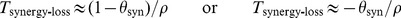
(4)are therefore approximations of the moment at which the optimal protocol is a monotherapy and no longer a combination. Figures 2(b), 3 and 5 all exhibit this phenomenon, but it can be seen most clearly in Figure 4(c) that shows the dynamical path taken by the best and the worst therapies. Analogous to a critical transition, a shift takes place at 30 h of treatment where the 50-50 therapy displaces the DOX monotherapy as the worst treatment. The synergistic treatment never recovers its previously favourable status rather, as Figure 3(b) shows in red, its performance continues to deteriorate exponentially.

The physico-genetics model predicts the drug interaction profile will be robust to changes in the duration of treatment, which can be interpreted as *ρ* being reduced in magnitude and so synergy maintained, if the efflux system were suppressed (Figure S16 in Text S1). This is analogous to setting *μ* = 0 in [Disp-formula pbio.1001540.e001] Equation 1 above.

**Figure 8:**
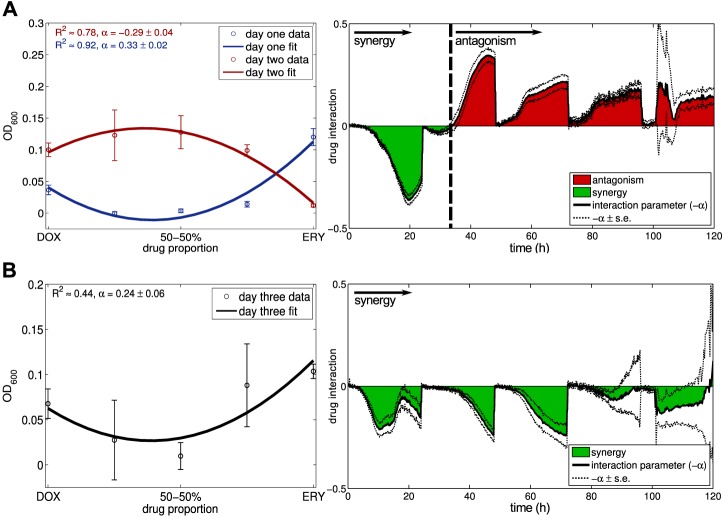
The deletion of one operon in strain AG100 stabilises antibiotic synergy. (a) The strain *E. coli* K12 (AG100) undergoes the smile-frown transition within two days (the value of *α* from the *α*-test is shown in the left figure and quoted ± s.e.; day-1 *α*-test at 18 h: *α*>0, *df* = 27, *t* = 14.84, *p*<10^−13^ with *θ*_opt_(18 *h*) = 0.04±0.01; day-2 *α*-test at 18 h: *α*<0, *df* = 27, *t* = −7.45, *p*<10^−7^). The right-hand plot shows a dynamic of the value of −*α* from the *α*-test through time with antagonism following synergy where the plot passes through zero, just as in Figure 4 for the strain MC4100. (b) *E. coli* K12 AG100A(Δ*acr*) does not exhibit the smile-frown transition by day 3 and the drug interaction is still synergistic then (*α*-test at 24 h on day 3: *α*>0, *df* = 27, *t*≈3.95, *p*<0.00052; see Section 7.2 in Text S1). The right-hand figure shows that the plot of −*α* from the *α*-test does not pass through zero at any time, the drug interaction is therefore stable and synergistic over the entire period of observation.

To test this prediction we repeated the original evolutionary protocol using two new *E. coli* strains: a wild-type strain AG100 and a mutant AG100A(Δ*acr*) [31]; we refer to Section 7 of Text S1 that details the minor differences between the first and now this evolutionary protocol. The latter strain differs from the former through a large deletion in *acrAB* that renders efflux systems that use the products of this operon, like *acrAB-tolC*, inoperable. As already observed using the *E. coli* K12 strain MC4100, AG100 soon exhibited the smile-frown transition, within 48 h according to Figure 8(a). In contrast, the mutant strain AG100A(Δ*acr*) that lacks *acrAB* continued to exhibit synergy until 72 h according to Figure 8(b), consistent with the prediction.

### Dose-Dependence: Higher Doses Amplify the Smile-Frown Transition

We now ask whether the synergy loss we observe is contingent on the choice of *D*_50_ and *E*_50_ as basal drug concentrations. For example, might synergy be maintained for longer if we were to increase the dosage of both drugs? We address this question with the following experiment.

We re-ran the drug-specific mathematical model (Section 6.4 in Text S1) at different dosages and repeated the evolutionary protocol using four different pairs of basal drug concentrations, chosen as follows. By analogy with (3) each new treatment can be represented by a pair of concentrations
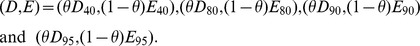
Empirically, we calibrated these four concentration pairs to produce a 40%, 80%, 90% and 95% reduction in growth relative to a zero-drug control by 18 h on day 1 for the 50-50 treatments (ones with *θ* = 1/2). We then subjected AG100 to treatments at each of the four basal dosages for a duration of five days using the drug proportions *θ* = 0,1/4,2/4,3/4, and 1.

**Figure 9:**
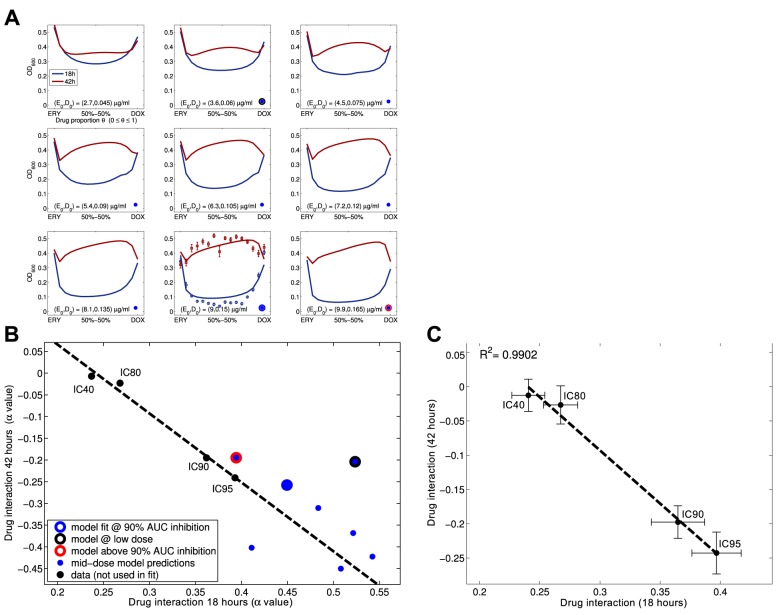
The stronger the synergy on day 1, the stronger the antagonism on day 2, both in models and data. (a) A theoretical model trained on prior predicts that the difference between 18 h synergy and 42 h antagonism will be greater at greater doses. (The prior training data is included in one of the panes and basal dosages are given within each pane.) (b) Predicted changes in interaction are shown as blue points that were determined using *α* values from the simulations in (a) above. Alongside these are the analogous *α* values from data in (c) which are black, the dashed line is the linear regression from (c). (c) The correlation between day 1 synergy and day 2 antagonism measured empirically at different basal dosages can be seen in this linear regression showing the interaction measure *α* at 12 h versus *α* at 48 h (see Materials and Methods for the definition of *α*; horizontal and vertical lines are s.e.). Labels denote the level of growth inhibition, 40, 80, 90 and 95%, observed at 18 h relative to a zero-drug control for each of four basal drug dosages.

The prior mathematical model made a quantitative prediction for this new protocol that is depicted in Figure 9(a): the greater the antibiotic dose, the greater the synergy observed on day 1 and the greater the resulting antagonism on day 2 (see also Figure 9(b)). These figures show the model predicts that synergy is maintained from the first day onwards only when the dosages are sufficiently low.

Figure 9(c) shows the results of this experiment are in quantitative agreement with the model. Indeed, the numerical values of day-one synergy and day-two antagonism are positively correlated in both the model and the resulting data (*R* ^2^ = 0.990, *F* = 145, *p*<0.0069) provided the antibiotic dose is sufficiently high in the former. Finally, we observe more rapid selection for resistance at higher doses in the sense that the greater the dose, the sooner the transition to antagonism (Section 7.3 in Text S1).

## Discussion

It is important to state that we, of course, exercise extreme caution when drawing parallels with *in vivo* infections where the immune response, the highly-organised spatial structure of the host, xenobiotic metabolism and the pharmacokinetics that result may substantially complicate antibiotic interaction dynamics. However, we also argue that *in vitro* evolutionary studies of bacteria allied to genome-wide analyses and mathematical modelling can play an important role in elucidating how antibiotic interactions change through time precisely because model systems like ours are so simple.

Drug interactions are subtle and synergy can be lost, and inverted, for reasons other than competitive release. Synergy must decay with time because of selection for drug-resistant alleles but it can be inverted when drugs degrade to produce non-antibiotic metabolites [26]. It is known that drug interactions can depend upon population heterogeneities because of differential pump expression between subpopulations [32], but cellular mechanisms not commonly associated with resistance might also force drug interactions to change with time. For example, a theoretical model was used to propose [33] that synergism and antagonism could be found simultaneously in a population of cancer cells due to metabolic adaptation in subpopulations, the so-called Harvey Effect [34]. To our knowledge, this theory has not been tested.

**Figure 10:**
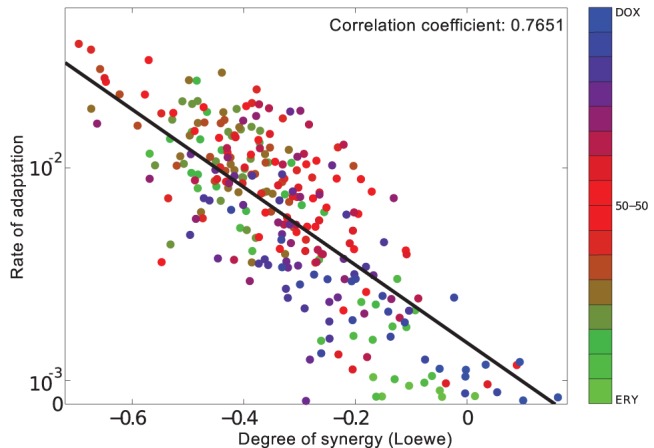
The greater the synergy, the more rapid adaptation is to treatment. This illustrates an entirely expected aspect of our data that corroborates a previous finding [14] on differences in rates of adaptation between antibiotic treatments using different drug pairs: selection for resistance is greater when treatments are more synergistic. The figure shows that our data also supports this idea (degree of interaction defined in Materials and Methods; rate of adaptation is defined in Section 4 of Text S1).

There are parallels with a prior study [14] that used antagonistic and synergistic antibiotic pairs to show that synergistic environments promote resistance more quickly than do antagonistic ones and the analogy of their result in our data is Figure 10. Their core argument, that single drug-resistance mutations have a greater fitness effect in more synergistic environments is applicable to our study and consistent with our findings. Unlike ours, however, that study did not address which treatments lead to the lowest or greatest bacterial loads.

Nothing of the molecular, multi-drug resistance mechanism is encoded within [Disp-formula pbio.1001540.e001] Equation 1 and despite its simplicity, this model may explain other phenomena. This includes the unreliability of antibiotic synergy assays such as checkerboards [35]–[37]. If a drug interaction assay were conducted with resistant cells in the inoculum [32] or if one emerged, irrespective of genetic mechanism, [Disp-formula pbio.1001540.e001] Equation 1 predicts synergy and antagonism could be reported for two replicates of the same checkerboard [37]. Indeed Figure 4(c) illustrates how the change from synergistic to antagonistic interaction can occur quickly and it is only when population density data is sufficiently well-resolved through time that a transition point from one to the other is found.

Our theoretical models are consistent with the smile-frown transition not being specific either to the drugs used or to the bacterium, any multi-drug resistance mechanism inactive in the absence of drugs that confers a fitness advantage in their presence may be sufficient (Section 8 in Text S1). However, while our data establishes that the duplication of a chromosomal multi-drug efflux operon is sufficient to observe the transition, this has been done for one Gram negative bacterial species and one drug pair.

Many questions therefore remain regarding the generality of our observations. Clinically-important pathogens are known to efflux drugs into extracellular space, or the periplasm, thus conferring resistance to a wide range of drugs in many species [38],[39]. As efflux has been observed both in clinical *Staphylococcus aureus* [40] and *Mycobacterium tuberculosis* (TB) [41] we ask whether synergy loss or the smile-frown transition might be observed in other bacteria. Relevant to this question is the study [35] of several clinical isolates of methicillin-susceptible and -resistant *S. aureus* (MSSA and MRSA) in which a combination of *vancomycin* and *rifampin* was variously reported as synergistic and antagonistic at 24 h and 48 h, with different interactions reported for both different strains and different drug concentrations. No mechanistic explanation has been attributed to this discrepancy and while this may not be at all related to efflux, the true nature of this important combination remains unclear [42].

What of drug combinations reliant on different mechanisms of synergy [43]? The duplicated genomic region illustrated in Figure 7 contains *dacA* with *β*-lactamase activity [44] and three efflux systems in addition to *acr*. Efflux of *fosmidomycin* by *far* [45], of *aminoglycosides* by *emrE* and of *fluoroquinolones* by *mdlAB* [39], all of which are found in the duplicated region (Table 2), indicates the smile-frown transition may also be relevant to other classes of antibiotics.

And would the transition still be observed if two target-altering, *de novo* mutations were needed for multi-drug resistance because there were no pre-existing chromosomal resistance mechanism that could be so rapidly duplicated? We have not been able to determine a pair of such mutations and so, by way of a partial response, we compared the duration for which synergy is maintained when an important chromosomally-encoded multi-drug pump is, and is not, present using data from *E. coli* strains AG100 and AG100A(Δ*acr*). Figure 8(a) shows that synergy is lost to antagonism in the former strain around 35 h but for the latter strain, the interaction only ceases to be significantly synergistic around 72 h, although significant antagonism is not observed thereafter. The latter strain, without *acr*, does therefore exhibit synergy loss but the smile-frown transition was not observed. However, in this case the interaction converges towards indifference in which one of the combination treatments maximises population densities by day 4 but without the smile-frown transition ever appearing (Section 7.2 in Text S1).

It has been suggested that the treatment of multi-drug resistant TB will be more successful if supplemented with efflux pump inhibitors (EPIs) [39],[46]. The present work suggests that if EPIs are used as an adjuvant to combination therapy they may prove beneficial by maintaining synergy for longer, although we have not conducted a direct test of this hypothesis using an EPI molecule.

We conclude that complementary theoretical and *in vitro* approaches agree that the optimal way of combining antibiotics depends on the duration of treatment. This could have been deduced from a simple engineering principle that complex adaptive systems cannot be controlled optimally using strategies that are constant through time (Section 8.2 in Text S1). The consequences of this principle for antibiotic combinations are dramatic and cause the emergence of what looks like antagonism from a synergism, rendering the supposed optimal combination the worst treatment of all within a day. So while it is axiomatic in theory [18] and demonstrable empirically [14] that drug resistance rises faster for more synergistic treatments, that the greatest antibiotic potency can also select for the highest bacterial densities has been overlooked.

## Materials and Methods

### Experimental Protocol

The protocol is a standard batch-transfer protocol used elsewhere [14] in the context of antibiotic treatments and described in detail in Section 3 of Text S1. Briefly: bacteria are cultured in liquid growth medium for 24 h in the presence and absence of different antibiotics and continually shaken. Optical density measurements are taken continually from where the inhibition due to treatment can be calculated relative to the growth observed in a control cultured without drugs. After each 24 h period has elapsed, the environment is sampled and approximately 1% of biomass transferred to fresh a environment that includes replenished growth medium and drugs. This process was repeated for 5 days.

### Quantifying Drug Synergy

There are many nonequivalent definitions of antibiotic synergy [8],[17],[47]–[49]. To ensure a precise quantification of drug interactions we use several consistent measures with different granularity derived with Loewe additivity as the key assumption. Suppose bacterial growth is measured over a fixed and short length of time, usually 24 h in the literature, although our measurements will be substantially longer. Population density is denoted by the function *B*(*D*,*E*) where *D* and *E* are extra-cellular drug concentrations, the number *B*(0,0) then represents density in a zero-drug environment. Assume each basal concentration, *D* and *E*, have been normalised to equal inhibitory effect, thus *B*(*D*,0) = *B*(0,*E*) = *rB*(0,0). The value 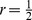 corresponds to the choice of IC_50_ for *D* and *E*, the concentrations denoted *D*_50_ and *E*_50_ in the text.

Quantification of the drug interaction begins with *i*, the interaction profile, where *i*(*θ*) = *B*(*θD*,(1−*θ*)*E*). Following Loewe additivity [8], *i* is said to be synergistic if, for all *θ* between zero and one exclusive, the effect of the drugs combined is greater than the sum of effects produced by each drug separately:
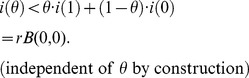
(5)This definition is described pictorially in Figure 1, Figures 1(b) and 1(d) are particularly relevant. Property (5) holds necessarily if *i*(*θ*) is convex (c.f. blue lines in Figures 2(b) and 5). When property (5) does hold it follows that *θ*_syn_, the maximally synergistic drug proportion that satisfies
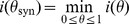
also satisfies 0<*θ*_syn_<1. Drug antagonism is said to occur when the reverse inequality applies in (5), this is necessarily the case if *i*(*θ*) is concave. The drug interaction is *additive* in this context if *i*(*θ*) is independent of *θ*.

Bacterial density is measured empirically over a time period of length T hours, so we now introduce T into the definition of *B*. Denote density by *B*(T;*D*,*E*) and re-write *i* as *i*(*θ*,T) to account for the change. The time-dependent optimal combination, *θ*_opt_(T), then satisfies
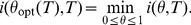
(6)It follows by definition that *θ*_opt_(T) and *θ*_syn_ are equal when T = 0 and are therefore also close for small T, Equation 3 describes the rate of divergence between the two.

If we define the dimensionless interaction profile
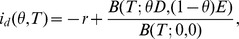
the degree of interaction, *I*(T), is given by the mean interaction taken over the relevant drug combinations:
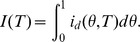
Negative *I*(T) denotes synergy, positive *I*(T) denotes antagonism.

A measure of the convexity and concavity of *i*(*θ*,T) obtained by fitting a quadratic, 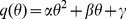, can be used to assess the drug interaction. Significant *positivity* (obtained using a t-test) of *α* indicates *synergy*, negativity indicates antagonism; Section 7 in Text S1 gives further information on the use of this test. If the density data is significantly nonlinear as a function of *θ*, meaning *α*≠0, the fitted quadratic can be used to robustly estimate the drug proportion that maximises bacterial density at each time. This proportion is given by one of *θ* = 0,1 or −*β*/(2*α*) depending on which value is the lowest of *q*(0), *q*(1) or *q*(−*β*/(2*α*)). Provided −*β*/(2*α*) lies between 0 and 1, an approximate upper bound on the confidence interval for this optimal value can be found from a t-test that returns confidence intervals for *α*, *β*, and *γ*. Throughout we will refer to the test described in this paragraph as the ‘*α*-test’ and it is implemented using the regression facilities in the Statistics Toolbox of MATLAB.

## Supporting Information

Text S1 **Supporting information.** This file includes nine sections and two appendices: 1. Introduction: hit early, hit hard? 2. Drug interaction profiles: synergy and antagonism. 3. Experimental evolution in a two-drug environment: methods. 4. Experimental evolution in a two-drug environment. 5. Analysis: whole genome sequencing. 6. Analysis: a mathematical model consistent with data. 7. Validating the theory: testing the smile-frown experiment with an *acrAB* knockout. 8. Optimal drug combinations are not constant: an analysis. 9. Final comment: single cell synergy and population synergy. Appendix A: Parameter values. Appendix B: Genes annotated in the duplicated regions.
(PDF)
