# Supplementary material for: When the Most Potent Combination of Antibiotics Selects for the Greatest Bacterial Load: The Smile-Frown Transition
Source: PLoS Biol. 2013 Apr 23;11(4):e1001540. doi: 10.1371/journal.pbio.1001540 (PMC3635860; doi:10.1371/journal.pbio.1001540)
Supplement: Text S1 — Supporting information. This file includes nine sections and two appendices: 1. Introduction: hit early, hit hard? 2. Drug interaction profiles: synergy and antagonism. 3. Experimental evolution in a two-drug environment: methods. 4. Experimental evolution in a two-drug environment. 5. Analysis: whole genome sequencing. 6. Analysis: a mathematical model consistent with data. 7. Validating the theory: testing the smile-frown experiment with an acrAB knockout. 8. Optimal drug combinations are not constant: an analysis. 9. Final comment: single cell synergy and population synergy. Appendix A: Parameter values. Appendix B: Genes annotated in the duplicated regions. (PDF) [file pbio.1001540.s001.pdf]

WHEN THE MOST POTENT COMBINATIONS OF ANTIBIOTICS SELECTS FOR THE GREATEST BACTERIAL LOAD: THE SMILE-FROWN TRANSITION

R. PENA-MILLER, D. LAEHNEMANN, G. JANSEN, A. FUENTES-HERNANDEZ,
P. ROSENSTIEL, H. SCHULENBURG AND R. BEARDMORE

Table of Contents

1. Introduction: hit early, hit hard? 2
1.1. Toy mathematical models: are synergistic drug combinations optimal? 2
2. Drug interaction profiles: synergy and antagonism 4
3. Experimental evolution in a two-drug environment: methods 6
3.1. Materials 6
3.2. Experimental protocol 6
4. Experimental evolution in a two-drug environment 8
4.1. Trade-off between rate of adaptation and degree of synergy 8
4.2. A measure of antibiotic efficacy: AUC inhibition 9
4.3. Smile-frown transition in the data 9
4.4. A corollary of the smile-frown transition: when a monotherapy is optimal 10
5. Analysis: whole genome sequencing 11
5.1. Genetic adaptation in evolved populations of *E. coli* K12 (MC4100) 11
5.2. DNA extraction and sequencing 11
5.3. Data analysis 11
5.4. Summary of results 12
6. Analysis: a mathematical model consistent with data 14
6.1. A core growth inhibition model: capturing dose-response curves 14
6.2. Modelling synergistic drug interactions 16
6.3. A mathematical model structure to mimic the experimental protocol 17
6.4. A drug efflux model 17
6.5. Theoretical prediction: synergy is stabilised if the efflux pump is suppressed 19
7. Validating the theory: testing the smile-frown experiment with an *acrAB* knockout 20
7.1. Additional experimental details 20
7.2. Outcome: synergy is maintained for longer with the pump knockout 21
7.3. Dose-dependence: higher doses amplify the smile-frown transition 23
7.4. Colony forming units (CFUs) as a density measure 25
8. Optimal drug combinations are not constant: an analysis 25
8.1. Mathematical definition of synergy 26
8.2. Generically, the optimal combination changes through time 27
9. Final comment: single cell synergy and population synergy 28
References 29
Appendix A. Parameter values 31
Appendix B. Genes annotated in the duplicated region 32

1. Introduction: hit early, hit hard?

Is it true that pathogen load decreases as antibiotic efficacy increases? This relationship appears self-evident. Moreover, as antibiotic efficacy can be enhanced by appropriately combining two or more drugs into a single therapy, it would also appear self-evident that the most effective way of deploying antibiotics is in combination. The combinations of antibiotic drugs preferred in clinical practise are the so-called *synergistic combinations* in which one antibiotic enhances the killing effect of another and *vice versa* [1]. Indeed, the *search for synergy* [2] is the idea that we can screen for small antibiotic molecules that enhance each other's killing effect [3] and use these as the basis of antibacterial treatments in the clinic. The century-old rationale for deploying antibiotics is simple, *hit early and hit hard* [4] and the use of synergistic combination therapies is a logical consequence of assuming the validity of this rationale.

Hit early, hit hard provides a rational approach to antibiotic use even when accounting for *de novo* drug-resistance evolution. If an allele confers increased drug resistance, it will sweep through a population after the antibiotic has been administered. The more drug we apply, the greater the rate of sweep and so the earlier the allele will fix in the pathogen population. The remedy, therefore, is to prevent resistance from arising in the first place and to apply as much drug as possible, as early as possible to keep pathogen load to a minimum for this minimises the probability of the novel allele arising in the first place.

1.1. Toy mathematical models: are synergistic drug combinations optimal?

Let us test the robustness of this argument using a very simple mathematical model. Imagine a single bacterial strain growing in a competitive environment where the resources needed for growth are scarce. Suppose $S(t)$ denotes the density of a bacterial population per unit volume at time t and, in the absence of drug, the population dynamics of the bacterium follow the following logistic equation:

$$\frac{d}{dt}S = S(1-S), \qquad 0 < S(0) \ll 1. \tag{1}$$

The dynamics of equation (1) see the population grow to carrying capacity at some rate, here both these parameters have been set to be unity. We now wish to clear the bacterium from its environment and so we introduce an antibiotic, with killing efficacy a:

$$\frac{d}{dt}S = S(1-S) - aS, \qquad 0 < S(0) \ll 1. \tag{2}$$

Suppose we now ask for the response time, t_{50}, at which the bacterium achieves half its final carrying capacity, so that $S(t_{50}) = 1/2$. Due to the nature of the model, it is a trivial fact that the best therapy, the one that maximises t_{50} is the one with the largest possible value of a. Thus equation (24) is consistent with the hit early, hit hard rationale.

Now suppose we modify equation (24) by including a second drug. We suppose it has the same killing efficacy as the first, but when we combine the two their total killing efficacy is increased. So, we now adapt the term 'a' to be a function of a variable θ between 0 and 1 such that $a(0) = a(1) > 0$. The idea here is that θ controls the fraction of each drug used in a combination treatment, so $\theta = 0$ represents the use of one drug only and $\theta = 1$ represents the use of the other drug. The quadratic function of θ,

$$a(\theta) = 1 + \theta(1-\theta),$$

suffices to mimic two synergistic drugs of equal killing effect in the following model:

$$\frac{d}{dt}S = S(1-S) - a(\theta)S, \qquad 0 < S(0) \ll 1. \tag{3}$$

Again, it is trivial that t_{50} is minimised by solutions of (3) for the '50-50' combination therapy, namely the treatment that mixes both drugs in equal amounts, so that $\theta = 1/2$. Thus, the structure of the model (3) is again consistent with hit early, hit hard rationale.

Now suppose we invoke a further change to our model and explicitly include drug resistance adaptation. To this end, without specifying an explicit physical mechanism supporting drug resistance at this point, we suppose that a second phenotype, $R(t)$, can be found in the population that is identical to the drug-susceptible wild-type, S, but which is completely drug resistant:

$$\frac{d}{dt}S = S(1-(S+R)) - a(\theta)S - \mu S, \qquad 0 < S(0) \ll 1, \tag{4a}$$

$$\frac{d}{dt}R = S(1-(S+R)) + \mu S, \qquad R(0) = 0. \tag{4b}$$

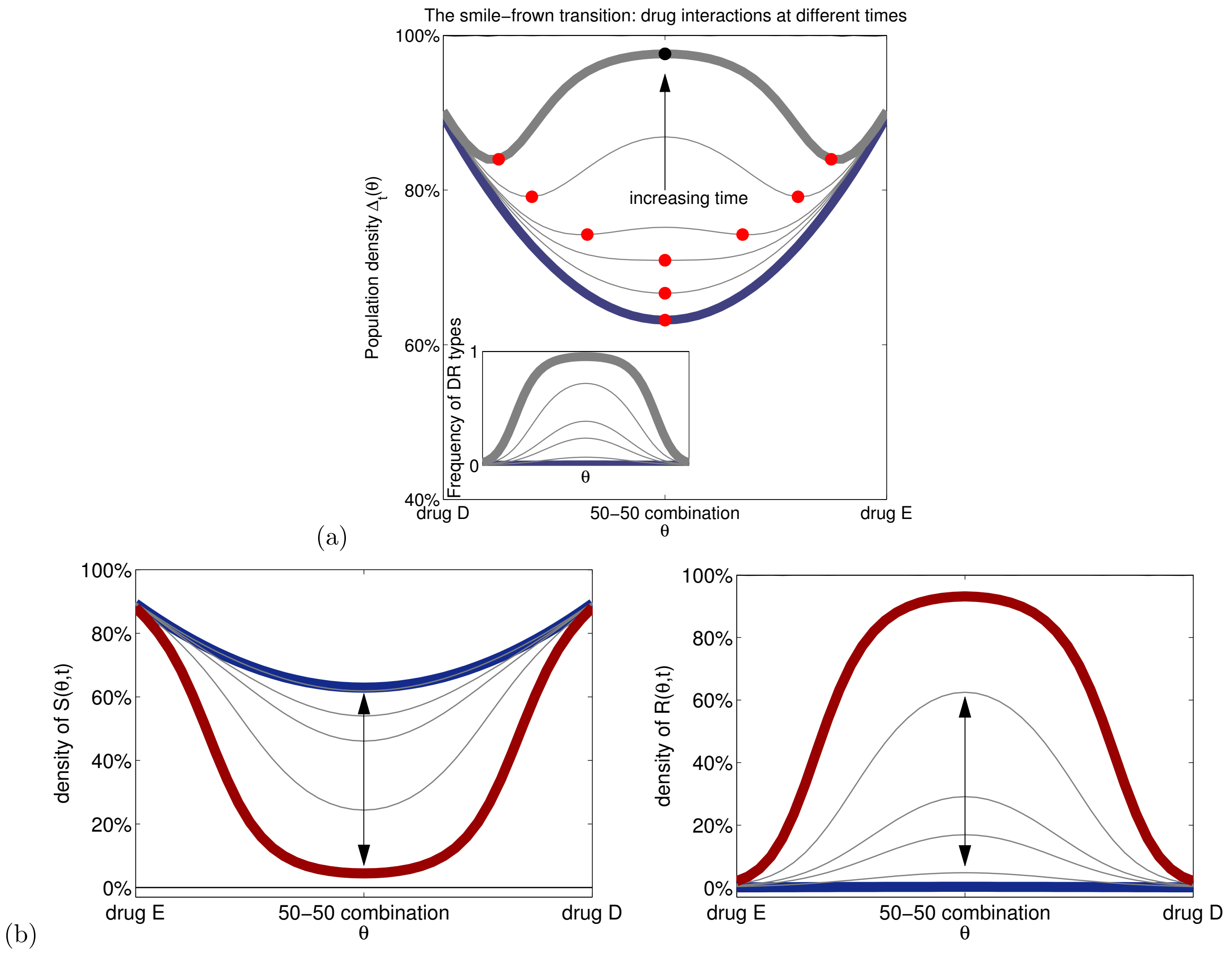

supplement fig. S1 – (**Competitive Release and the Smile-Frown Transition**) (a) Population density (as a fraction of carrying capacity) on the y axis is plotted against the drug combination parameter, θ, on the x axis for treatments of different duration. Two drugs are used, 'D' and 'E', whose synergy is apparent over treatments of short duration because the population density is minimised where $\theta = 1/2$ (the red dot situated on the thick blue line). However, there comes a future time for which density is maximised for this treatment (the black dot on the thick grey line). The red dots show the path of the treatments of minimal density as time progresses: they start as combination therapies but eventually become monotherapies. The inset shows the frequency of drug-resistance in the population at the same times (denoted 'Frequency of DR types' on the y axis), illustrating that drug resistance sweeps fastest where synergy is greatest. In both diagrams, θ ranges from zero (drug D) to one (drug E). (b) The densities of S and R are shown at different times, the thick blue line denoting a treatment of short duration and a thick red line denoting a longer treatment. The black arrow denotes the loss of S that occurs because of the drug as the treatment proceeds and it has the same length in both left and right figures. However, the right-hand figure shows that the gain in density of R for that length of treatment more than compensates for the loss of S due to the drug.

Here $\mu > 0$ is the constant rate at which the drug-susceptible phenotype gains resistance and, to begin with, we assume that there are no resistant bacteria in the population. We can use different optimality criteria to understand how to deploy drugs in equation (4), the most natural one is to minimise population density, so we denote the population density by

$$\Delta_t(\theta) = S(t) + R(t), \tag{5}$$

noting that the latter depends on θ implicitly.

The question we now ask is for what value of θ is $\Delta_t(\theta)$ minimised? We know by design that $\theta = 1/2$ is the value corresponding to the most synergistic treatment and that this value also minimises population density for a short period of time. But what happens as time increases? Supplement fig. S1 answers this question.

It shows that there comes a future point at which the most synergistic treatment ceases to be optimal and, in fact, a short time later this drug combination becomes the worst treatment of all. Somewhere between these two times, the monotherapies reduce population density below what can be achieved by combining the drugs.

The inclusion of multi-drug resistance thus complicates the picture. For example, it not true for (4) that the optimal therapy hits the drug-susceptible bacteria as hard as possible. Rather, the optimal therapy takes two forms: a near 50-50 combination consisting of both drugs at equal concentration for short treatments, but a monotherapy of either drug is better for longer treatments.

2. Drug interaction profiles: synergy and antagonism

In order to test these theoretical predictions, the following section will describe a laboratory-based microcosm in which different strains of *E.coli* K12 are challenged by the antibiotics erythromycin and doxycycline. However, before we can understand the outcome of those experiments, we need to define some common terminology and present basic data analysis tools we need to understand how different combinations of antibiotics interact.

In the remainder, we will assume that a population of bacteria is cultured in the presence of two antibiotics at extracellular concentrations denoted by two variables, D and E; the latter are called *basal drug concentrations.* Bacterial population density, for instance measured in units of optical density (OD or $\mathrm{OD}_{600\mathrm{nm}}$) in a shaken culturing device will depend on the concentrations of both these drugs. If t denotes time, density will be denoted by the drug-dependent time-series $B(t; D, E)$ and as cells are cultured for a fixed amount of time, T hours say, it follows that $0 \leq t \leq T$. In what follows, our fitness measure of a bacterial population will be the optical density determined empirically from a lab-based experimental microcosm, but other measures of fitness could, equally, be used.

A fair comparison of the efficacy of a drug combination necessitates that each basal drug concentration, D and E, is normalised to achieve equal inhibitory effect over some period of time. We will therefore assume that each single-drug monotherapy achieves a factor-r reduction in bacterial density relative to a drug-free environment by the end of the experiment, thus

$$B(T; D, 0) = B(T; 0, E) = rB(T; 0, 0).$$

As in practise we will normalise the basal drug concentrations to achieve 50% inhibition with respect to the null antibiotic control in a growth assay lasting twenty-four hours, in the remainder we have in mind the specific value $r = 1/2$. For convenience, we therefore denote by D_{50} the basal concentration of the first drug and assume that

$$B(T; D_{50}, 0) = \frac{1}{2}B(T; 0, 0).$$

Similarly, E_{50} is the concentration of the second drug, E, and it satisfies $B(T; 0, E_{50}) = B(T; 0, 0)/2$ too.

Using D_{50} and E_{50} as basal drug concentrations, we introduce a drug combination parameter that allows us to combine both drugs whilst maintaining a constant effective dosage. This parameter, denoted throughout by θ, takes values between zero and one inclusive and it can be used to represent any drug combination along the *equidosage line* (see supplement fig. S2). For each value of θ, the actual concentration of drug deployed to the environment of the bacteria is then $\theta \cdot D_{50}\, \mu g/ml$ of drug D and $(1-\theta) \cdot E_{50}\, \mu g/ml$ of drug E.

The basal concentrations will be fixed throughout, the only freedom permitted in terms of determining optimal drug therapies will be through the parameter θ. Indeed, the value of θ that gives rise to the lowest bacterial density will be deemed the optimal combination. Our definitions are designed to ensure a fair comparison between different treatments, we are therefore not at liberty to deploy antibiotics at arbitrarily high dosages and deem those better therapies.

The degree of interaction between drugs is usually defined in terms of the deviation from a neutral interaction, derived either using Bliss independence or Loewe additivity (for an extensive discussion of this topic see [2]). Here we will use the latter and define synergism based on the so-called interaction profile

$$i(\theta) = B(T; \theta D, (1-\theta)E),$$

defined for $0 \leq \theta \leq 1$.

Definition 1 (the Loewe drug interaction profile: $i(\theta)$; synergy, antagonism and additivity)**.** *A drug interaction is said to be* synergistic *if, for all θ between zero and one exclusive, the effect of the drugs combined is greater than the sum of effects produced by each drug separately:*

(6) $$i(\theta) < \theta \cdot i(1) + (1-\theta) \cdot i(0).$$

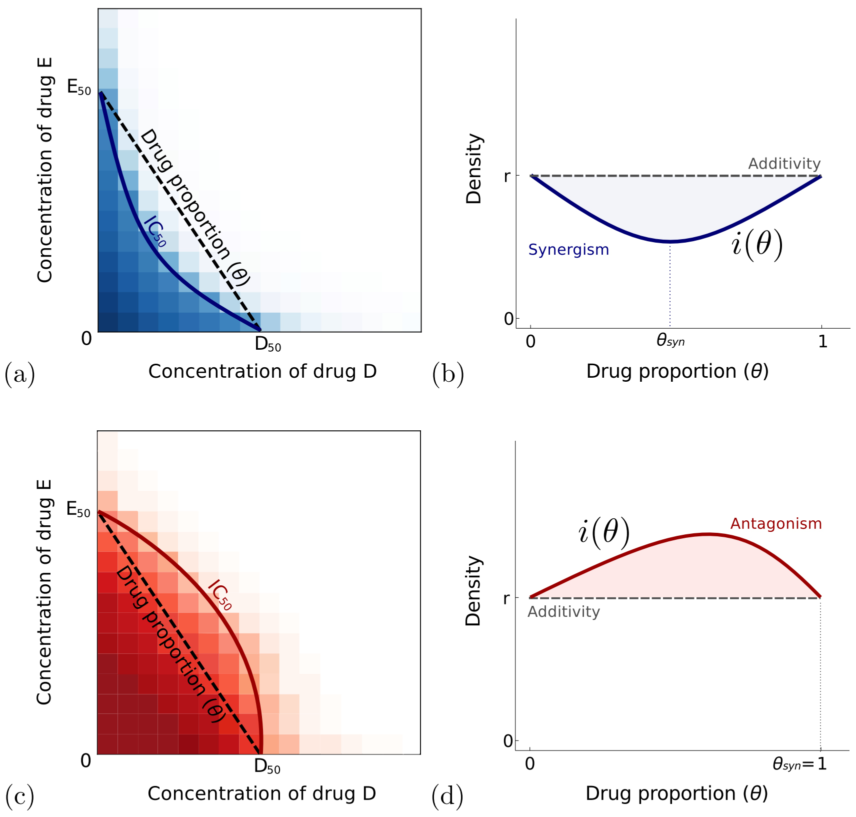

supplement fig. S2 – The drug interaction profile is related to a 'checkerboard' diagrams shown in (a) and (c). In the latter, the concentration of both drugs is given on the x and y axes, bacterial growth inhibition (or population density or some other fitness measure) is then plotted on the z axis. The contour of all concentrations that reduce this measure by half is an *isobole* here denoted IC_{50} and figures (a) and (c) show two checkerboard plots viewed from above. Basal concentrations of both drugs that achieve the same inhibitory effect in this illustration are D_{50} and E_{50}, θ then parameterises the equidosage line between these two values. The fitness measure evaluated along this line is shown in (b) and (d) and we define the degree of interaction based on this curve, this is $i(\theta)$. We say the interaction is *synergistic* when the drug proportion that minimises $i(\theta)$ satisfies $0 < \theta < 1$ as in (b), we denote the resulting value by θ_{syn}. In (d) we observe $\theta_{\text{syn}} = 0$ or $\theta_{\text{syn}} = 1$, in this case the drugs are said to be *antagonistic* as $i(\theta)$ is maximised by a drug combination.

It follows by construction that $i(1) = i(0) = rB(T; 0, 0)$ and so (6) asks that treatment efficacy is greatest when drugs are combined. Note that inequality (6) is necessarily satisfied if $i(\theta)$ is a convex function of θ.

The most synergistic drug combination is denoted by the value θ_{syn}, this is the drug proportion that satisfies

$$i(\theta_{syn}) = \min_{0 \le \theta \le 1} i(\theta); \tag{7}$$

it must follow that $0 < \theta_{syn} < 1$ for a synergistic interaction. The drug interaction is said to be antagonistic *if the reverse inequality applies in (6) and in this case $\theta_{syn} = 0$ or $\theta_{syn} = 1$; this will apply if $i(\theta)$ is a concave function of θ. Under these assumptions the drug interaction is said to be* additive *if $i(\theta)$ is a constant and therefore independent of θ.*

Definition 1 is quite standard but the following discussion is not commonly undertaken when antibiotic interactions are discussed: bacterial inhibition due to drugs is measured over a time interval of total length T hours. We therefore introduce time, T, explicitly into the interaction profile and write the latter as a time-dependent interaction $i(\theta, T)$. The time-dependent optimal combination, $\theta_{\text{opt}}(T)$, is then the drug proportion that satisfies

$$i(\theta_{\text{opt}}(T), T) = \min_{0 \le \theta \le 1} i(\theta, T) \tag{8}$$

and it follows by definition that $\theta_{\text{opt}}(T)$ and θ_{syn} are equal for small values of T.

From the dimensionless interaction profile

$$i_d(\theta, T) = -r + \frac{B(T; \theta D, (1-\theta)E)}{B(T; 0, 0)},$$

we define the following *degree of interaction* at T hours, $I(T)$, a value given by the mean of drug interactions over all possible drug combinations:

$$I(T) = \int_0^1 i_d(\theta, T) d\theta. \tag{9}$$

There are different definitions of drug synergism in the literature and Definition 1 is given using Loewe additivity as its starting point. In order to provide consistency of results across different possible definitions of drug interaction, we will also use the following synergy measure defined in terms of Bliss independence [2].

Definition 2 (the Bliss interaction profile: $i_b(\theta, T)$)**.** *The time-dependent Bliss interaction profile is given by*

$$i_b(\theta; T) = \frac{B(T; \theta D, 0)}{B(24; 0, 0)} \cdot \frac{B(T; 0, (1-\theta)E)}{B(T; 0, 0)} - \frac{B(T; \theta D, (1-\theta)E)}{B(T; 0, 0)}; \tag{10}$$

positive values of $i_b(\theta; T)$ correspond to a synergistic interaction while negative values correspond to antagonism.

3. Experimental evolution in a two-drug environment: methods

Now the basic numerical tools needed to define and analyse drug interactions have been detailed, we continue with a description of an evolutionary experiment in which a bacterium is challenged by drugs that interact synergistically. The experimental methods are based on those of a previous study [5], detailed as follows.

The following serial dilution protocol was implemented. A shaken microtiter plate with liquid minimal growth medium is inoculated with a small density of bacteria. In addition to the limiting carbon source and other nutrients needed for growth, each well in the plate contains a combination of antibiotics with basal concentrations D and E at a proportion determined by the parameter θ. Cells are cultured for $T = 24$ hours after which a small and fixed volume is taken from each well and transferred to a second plate which contains fresh liquid growth medium and the same combination of antibiotics. The repetition of this process defines a serial transfer experiment. It is essential to note that the supplied concentrations of extracellular resources and drugs and each drug proportion, defined as θ above, are *identical* in every transfer. This allows us to study the adaptation of a bacterial population in a fixed drug environment.

3.1. **Materials.** *E. coli* K12 (MC4100) (from the Coli Genetic Stock Center: `http://cgsc2.biology.yale.edu/Strain.php?ID=9973`) was used and inoculation cultures were started from the same single colony for all the experiments described below.

Experimental populations were cultured in M9 medium with the following concentrations: Part A: 350 g/LK_2HPO_4, 100 g/LKH_2HPO_4; Part B: 29.4 g/L Trisodium citrate, 50 $g/L(NH_4)_2SO_4$, 5 $g/LMgSO_4$. Parts A and B were 50x stock solutions in deionized water, sterilised by autoclaving. For M9 minimal medium they were diluted in water accordingly with 0.2% glucose and 0.1% casamino acid added as nutrients.

The antibiotics used are erythromycin (Sigma-Aldrich, Product #856193) and doxycycline (Sigma-Aldrich, product #D9891). Liquid stocks were prepared from powder stocks at $50mg/ml$ in ethanol for erythromycin and at $5mg/ml$ in deionized water for doxycycline (afterwards filter sterilised) and frozen at -20°C. All dilutions were prepared in M9 growth medium and then stored in the fridge at approx. 4-8°C.

3.2. **Experimental protocol.** Experiments were conducted in 384-well plates with $100\mu l$ liquid volume. Growth medium and antibiotics were distributed over the plates fully randomised by a pipetting robot (Tecan Freedom Evo) to exclude gradient effects (for typical randomisation patterns, see supplement fig. S3). Plates were inoculated and transferred with a 384-well replicator. Experiments were conducted at 30°C, density measurements were taken in a shaken plate reader (Tecan Genios).

The full protocol consisted of three sets of experiments:

(Ex. 1) **Single-drug dose-response experiments to calibrate equal inhibitory effects.**
Preliminary experiments were performed to determine dose-response relationships for erythromycin and doxycycline, both are shown in supplement fig. S11. This data determines the MIC and the IC_{50} for both drugs.

(Ex. 2) **Five day serial dilution experiment with different multidrug combination treatments.**

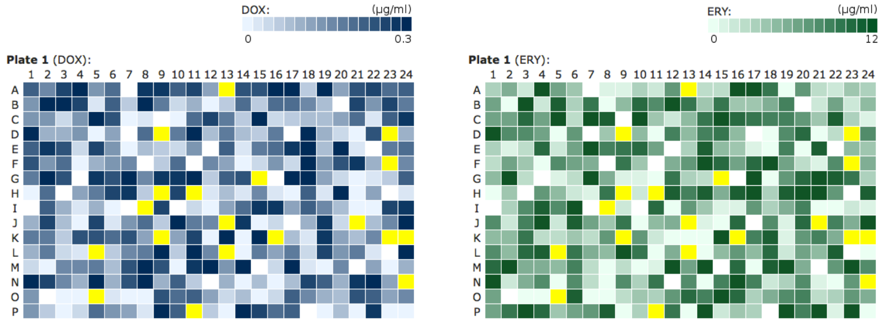

supplement fig. S3 – In order to exclude gradient effects, treatments across the 384-well plates were randomised using a pipetting robot (Tecan Freedom Evo). This figure illustrates typical randomisation patterns for different concentrations of (left) doxycycline and (right) erythromycin for 16 drug proportions in the equidosage line. White squares correspond to empty-well controls and yellow squares to null-antibiotic controls.

Basal concentrations of both drugs were chosen from the preliminary experiment to achieve 50% inhibition at 24h ($E_{50} = 9\mu g/ml$ for erythromycin and $D_{50} = 0.15\mu g/ml$ for doxycycline). This experiment then consisted of serial dilutions for each of the following sixteen drug combinations:

$$\left(\frac{0}{15}D_{50} + \frac{15}{15}E_{50}, \frac{1}{15}D_{50} + \frac{14}{15}E_{50}, ..., \frac{15}{15}D_{50} + \frac{0}{15}E_{50}\right).$$

Supplement fig. S4 illustrates the optical densities of a population of *E.coli* cultured at each of these drug combinations. In addition to these treatments, the following three controls were included: (1) M9 growth medium without antibiotics and without inoculation to serve as a reference for the density measurements, (2) M9 growth medium without antibiotics but with inoculation to serve as the uninhibited growth reference and (3) M9 growth medium with E_{50} **and** D_{50} to provide a lower-bound control on bacterial growth. All treatments and controls were replicated nineteen times and pipetted into one plate.

Each one of the five prepared microtiter plates was stored at 4°C until the day of their respective usage. So, in order to control for degradation of the drugs, a sixth 'checking' plate was prepared at the same time and stored with the other five plates throughout the duration of the five day experiment. At the end of the experiment this plate was inoculated with an overnight culture of the original colony and measured for 24h. Both on day one and when using the checking plate, doxycycline and erythromycin caused a significant reduction of the AUC (all 4 tests: Wilcoxon signed rank test, $W = 361, N = 19, p < 0.0001$). This is consistent with maintenance of the efficacy of the drugs for the entire five-day protocol.

An additional test was performed to determine whether resistance was the product of epigenetic adaptation alone. Ten samples each of the initially most synergistic drug treatment and the control treatment without drug were obtained from the end of the evolutionary experiment and cultured in M9 growth medium without antibiotics for 24h. Afterwards, the resulting populations were all subjected to the initially most synergistic drug combination for another 24h. Consistent with a likely genetic basis to drug-resistance adaptation, samples from the short-term synergistic treatment still displayed a higher AUC when compared to the no antibiotic control treatment (Wilcoxon signed rank test, $W = 92, N = 10, p < 0.001$).

(Ex. 3) **Checkerboards and Isobolograms: standard drug-resistance assays**.
Checkerboards were obtained by measuring the optical density at 24h of a grid composed of sixteen concentrations of each drug. Each one of the 256 drug combinations of our grid was replicated 11 times and pipetted onto 8 plates. Six wells with M9 growth medium but without inoculation were also included in a random distribution on each plate to serve as a contamination control and as density reference. Plates were kept in an incubator on shakers and density measurements taken every 45min for 24h.

The purpose of this experiment was to obtain two isobolograms: the first one was inoculated with the original isogenic population of susceptible bacteria while the second was inoculated with a sample from the highly synergistic combination ($4.8\mu g/ml$ erythromycin and $0.08\mu g/ml$ doxycycline) obtained at the beginning of the fifth day of Ex. 2.

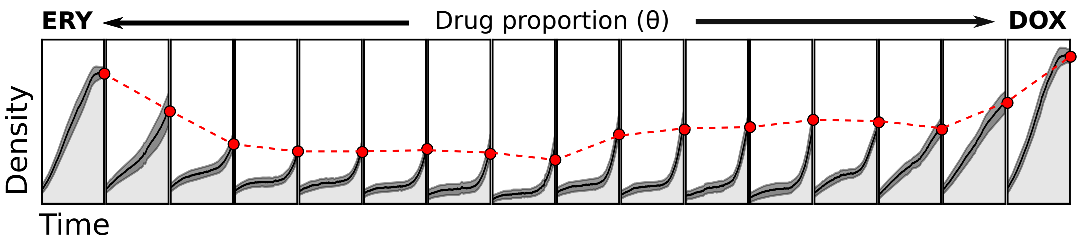

supplement fig. S4 – (**The U-shaped smile in data.**) Optical densities as a function of time for one 24-hour season under different drug treatments characterised by the parameter $0 \leq \theta \leq 1$. The box in the far left illustrates the optical density of bacteria growing in erythromycin monotherapy ($E = 9\,\mu g/ml$), the box at the right corresponds to doxycycline monotherapy ($D = 0.15\,\mu g/ml$), while every other box represents a multidrug combination treatment given by the pair $(\theta D, (1-\theta)E)$. The red dots indicated at the same time on each day show that bacterial density at that time is minimised when $\theta \approx 0.5$. As a result, we say that erythromycin and doxycycline have a synergistic interaction; note how this red curve has the characteristically near-convex 'U-shape' associated with synergy.

4. Experimental evolution in a two-drug environment

Remark 1. *In the following, the terms* 'day' *and* 'season' *will be used synonymously for 'transfer' in our batch-transfer protocol (*Ex. 2 *described above).*

4.1. **Trade-off between rate of adaptation and degree of synergy.** Now the serial dilution protocol 'Ex. 2' has been detailed, we can define a non-genetic measure of adaptation to treatment that uses the data it produces. Let N denote the number of transfers (ie. days) in a serial dilution experiment with each transfer denoted by the index $j \in \{1, N+1\}$. The density of bacteria at time t in a given well at transfer j is denoted by $B_j(t; D, E)$. If $0 < \eta \ll 1$ is a dilution parameter denoting the volume fraction of the dilution taken at the end of each day, then the act of performing this daily transfer can be described mathematical by the expression:

$$B_j(0; D, E) = \eta \cdot B_{j-1}(24; D, E). \tag{11}$$

A concept we make use of, closely related in spirit to one proposed in [5], is the *rate of adaptation* that we can now define. This quantity, illustrated in supplement fig. S5, measures how quickly resistance phenotypes spread within a population in a serial transfer experiment. Formally, the measure is defined as follows.

Definition 3 (Rate of adaptation)**.** *Denote by $Y_j(\theta)$ the total increase in bacterial density observed during transfer j:*

$$Y_j(\theta) = B_j(24; \theta D, (1-\theta)E) - B_j(0; \theta D, (1-\theta)E). \tag{12}$$

For an experiment of N transfers we obtain a series $\{Y_1, Y_2, ..., Y_{N+1}\}$ and so define the total yield improvement $\Delta Y(\theta) = Y_{N+1}(\theta) - Y_1(\theta)$. The time of adaptation*, t_{adapt}, is the interpolated time at which the bacterial yield improvement of the population reached half its maximum value. We say that the* rate of adaptation *of this bacterial population to this environment is*

$$\omega(\theta) = \frac{\Delta Y(\theta)}{2 \cdot t_{adapt}}. \tag{13}$$

For an illustration, see supplement fig. S5; also see [5] *for a related phenotypic measure of adaptation.*

Supplement fig. S6 shows the rate of adaptation as a function of the different degrees of synergy for each of the nineteen replicates of each combination treatment implemented in our experimental protocol. There are two important features of note: single-drug treatments (plotted as blue and green dots) are clustered in a region with low synergism, while multidrug combination treatments (plotted with red dots) present a high degree of synergism. Furthermore the figure shows a clear trade off: interactions with higher degrees of synergism possess higher rates of adaptation; this result is entirely analogous to one obtained earlier for different drug pairs [5].

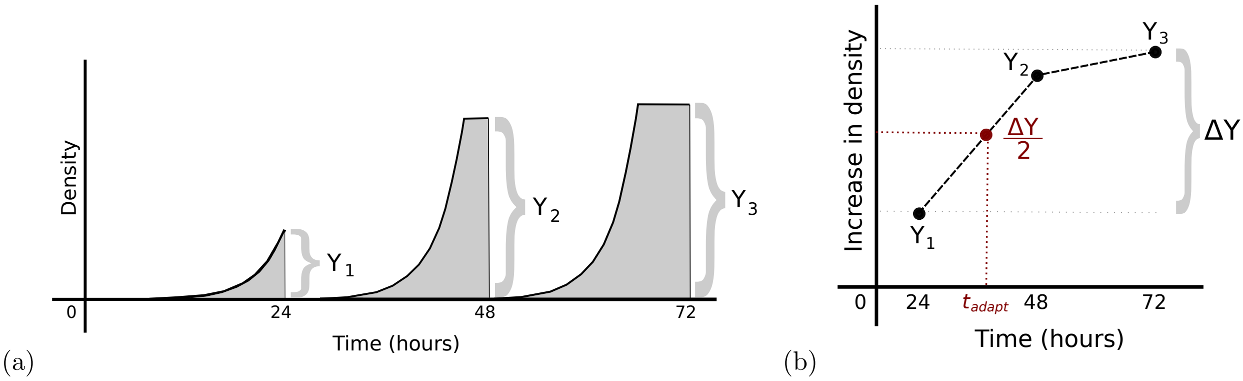

supplement fig. S5 – (a) An illustration of a schematic three-season experiment with an increase in bacterial yield at the end of each transfer represented by the variable $Y_j(\theta)$. (b) The rate of adaptation is defined in terms of the yield improvement, $\Delta Y(\theta)$, and the time to achieve half its maximum difference, t_{adapt} as stated in (13).

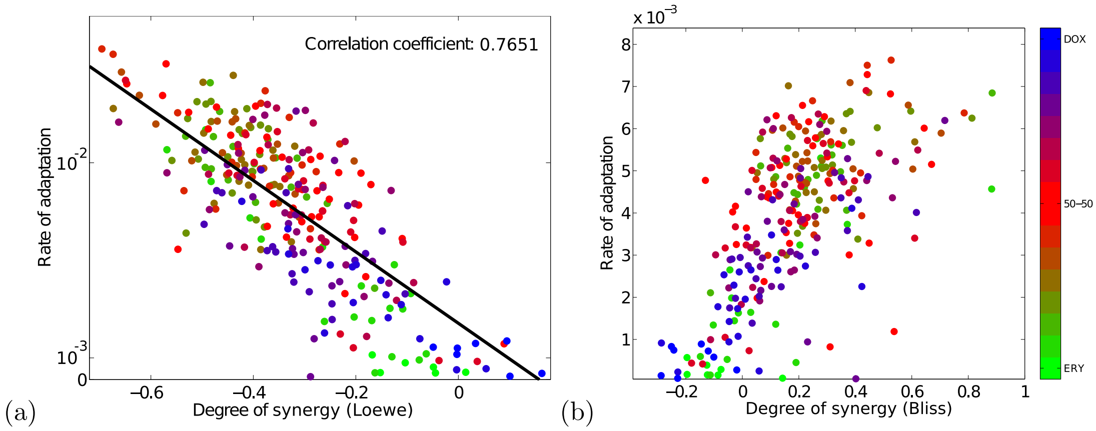

supplement fig. S6 – A correlation between rate of adaptation and degree of synergy determined using (a) Loewe additivity (Definition 1 with $r = 0.72$) and (b) Bliss independence (Definition 2). Independently of the definition of synergism used, multidrug combinations possess greater synergy and a higher rate of adaptation than treatments biased towards one drug with their necessarily lower degree of synergy (red dots represent near 50-50 combinations, blue dots represent doxycycline monotherapy and green dots erythromycin monotherapy).

4.2. **A measure of antibiotic efficacy: AUC inhibition.** The following measure of antibiotic efficacy takes into account the total bacterial density observed during a defined time interval in the presence and absence of drugs.

Definition 4 (AUC inhibition)**.** *Area under the curve inhibition at the end of day j is a measure of the fitness of bacteria relative to drug-free growth and expressed as a number between zero and one:*

$$\mathcal{A}(j; D, E) = 1 - \frac{\int_0^{24} B_j(t; D, E)dt}{\int_0^{24} B_j(t; 0, 0)dt}. \tag{14}$$

Hence $\mathcal{A}(j; D, E) = 0$ when drugs have no effect and $\mathcal{A}(j; D, E) = 1$ when the antibiotic combination has completely inhibited growth.

This measure is illustrated in supplement fig. S7.

4.3. **Smile-frown transition in the data.** The purpose of this section is to show that the smile-frown transition predicted by the logistic model (4) and depicted in supplement fig. S1 is found in the empirical dataset when *E.coli* K12(MC4100) is cultured in the presence of erythromycin and doxycycline using protocol Ex. 2.

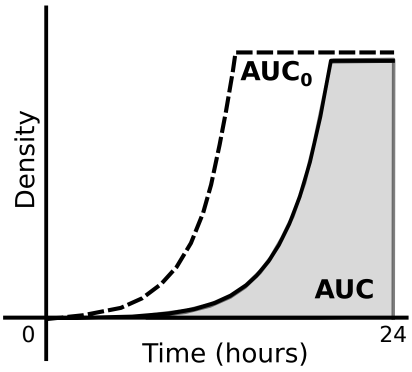

supplement fig. S7 – The area under the curve measure of inhibition ($AUC\ inhibition$) on day j is determined by integrating the total observed bacterial density throughout the duration of the season (represented with a grey area and denoted AUC) relative to the total observed bacterial density during the same time interval in an experiment with no antibiotics (area under the dotted line and denoted AUC_0). In symbols, inhibition is given by $\mathcal{A} = 1 - \mathrm{AUC}/\mathrm{AUC}_0$.

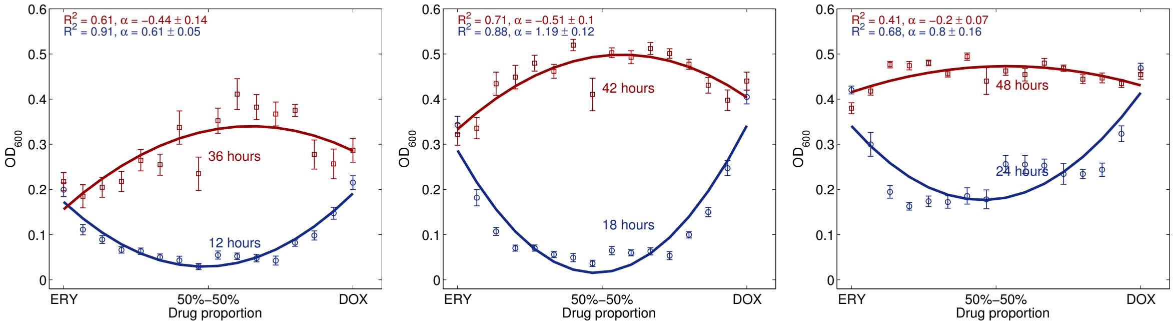

supplement fig. S8 – (**Loss of the synergistic smile**) Each pane shows a quadratic fit to optical density data at times 12h, 18h, 24h (in blue) and 24h later at 36h, 42h and 48h (in red). The earlier times are consistent with a test for synergism, the later are consistent with antagonism; the test and p values are given in the text in section 4.3. The figure legends show unadjusted R^2, the fitted value of α and its estimated standard error where the quadratic $\alpha\theta^2 + \beta\theta + \gamma$ is fitted to data; as usual, $\theta \in [0, 1]$ represents drug proportion on the x-axis.

The properties of synergy and antagonism as stated in Definition 1 are difficult concepts to apply directly to data as the variation inherent in any empirical observation of bacterial growth will mean that convexity or concavity of the population density data are unlikely to hold in practise. So, as to test for convexity or concavity in observed data, we fitted a quadratic $q(\theta) = \alpha\theta^2 + \beta\theta + \gamma$ to those densities for at least six different times during the first 48 hours of growth and examined the sign of the coefficient α (see supplement fig. S8). Motivated by the Definition 1 we take the convex case where $\alpha > 0$ as an indicator of synergy and the concave case where $\alpha < 0$ is an indicator of antagonism; a t-test is used for the significance of this sign condition.

Supplement fig. S8 illustrates the results obtained when applying this test. It shows that at 12h, 18h and 24h, the test is consistent with synergism with approximate p values for the positivity of α of $4.69 \cdot 10^{-8}$, $2.476 \cdot 10^{-7}$ and $2.475 \cdot 10^{-4}$ respectively. Twenty four hours later, at 36h, 42h and 48h the test is consistent with antagonism with respective p values $9.33 \cdot 10^{-3}$, $2.529 \cdot 10^{-4}$ and $1.25 \cdot 10^{-2}$. The R^2 values of each fit are given in the figure legend. Thus, we conclude that the smile-frown transition occurs in the data before 48h of growth have ellapsed, consistent with the model (4).

4.4. **A corollary of the smile-frown transition: when a monotherapy is optimal.** Equation (4) made a second prediction, not only is the smile-frown transition seen but it predicted that this transition is the result of the optimal therapy shifting from one timepoint to another. Moreover, at certain critical times the optimal therapy is predicted to move quite abruptly from being a combination therapy at early times to a monotherapy

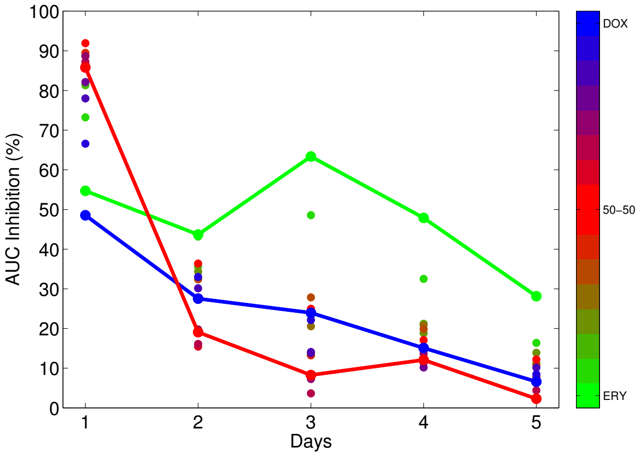

supplement fig. S9 — The decline in inhibitory effect for each of the sixteen treatments, where three have been highlighted: green is the erythromycin monotherapy, blue is the doxycycline monotherapy and red is the maximally synergistic combination (as measured over 24h). A point arises around day two where the loss of inhibition is so rapid for the most synergistic treatment that it no longer provides the optimal therapy in this measure and a monotherapy is preferable.

at later times. This is illustrated in supplement fig. S1 and, as can be seen in supplement fig. S9, this prediction is also borne out by data.

Supplement fig. S9 shows that the inhibition of growth due to the most synergistic combination treatments rapidly declines, so much so that within a day the erythromycin monotherapy provides greater inhibition, so too the doxycycline monotherapy but the latter difference is less clear in the figure.

5. Analysis: whole genome sequencing

5.1. **Genetic adaptation in evolved populations of *E. coli* K12 (MC4100).** To identify the genetic mechanism that accounts for the smile-frown transition that occurred during the evolution experiments, we sequenced the genomes of ten evolved populations of each single drug treatment (the one where $E = 9\mu g/ml$ and the other where $D = 0.15\mu g/ml$) and of the combination treatment with the largest synergy effect on day one (where $E = 4.8\mu g/ml$ and $D = 0.07\mu g/ml$). To ensure the genetic changes we identify are correlated with adaptation to antibacterial treatment, we also sequenced ten control populations that evolved under experimental conditions but were not exposed to any antibiotics. Finally, we re-sequenced the ancestral population to mitigate against possible inaccuracies or changes between our starting strain and published *E. coli* MC4100 genomes.

5.2. **DNA extraction and sequencing.** To obtain DNA, subsamples of bacterial cultures of day 4 (protocol Ex. 2) were taken by scratching the surface of frozen material with a pipette tip. The tip was used to inoculate 20 ml of M9 growth medium containing the respective antibiotic concentrations these replicates had encountered in the serial dilution experiment. The cultures were grown for 24h at $30°C$, thus re-running day five of the experiment. Additionally, an LB overnight culture of the original agar plate colony (used for inoculation on the first day) was prepared for sequencing.

DNA was extracted using the DNeasy Blood & Tissue Kit (Qiagen, Hilden, Germany) according to the manufacturer's instructions for Gram-negative bacteria. The 41 samples were processed into genomic paired-end Illumina sequencing libraries according to standard methods [6]. Sequences were obtained for the ancestral culture and for all replicates of the control, doxycycline and combination treatments. We only obtained sequences for six replicates of the erythromycin single drug treatment.

5.3. **Data analysis.**

Error Correction and Quality Filtering. All reads were error corrected using Quake 0.3.0 [7] using a kmer size of 15. Adapter sequences (AGATCGGAAGAGCACACGTC and AGATCGGAAGAGCGTCGTGT) were stripped using SeqPrep (`https://github.com/jstjohn/SeqPrep`). Reads were then quality trimmed from both ends, to exclude any bases of Phred quality score lower than 26 using DynamicTrim.pl from the SolexaQA

package 1.10 [8]. To exclude short and orphan reads and to maintain pairing of reads between the two fastq files, reads shorter than 40 were filtered out using LengthSort.pl from the same package.

Mapping Onto Reference. Reads of all samples were mapped to the published *E. coli* K12 (MC4100) reference genome [9], which is available for download at `ftp://ftp.ncbi.nih.gov/genomes/Bacteria/Escherichia_coli_BW2952_uid59391/` (version NC_012759.1, accessed January 2012). The reference sequence was saved in the fasta format and indexed using the index subroutine of bwa 0.6.1 [10]. Subsequently, reads were aligned to the reference using the bwa sampe module for paired end reads. Reads were also mapped with GSNAP. A GMAP/GSNAP database with kmer size 15 was created and reads were mapped with GSNAP [11] with an expected paired-end length of 300, allowing 50bp deviation of the expected paired-end length. Using samtools [12], the sam files resulting from both mappers were sorted to genomic position and filtered to include only reads mapped in a proper pair and aligned with a mapping quality greater than or equal to twenty. The sorted bam files were indexed and basic statistics were calculated using the flagstat option in samtools. Per-base coverage was calculated using genomeCoverageBed in Bedtools [13]. Coverage was further analysed in R and Bioconductor using the HilbertVis and ShortRead packages.

Analysis of Structural Variation and SNPs. To call single nucleotide polymorphisms (SNPs), duplicate reads were removed using the MarkDuplicates code of Picardtools (available at `www.picard.sourceforge.net`). SNPs were called with SNVer 0.4.0 [14] and with VarScan 2.0 [15]. SNVer uses a frequentist approach to test whether a polymorphic genomic locus represents a rare variant present above a minor allele frequency (MAF) treshhold. In our analysis the MAF was set to 0.05 and SNPs were only kept if they were supported by more than two reads. VarScan uses a heuristic/statistic method to call SNPs based on read depth, base quality, significance and variant frequency. In this analysis all detected variants with a minimal coverage of 50 were kept. The filtered SNPs were finally annotated using the published annotation file for *E. coli* K12 (MC4100) (`ftp://ftp.ncbi.nlm.nih.gov/genomes/Bacteria/Escherichia_coli_BW2952_uid59391/NC_012759.gff`) and a Perl script.

Structural variants were detected using Breakdancer [16], CNVnator [17] and Pindel [18]. Breakdancer detects structural variants based on anomalous location and/or orientation of read pairs. Using the breakdancer_max algorithm, all sorted bam alignment files were screened for indels, deletions, insertions, inversions and intrachromosomal rearrangements. Deletions, insertions and intrachromosomal rearrangements were kept if they received a score larger than 35 and were supported by at least 5 reads; for inversions, a quality score of 30 was considered sufficient. The Pindel pattern-growth algorithm detects breakpoints of large deletions and medium sized insertions by identifying paired reads for which only one of the reads can be mapped to the reference. It then attempts to break the unmapped read into two and map both shorter fragments to the reference. If successful, the breakpoints of deletions or insertions can thus be determined. CNVnator utilises statistical analyses of read mapping density (i.e., coverage) within different bins along the genome to discover copy number variants of any size. After calibration, a bin size of 100 was chosen for all CNVnator analyses.

5.4. **Summary of results.** All SNP and structural variation analyses were first performed on the ancestor. This strain had 59 deletions (among which one large 49.2 Kb deletion) and 18 SNPs compared to the published reference (cited above). In the control treatment six SNPs, four deletions and one inversion were found in four genes. These likely represent adaptations to the growth conditions and were therefore filtered from the results for the other treatments. The doxycycline samples showed 11 SNPs and five 1-12bp deletions in five genes specific to the treatment (Table S1), whereas the analysis of the erythromycin replicates yielded three unique SNPs and 4 unique deletions in two genes (Table S1). In the combination treatment only one sample showed unique SNPs in two genes. Additionally to SNPs, the analysis detected a 315Kb duplication between genome positions 274201 and 589900 (supplement fig. S10). This duplication was found in 90% of all combination replicates, but also in 30% of the doxycycline and 30% of the erythromycin treatments.

Genetic basis of adaptation. The above genomic analyses have yielded evidence for genetic adaptation to drug treatment. Populations adapted differently to single drug treatments than combination treatment but generally followed the same evolutionary routes across independent biological replicates within a treatment.

In the doxycycline treatment, 60% of the replicate populations became resistant through SNPs (Table S1) and/or 1-14bp deletions in the *marR* negative regulator of the *marRAB* operon ([19, 20], for a review of *mar*, see [21]). The Multiple Antibiotic Resistance (*mar*) locus mediates resistance to tetracyclins, chloramphenicol,

Gene	Number of polymorphic sites	Frequency in replicates	Annotation
**Doxycycline**			
marR	7	0.5	Repressor of *marRAB* operon (latter involved in activation of antibiotic resistance and oxidative stress genes)
mdaB	1	0.1	NADPH quinone reductase
agaS	1	0.1	tagatose-6-phosphate ketose / aldose isomerase
ascF	1	0.1	phosphotransferase system IIC components (carbohydrate transport)
eco	1	0.1	serine protease inhibitor
**Erythromycin**			
acrR	1	0.2	*acrRAB* regulator (antibiotic transporter operon)
ycbZ	2	0.6	ATP-dependent protease posttranslational modificatiom
**Combination**			
rcnA	1	0.1	membrane protein conferring nickel and cobalt resistance
evgS	1	0.1	hybrid sensory histidine kinase in two-component regulatory system

Table S 1 – Overview of single nucleotide polymorphisms in the genomes of *E. coli* that evolved five days with erythromycin, doxycycline or a combination of both. The number of polymorphic sites indicates how many independent nucleotide positions in the gene carry a SNP in at least one replicate. The frequency reflects the number of replicates where a polymorphism in the gene was found. The table only shows SNPs unique to the three treatments.

penicillins, cephalosporins, nalidixic acid and rifampicin through an energy-dependent efflux system [22]. Specifically, MarA transcriptionally activates at least 60 genes [23, 24, 25] that affect outer membrane permeability, antibiotic efflux and the reducing potential of the cell. Mutations that render the negative regulator MarR inactive have been found scattered across the gene and resulted in an increased efflux of antibiotics (see the overview in [26] and references therein). This *mar*-related mechanism is well-described as a genetic adaptation conferring resistance to tetracyclins.

In the erythromycin treatment, most replicates had a SNP or deletion in the *ycbZ* gene involved in translational modification (*ycbZ* is a putative protease). One replicate population obtained a mutation in *acrR*, the negative regulator of the *acrAB* multidrug efflux system. The *acrAB* locus is known to be one of the most important broad substrate efflux pumps in *E. coli* [27, 20, 28]. Deletion of this operon leads to hypersensitivity to antibiotics, detergents and dyes [29, 20], indeed Mazzariol and coworkers [30] showed that high-level resistant clinical isolates of *E. coli* over-expressed *acrA*.

In 90% of the populations in the combination treatment, but also in 30% of the erythromycin and 30% of the doxycycline treatments, a 315 Kb duplication was found (see supplement fig. S10). The duplication was observed significantly more for the combination treatment than in the erythromycin (Fisher's exact test, P=0.035) and the doxycycline (Fisher's exact test, P=0.019) treatments. All 14 replicates where a duplication was detected were consistently between positions 274201 and 589900. This genomic region contains 293 genes, among which eleven known antibiotic resistance genes, 32 transporter genes and 31 transposon-related genes (Appendix B).

Three multidrug efflux systems and an antibiotic degradation system are encoded in the duplicated region (again, see Appendix B). Nicoloff *et al.* showed [31] that a multi-drug resistant isolate of *E. coli* gained antibiotic resistance through a 149 Kb duplication of a region including the *acrAB* locus. Artificial reduction of the copy number of *acrAB* lead to loss of the resistant phenotype but introduction of the duplicated region into a strain lacking the *acrAB* locus did not lead to sufficient AcrAB copy number to reach resistance. The role of spontaneous tandem duplications containing *acrAB* was later confirmed in several other *E. coli* strains [32].

The consistent parallel evolution towards a 315 Kb duplication here in all but one replicate of the combination treatments strongly suggests, therefore, that genetic amplification of multi-drug efflux pumps such as *acrAB* is the likely adaptation that confers the multi-drug resistance phenotype.

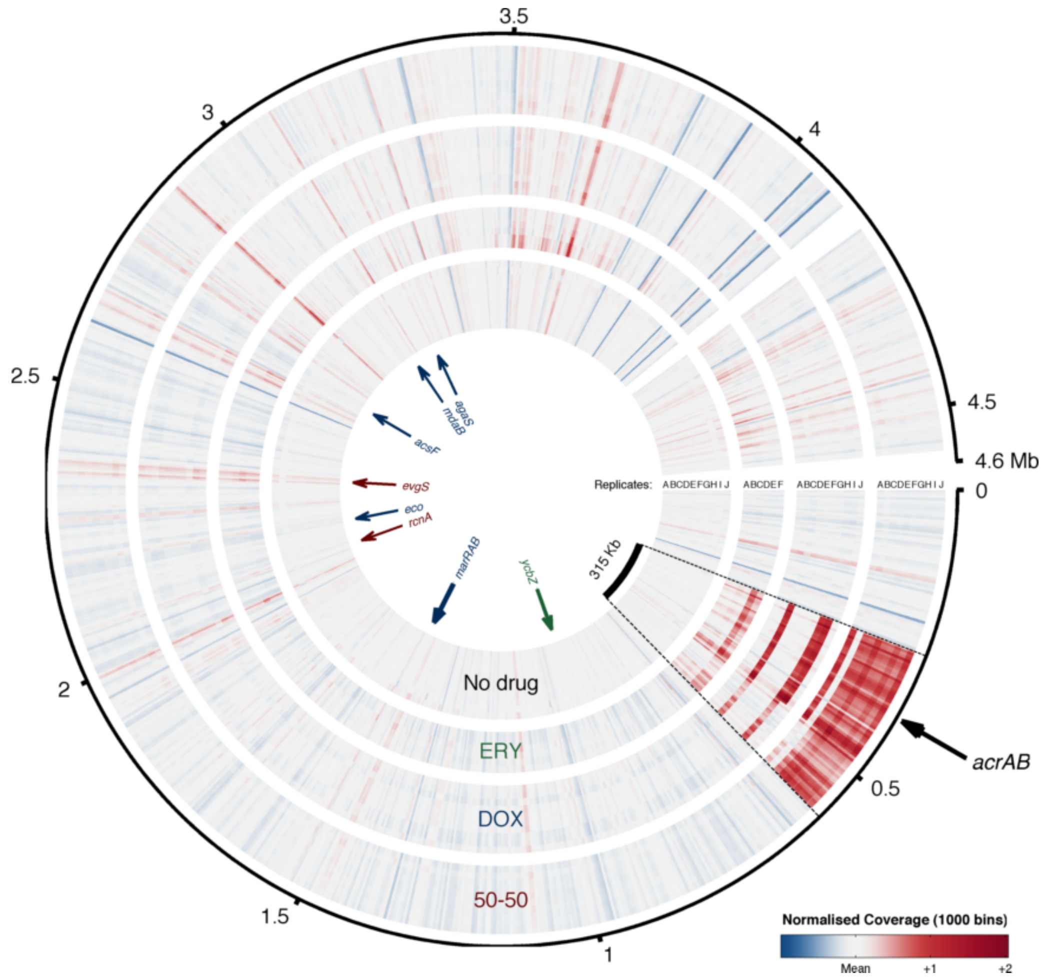

supplement fig. S10 – Coverage plots for the genomic region between positions 274,201bp and 589,900bp showing the duplication detected by CNVnator and breakdancer, an increase in coverage suggestive of a duplication of that region. It shows no duplications were detected in the absence of drugs but more duplications of the region were detected in replicates of the combination therapy than in monotherapies. These are indicated by the reddest regions. The arrows are colour-coded and highlight SNPs in different treatments, the thickness of the arrow denotes how many replicates that SNP was found in.

6. Analysis: a mathematical model consistent with data

The remainder of this document seeks to establish whether gene duplication of efflux genes underpins the genetic basis of the smile-frown transition. To this end, we now turn to mathematical models to ask whether the loss or gain of efflux genes in a model can produce the loss, or gain, of the transition.

6.1. **A core growth inhibition model: capturing dose-response curves.** Bacteriostatic antibiotics suppress bacterial growth by inhibiting DNA replication, RNA transcription or by interfering with protein synthesis and other aspects of cellular metabolism and physiology. For instance, both drugs in our experimental system target different subunits in the ribosome: *erythromycin* binds to the 50S ribosomal subunit and *doxycycline* to the 30S ribosomal subunit. Although they have different specific targets their mode of action is superficially similar, both suppressing cell growth by inhibiting protein translation.

The core growth-inhibition model presented in this section will use this observation to support the following modelling assumption: antibiotics act by inhibiting the production of an essential metabolite and therefore reduce flux through a pathway, thus reducing growth rate. While this is a vast over-simplification of the true cellular biology, this assumption will at least provide us with some broad insights into how two bacteriostatic antibiotics interact when they inhibit growth.

Cells were cultured in an environment in which glucose is limiting. We therefore assume for modelling purposes that the rate of cell growth is simultaneously proportional to the rate of translation and to the uptake

rate of glucose at extracellular concentration S. If we also assume that the antibiotic molecule has no effect on the uptake of the limiting resource, the growth rate G of a cell in which the extracellular concentration of a drug is D may then be written in the form $G(S, D) = \text{constant} \times u(S) \times \gamma(D)$. Here the constant represents cell yield per unit resource, glucose, $u(S)$ is the uptake rate of glucose and $\gamma(D)$ is a dimensionless growth inhibition function. We now assume that $u(S)$ is a standard Monod function,

$$u(S) = \frac{V_{\max} S}{K + S}, \tag{15}$$

where $V_{\max}$ is maximal resource uptake rate and K a half-saturation constant.

The decrease in growth rate due to the presence of antibiotic will be described by an inhibition function of the form

$$\gamma(D) = \frac{1}{1 + \kappa_d D}, \tag{16}$$

for some parameter $\kappa_d \geq 0$ that can is a phenotype of each cell. Note that κ_d is a single-cell measure of antibiotic efficacy in the sense that if D_{50} is the antibiotic concentration required to halve the translation rate then $D_{50} = 1/\kappa_d$. As a result, we assume that the per-cell, per-unit time growth rate of each bacterial type is determined both by the resource availability, S, and by the concentration of the antibiotic, D, and a resource conversion parameter, c, as follows:

$$G(S, D) = c \cdot u(S)\, \gamma(D). \tag{17}$$

Our experimental microcosm, the shaken 384-well plates described in section 3, can have a relatively high concentration of bacteria per unit of volume. However, as the plates are shaken continually and vigorously, we will assume that the bacteria diffuse in the liquid medium in such a way that it is possible to describe antibiotic and carbon uptake using models based on the mass action law, as described by the following system of differential equations:

$$\frac{d}{dt}B \;=\; G(S, D) \cdot B,\; \frac{d}{dt}S \;=\; -u(S) \cdot B,\; \frac{d}{dt}D \;=\; -d\,D \cdot B, \tag{18}$$

suitably augmented with initial conditions $(B(0), S(0), D(0))$. Here B represents the density of bacteria in the growth medium and d represents the rate of uptake of antibiotic molecules from the environment due to their binding to bacterial cells.

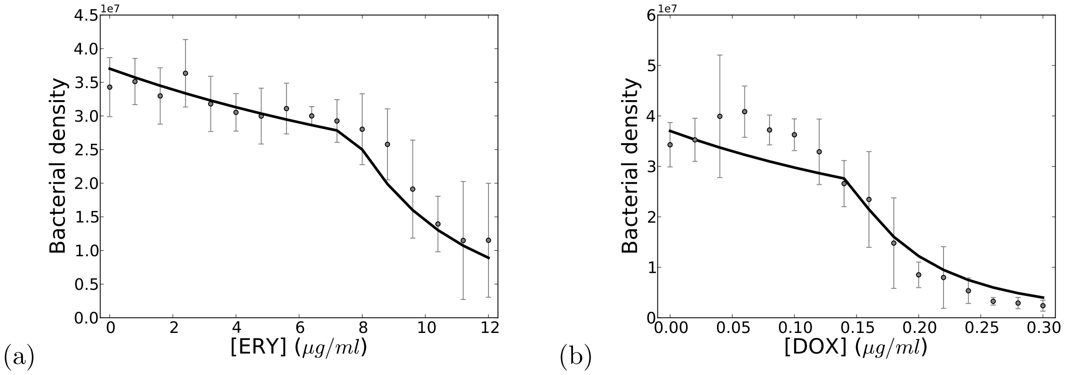

supplement fig. S11 – Dose-response curves of *E. coli* K12(MC4100) exposed to different concentrations of (a) erythromycin and (b) doxycycline. Circles represent data and lines are model simulations (ie. equations (18) with parameters as described in Appendix A; vertical lines are s.e.).

Many simplifying assumptions were used to formulate equation (18). So, in order to validate this core growth-inhibition model, we performed numerical simulations with parameter values described in Table S3 and compared the model with data obtained by culturing *E. coli* for 24h under glucose limitation and subject to increasing concentrations of erythromycin and doxycycline. As can be seen in supplement fig. S11, the model was able to capture quantitative features of the dose-response curve to both drugs. Following the experiments, the model used a glucose value of $S(0) = 2000\mu g/ml$ and the value of $D(0)$ can be read from the x-axis of supplement fig. S11 where 'D' in this model represents both drugs, doxycycline and erythromycin, in either case.

6.2. **Modelling synergistic drug interactions.** Having successfully captured single-drug dose-response profiles using a core mathematical model, we now return to the situation where two antibiotics are present in the environment at concentrations D and E. A natural extension of the model presented in the previous section may be obtained by supposing that growth inhibition can be described by a dimensionless function that depends on the concentrations of both drugs, $\gamma(D,E)$.

In a general sense, $\gamma(D,E)$ must be a function satisfying

$$\gamma(0,0)=1, \gamma(D,E)\geq 0, \gamma(D,0)=\gamma_d(D) \qquad \text{and} \qquad \gamma(0,E)=\gamma_e(E),$$

where $\gamma_d(D)$ and $\gamma_e(E)$ are growth inhibition functions that characterise the use of each antibiotic separately. Motivated by the action of doxycycline and erythromycin, we assume that the drugs have non-overlapping targets on a single enzyme and that we can model their interaction as a simplification of a model of mutually non-exclusive competitive inhibitors. As a result, the combined effect of the drugs D and E are assumed to have the following functional form

$$\gamma(D,E)=\frac{1}{1+\kappa_d D+\kappa_e E+\kappa_m DE}, \tag{19}$$

where κ_d, κ_e and κ_m are constant and strictly positive.

To see that the function $\gamma(D,E)$ so-defined yields a *synergistic* interaction in the sense defined in inequality (6), let θ lie between 0 and 1 and suppose that D_0 and E_0 have been chosen so that $\gamma(D_0,0)=\gamma(0,E_0)$. From this we deduce that $\kappa_d D_0=\kappa_e E_0$ must hold. Differentiating $\gamma(\theta D_0,(1-\theta)E_0)$ with respect to θ gives

$$\begin{aligned}\frac{\partial}{\partial\theta}\gamma(\theta D_0,(1-\theta)E_0) &= -\left(1/\gamma(\theta D_0,(1-\theta)E_0)\right)^2\cdot\left(\kappa_d D_0-\kappa_e E_0+(1-2\theta)\kappa_m D_0 E_0\right)\\ &= -\left(1-2\theta\right)\left(1/\gamma(\theta D_0,(1-\theta)E_0)\right)^2\kappa_m D_0 E_0\end{aligned}$$

which changes from negative to positive values when passing through $\theta=1/2$. Hence the function $\gamma(\theta D_0,(1-\theta)E_0)$ is monotonic decreasing for θ in $(0,1/2)$ and monotonic increasing in $(1/2,1)$ with maxima at both boundaries, $\theta=0$ and $\theta=1$. Furthermore, a short calculation shows that

$$\frac{\partial^2}{\partial\theta^2}\gamma(\theta E_0,(1-\theta)E_0)=2\kappa_m D_0 E_0\gamma(\theta D_0,(1-\theta)E_0)^{-2}\left((1-2\theta)\cdot\gamma^{-1}\cdot\frac{\partial}{\partial\theta}\gamma+1\right).$$

As $(1-2\theta)\cdot\frac{\partial}{\partial\theta}\gamma$ is positive, we deduce that $\frac{\partial^2}{\partial\theta^2}\gamma>0$ and so $\gamma(\theta D_0,(1-\theta)E_0)$ is convex as a function of θ. The function $\gamma(\theta D_0,(1-\theta)E_0)$ therefore satisfies the synergy property required for inhibition functions set out in Definition 1 (ie. the required convexity property holds), justifying our claim of synergy for γ.

We can now give a mathematical form for bacterial growth rate, G, in terms of the concentration of available limiting resource and the concentration of both antibiotics in the environment. This is a standard Michaelis-Menten term multiplied by the growth inhibition coefficient defined in (19):

$$G(S,D,E)=c\cdot u(S)\cdot\gamma(D,E). \tag{20}$$

As for one-drug models presented earlier, c denotes a resource conversion rate and $u(S)$ is the resource uptake function defined in (15). If we now write $B(t)$ for the density of an isogenic population of bacteria growing under resource limitation and under the effect of the antibiotics, at concentrations $D(t)$ and $E(t)$, then the equation governing the dynamics the bacterial density can be written thus:

$$\frac{d}{dt}B = G(S,D,E)\cdot B,\ \frac{d}{dt}S = -u(S)\cdot B,\ \frac{d}{dt}D = -d\,D\cdot B,\ \frac{d}{dt}E = -e\,E\cdot B, \tag{21}$$

with initial conditions $\boldsymbol{x}(0)=(B(0),S(0),D(0),E(0))$. Here the affinity constants, d and e, are mass action constants that represent the binding and uptake rates of the antibiotic molecules to the bacterial cells.

Using the parameter values described in Table S3, the model defined by (21) is seen in supplement fig. S12 to be consistent with an empirically-determined bacterial inhibition dataset obtained by culturing the bacteria at different drug proportions for 24-hours. This figure establishes that monotherapies produce an AUC measure of 50%, whereas a 50-50 combination therapy of both drugs produces a reduction in AUC growth of nearly 90%. This also establishes that the interaction of the drugs is synergistic when measured over a twenty-four hour period.

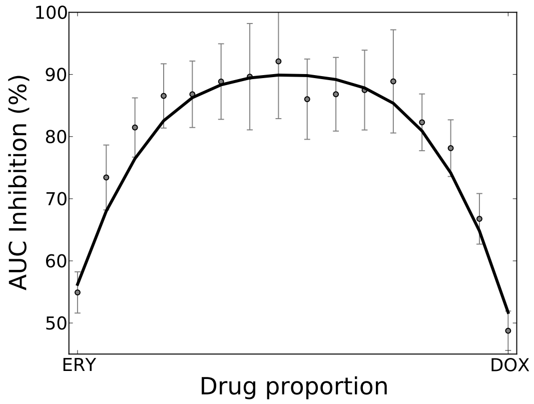

supplement fig. S12 – Area under the curve inhibition of *E. coli* K12 (MC4100) cultured for 24h under glucose limitation and different proportions of erythromycin and doxycycline. Solid lines are simulations of the mathematical model (21) with parameter values defined in Table S3, vertical bars are one s.e..

6.3. **A mathematical model structure to mimic the experimental protocol.** Suppose that $\boldsymbol{B}(t) = (B^1(t), \ldots, B^n(t))$ is a vector containing the density of n bacterial genotypes supported by the model at time t; the meaning of the different bacterial genotypes will be defined carefully in due course. The first entry of this vector represents the density of a wild-type bacterium in the experimental device and as the initial stage of the experiment is to inoculate the first flask with an isogenic population consisting only of wild-type bacteria at density B_0, we assume that $\boldsymbol{B}(0) = (B_0, 0, ...0)$. The value B_0 is approximately $10^4 - 10^5$ cells per ml in practise.

If the experimental protocol invokes N flask-to-flask transfers, each represented by an index $j \in \{1, N+1\}$, then the density of each bacterial genotype at transfer j is determined by the terminal densities from the previous day. If the duration of each transfer is T hours and in practise $T = 24h$, then the density of each bacterial genotype within the j-th day will be written $\boldsymbol{B}_j(t)$ for $0 \leq t \leq T$. As a consequence of the nature of the experimental protocol, in particular (11), the initial condition within each transfer will satisfy the condition $\boldsymbol{B}_j(0) = \eta \cdot \boldsymbol{B}_{j-1}(T)$, where $0 < \eta < 1$ is a dilution parameter.

Let us represent the state of the experimental system by the variable $\boldsymbol{x}_j(t) = (\boldsymbol{B}_j(t), S(t), D(t), E(t))$, where D and E are the concentrations of erythromycin and doxycycline and S is the limiting resource, glucose. A general, mathematical structure representing a population of *E.coli* competing in a single resource-limited environment and subject to the inhibiting effect of two bacteriostatic antibiotics can be written as follows:

$$\frac{d}{dt}\boldsymbol{x}_j = \mathcal{F}(\boldsymbol{x}_j) \tag{22a}$$

(where $\mathcal{F}$ is a model-specific mapping detailed below) with initial conditions

$$\boldsymbol{x}_j(0) = (\eta \cdot \boldsymbol{B}_{j-1}(T), S_0, \theta D_0, (1-\theta)E_0) \tag{22b}$$

within each season where $\boldsymbol{x}_1(0) = (\boldsymbol{B}(0), S_0, \theta D_0, (1-\theta)E_0)$. Here D_0 and E_0 are basal concentration of the two antibiotics and S_0 is the initial concentration of the limiting resource within each season. There remains to specify the nature of the function $\mathcal{F}$.

Remark 2. *The time series $\boldsymbol{x}_j(t)$ depend implicitly on θ through the definition of the initial condition within each transfer (although $\mathcal{F}$ does not itself depend on θ directly). To emphasise this dependence later in the article, we will use the notation $\boldsymbol{x}_j(t;\theta)$.*

6.4. **A drug efflux model.** To complete the construction of a the model, we assume that a protein is synthesised that interacts physically with the drug. This protein could in principle have several functions, the one we assume here is a passive efflux mechanism whereby the small antibiotic molecule is transported into the extracellular environment by one or more of these proteins. We could, with a small number of modifications, assume the protein is instead an enzyme that hydrolyses the antibiotic into a number of products that are harmless to the cell. Howver, we choose drug efflux for definiteness motivated by the fact that *acrAB* was found within the duplicated chromosomal region as highlighted by the above genomic analysis.

That part of our mathematical model which represents the dynamics within one season is defined as follows (this defines the function $\mathcal{F}$ above):

$$\frac{d}{dt}b_1 = G(S, D_1, E_1)b_1 - \delta(b_1 - (1+\Delta)b_2), \tag{23a}$$

$$\frac{d}{dt}b_j = G(S, D_j, E_j)b_j - \delta((2+\Delta)b_j - b_{j-1} - (1+\Delta)b_{j+1}), \tag{23b}$$

$$\frac{d}{dt}b_n = G(S, D_n, E_n)b_n - \delta((1+\Delta)b_n - b_{n-1}), \tag{23c}$$

$$\frac{d}{dt}S = -\frac{VS}{K+S}\sum_{j=1}^{n} b_j, \tag{23d}$$

$$\frac{d}{dt}D_{\text{ext}} = -d_D D_{\text{ext}} - \sum_{j=1}^{n} b_j \left(\varphi_d(D_{\text{ext}} - D_j) - \frac{v_d p_j}{k_d + p_j} D_j\right), \tag{23e}$$

$$\frac{d}{dt}D_j = -d_D D_j + b_j \left(\varphi_d(D_{\text{ext}} - D_j) - \frac{v_d p_j}{k_d + p_j} D_j\right), \tag{23f}$$

$$\frac{d}{dt}E_{\text{ext}} = -d_E E_{\text{ext}} - \sum_{j=1}^{n} b_j \left(\varphi_e(E_{\text{ext}} - E_j) - \frac{v_e p_j}{k_e + p_j} E_j\right), \tag{23g}$$

$$\frac{d}{dt}E_j = -d_E E_j + b_j \left(\varphi_e(E_{\text{ext}} - E_j) - \frac{v_e p_j}{k_e + p_j} E_j\right), \tag{23h}$$

where $j = 2, ..., n-1$.

The model in (23) is designed to capture the densities of bacteria that carry j copies of a gene coding a protein that transports drug from the cell, we denote this density by b_j. Using t for time, the variable S is the concentration of a limiting carbon source, glucose, D_{ext} and E_{ext} are extracellular concentrations of each drug and D_j and E_j are the intracellular drug concentrations for each subpopulation. The drugs are assumed to degrade at an exponential rate determined by d_D and d_E. The variable p_j encodes the number of efflux proteins expressed from a cell with $j-1$ copies of the efflux gene, provided $j \geq 2$, we then impose the conditions $p_1 = 0$ and $p_2 > 0$. This means that cells with one gene copy must first express it before the drugs can be pumped from the cell, thereafter that gene may be amplified.

We assume a functional form for p_j that is monotone increasing and bounded in j, controlled by a dimensionless constant g (the Michaelis-Menten function $p_j = (j-1)/(1+g(j-1))$). Thus p_j is also dimensionless and the quantity $p_j/(k_e + p_j)$ is the probability of finding a pump in the state where it momentarily is bound to drug. The rationale for this is that the polymerase transcription complex, assumed limited in number, has to compete for each gene copy, thus providing a limit on the number of efflux genes that can be simultaneously expressed. Thus the cell phenotype for which $j = 1$ has the gene for drug efflux, but does not express it.

The process of up-regulation of efflux genes and both increases and decreases in the copy number of the efflux genes are assumed to occur randomly as a Poisson process at a certain rate δ per cell per hour (the probability of expression and amplification of the gene per cell per unit time are assumed, for simplicity, to be the same).

The remaining variables in (23) have the following meaning: φ_e, φ_d are antibiotic diffusion rates across the cell membrane, v_e, v_d are maximal antibiotic efflux rates and k_e, k_d are half-saturation constants associated with efflux pump-antibiotic molecule binding; V and K are maximal uptake rate and half-saturation constants associated with Michaelis-Menten uptake of the single carbon source, glucose S; $G(S, D, E)$ is the per hour growth rate of each cell detailed above; δ is the rate of amplification of the efflux gene and $\delta(1+\Delta)$, a value necessarily greater than δ, is the rate of decay of the efflux protein expressed by this gene. Finally, therefore, $n-1$ is the maximum copy number of the gene.

To complete the statement of the model, we set $n = 3$ to represent three different cell phenotypes: an unexpressed pump gene, a single expressed pump gene and one additional copy of that gene where both copies are fully expressed. (In supplement fig. S13 these cell types are referred to in the figure legend as 'no pump', 'pump' and 'gene copy', respectively.) Finally, at the end of each 24-hour season a sample of the current population is transferred to fresh medium where the next season of growth occurs. A sample of around 1% of the bacterial population was therefore taken and transferred to a fresh environment. The intracellular drug concentrations (per cell) were maintained in this transfer but the extracellular environment was reset to provide the growth and environmental dynamics seen in supplement fig. S13. This was repeated for five days (120h total growth) to respect the experimental protocol.

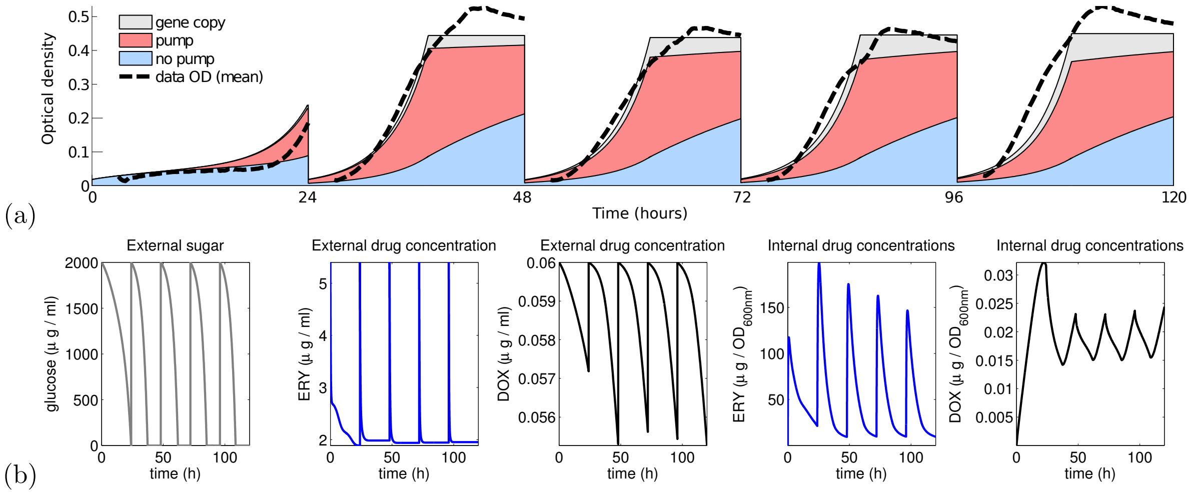

supplement fig. S13 – One simulation of the drug efflux model over five days (seasons of 24h) totalling 120h for a drug combination given by $\theta = 6/14$ corresponding to $D = 0.06\mu g/ml$ and $E = 5.4\mu g/ml$. (a) The densities of each phenotype (red, blue and grey areas) sum to form the total population density that is compared with mean observed optical density data (dotted black line). In simulations, the pump and its gene copy are selected for in the growth phase, but are selected against in stationary phase. (b) The drug and sugar concentrations sweep downwards from their highest values at the start of each new season where the bacterial densities and extracellular environments have been reset to their initial ($t = 0$) values.

Dynamics from the first two days' simulation of both the model and of the data have been sampled to produce supplement fig. S14. This figure shows that the drug efflux model and the data are quantitatively consistent and, according to the model, we do indeed find that the interaction profile between erythromycin and doxycycline has morphed from a synergistic to antagonistic form within a matter of hours.

For completeness we provide supplement fig. S15 which shows that while the transition from a synergistic to antagonistic interaction profile occurs on the second day of the experiment, thereafter the profile continues to be one of progressively weaker antagonism.

6.5. **Theoretical prediction: synergy is stabilised if the efflux pump is suppressed.** Each cell genotype in (23a-c) has the same absolute fitness, $G(S, D, E)$, with its synergistic dependence on D and E, each cell also has the same uptake rate of limiting resource. What differs between the cell types is the concentration of drug within each cell due to the number of pumps the cell produces. From our assumptions, it follows that there are no fitness costs of being drug-resistant in this model, the production of the efflux protein is assumed to carry no cost either in terms of growth rate or growth yield.

As the expression and amplification of the efflux pump genes can be prevented in (23) by setting $\delta = 0$, after effecting such a change it follows that (23) can only support one cell phenotype (under our assumptions) whose growth is described completely by the function $G(S, D_1, E_1)$. As this function is, by its very design, known to synergise with respect to the two drugs because $G(S, \theta D_1, (1-\theta)E_1)$ is convex as a function of θ, it follows that the drug interaction predicted by equation (23) must be synergistic for all times. We deduce that it is the superposition of different resistant sub-populations that eventually emerge if $\delta > 0$ that combine to produce the loss of synergy seen in data as illustrated in supplement fig. S14.

As a further illustration, we reproduced the bottom-left pane of supplement fig. S14 but after setting $\delta = 0$ (with all the other parameters as stated in Table S4). When we made this one change, the difference in the modelled drug interactions between twelve and thirty six hours can be seen in supplement fig. S16. Note in particular how the drug synergy persists to later times after effecting this change. Such an artificial suppression of the efflux pump, akin to a loss of function mutation in the pump gene, ensures that the designed drug synergy is a stable property of the model that leads to a continual decline in population size as the treatment proceeds.

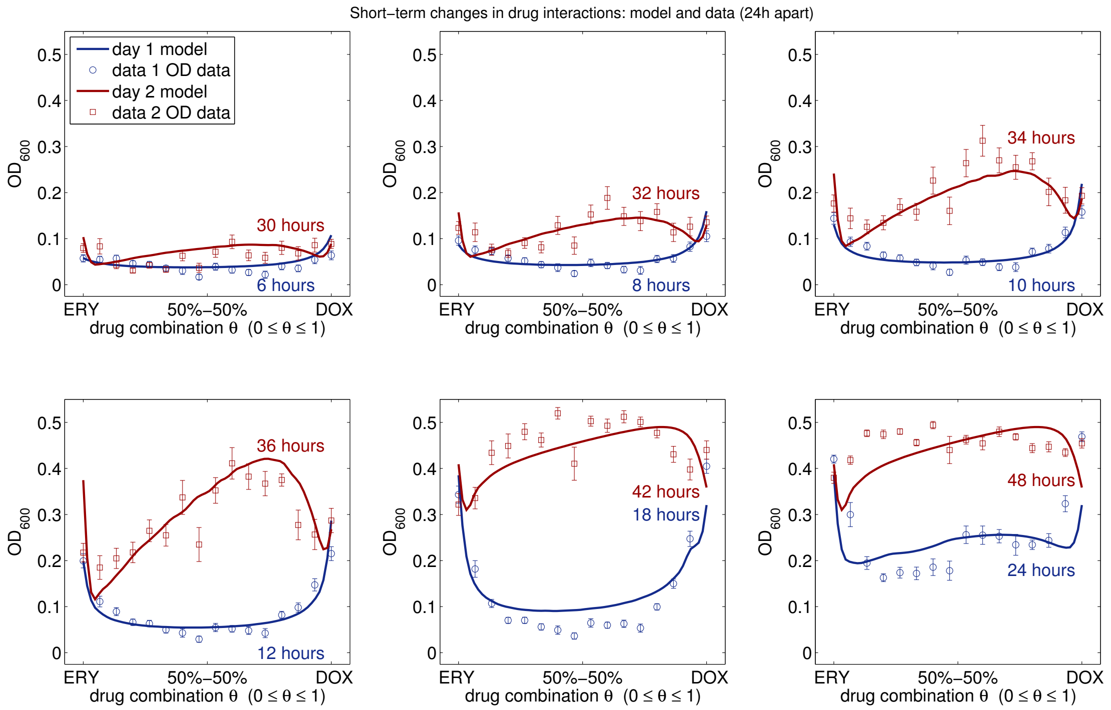

supplement fig. S14 — The transition from a synergistic to antagonistic drug interaction profile can be seen in optical density data illustrated here at different times 24h apart. The outcome of the model (unbroken lines) is superimposed upon the mean observed data (broken lines and squares; bars indicate s.e.). Until 18h the model indicates a convex interaction profile consistent with synergy (blue), beyond 30h the model combination therapies have a near-concave profile consistent with antagonism (red).

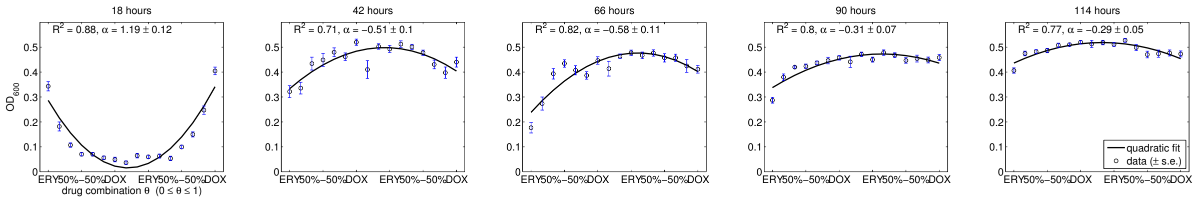

supplement fig. S15 — The smile-frown transition from day one to day two is proceeded by an antagonistic profile until the end of day five, with the strength of the antagonism decaying each day, as this figure shows (at hour 18 each day). The parameter value associated with the α test is provided in each figure window.

7. Validating the theory: testing the smile-frown experiment with an *acrAB* knockout

Our analysis of the mathematical model has thus provided a clear prediction: in the absence of key drug resistance genes, the 'smile' we associate with drug synergy will be stabilised as shown in supplement fig. S16. The purpose of this section is to test this.

7.1. **Additional experimental details.** Since a large duplication was found significantly more often for the combination rather than any of the other treatments, at least one of the duplicated genes may account for the smile-frown transition. One of the candidates is the *acrAB* operon which is responsible for expression of the *acrAB*-*tolC* multidrug efflux pump. We ran a second serial dilution experiment, namely Ex. 2, in which we compared the strain *E. coli* AG100 with the corresponding deletion mutant for the *acrAB* operon (*E. coli* (Δacr)). Otherwise, media and drugs were exactly as Ex. 2.

Having resolved a large duplication present in significantly more of the replicated treatments where a drug combination treatments was implemented when compared to the single drug treatments, the genomics indicated that the *acrAB*-*tolC* multidrug efflux system was likely to play a significant role in the smile-frown transition. We thus chose to treat this as a candidate mechanism and ran another serial dilution experiment, namely Ex. 2,

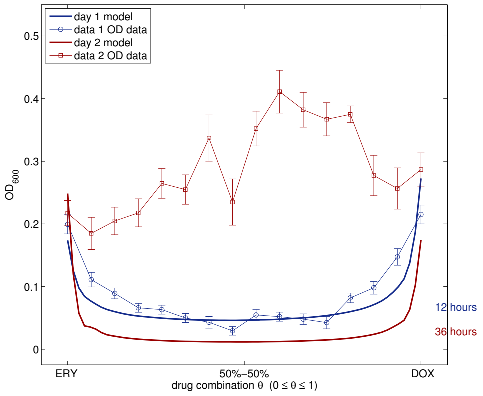

supplement fig. S16 – A theoretical model with just one drug-susceptible phenotype in which the efflux mechanism has been suppressed in the model (we set $\delta = 0$): drug synergy is maintained by this model at all times (shown here as unbroken, smooth lines at 12h and 36h). The observed population densities are also shown (with standard error bars) for comparison.

this time comparing *E. coli* K12 (AG100) [22] with *E. coli* K12 (AG100A)(Δacr) [28], where the latter is derived from the former by deletion of the *acrAB* operon. Otherwise, media and drugs were exactly as found in the previous serial dilution experiment described above, Ex. 2.

The same controls were also used. Calibration experiments (see Ex. 1) and the serial dilution experiment itself were inoculated with cultures prepared from the same colony on an agar plate, respectively. Deviating from Ex. 2, we now implemented five experimental concentration combinations for each of the two strains, these were:

$$\left(\tfrac{0}{4}D_{50} + \tfrac{4}{4}E_{50}, \tfrac{1}{4}D_{50} + \tfrac{3}{4}E_{50}, \tfrac{2}{4}D_{50} + \tfrac{2}{4}E_{50}, \tfrac{3}{4}D_{50} + \tfrac{1}{4}E_{50}, \tfrac{4}{4}D_{50} + \tfrac{0}{4}E_{50}\right).$$

For both strains and both drugs, IC_{50} values were calibrated as the concentrations giving 50% OD_{600nm} inhibition measured at 24h (see Table S2).

	E_{50} in $\mu g/ml$	D_{50} in $\mu g/ml$
AG100	7	0.16
AG100A	0.33	0.037

Table S2 – IC_{50} values determined for *E. coli* K12 strains AG100 and AG100A(Δacr).

For this serial dilution experiment, each treatment (including controls) was replicated 6 times and the transfers were implemented for three days using one 96 well plate per day (we combined both strains into one run on a single plate). Optical density (OD_{600nm}) was measured every 7.5 minutes, with a FLUOstar Omega plate reader controlling the temperature at 30°C and shaking the plate linearly at 300 rpm between measurements.

An additional microtiter plate was refrigerated alongside the other plates used throughout the duration of the serial dilution experiment. This was assayed with an inoculation culture from the original agar plate colony after the serial dilution experiment had finished to control for degradation of the drugs. No difference in growth between the first day of the experiment and the control experiment was found, we conclude that the drugs in the growth medium had not degraded during this part of the experiment.

7.2. **Outcome: synergy is maintained for longer with the pump knockout.** The results of this experiment are summarised in supplement fig. S17 and supplement fig. S18. These figures are completely consistent with what was anticipated from the predictions of the mathematical analysis: in the presence of the *acrAB-tolC* efflux system, the smile-frown transition is seen in strain AG100 but the AG100A(Δacr) it is lost. In particular, supplement fig. S18 shows that the loss of the *acrAB-tolC* pump ensures that erythromycin and doxycycline are still synergistic for the pump knockout strain AG100A(Δacr) even by day three of the protocol.

Supplement fig. S19 shows that synergy is lost by day four because the densities are now maximal in one of the combination treatments. However, the α-test does not show significant antagonism and so we cannot

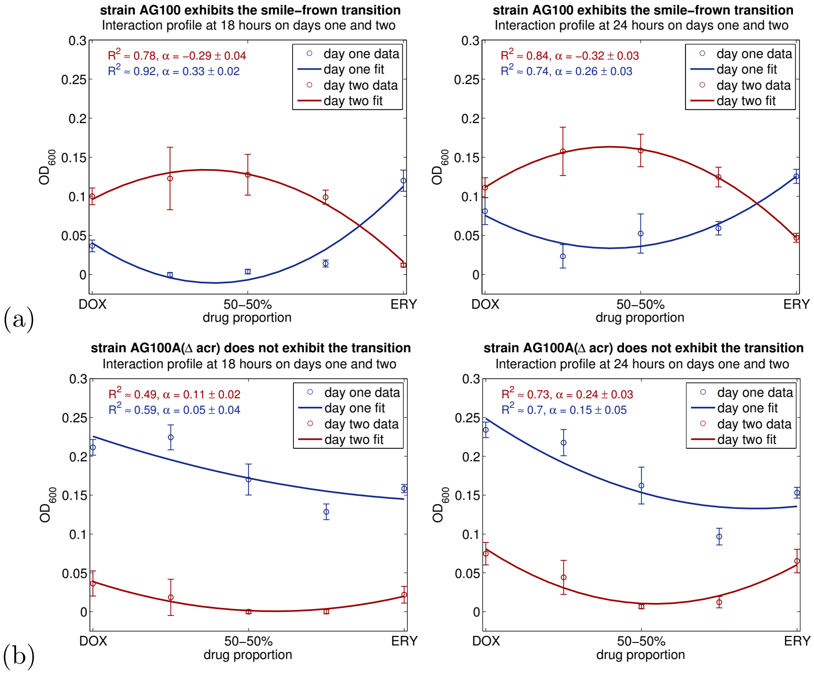

supplement fig. S17 – A repetition of the test for synergy applied in supplement figs. S8 based on a quadratic fit (and a t-test) to density data as a function of θ. Here (a) shows that strain AG100 undergoes the smile-frown transition using experimental protocol Ex. 2, reporting synergy on day one (red line quadratic fit, red circles data; t-test at 18h, $df = 27, t \approx 14.84, p < 10^{-13}$) and antagonism on day two (blue line quadratic fit, blue points data; t-test at 18h $df = 27, t \approx -7.45, p < 10^{-7}$). The data in (b) shows that the same experimental protocol provides no evidence for a change in drug interaction over 48h when the strain AG100A(Δacr) is used: the smile-frown transition does not occur (circles are data, vertical bars are one s.e., lines are a quadratic fit).

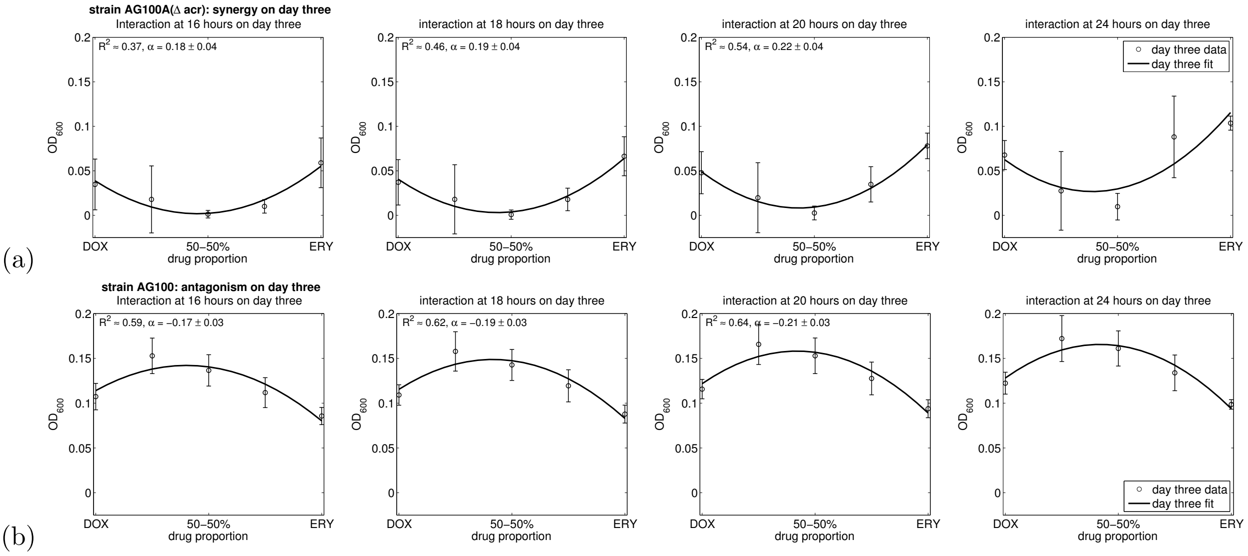

supplement fig. S18 – (a) The analogous experimental protocol and data to supplement fig. S17 for strain AG100A(Δacr) and now extended to day three. All four figures taken at four different times on this day are consistent with synergy ($p < \{0.0003, 10^{-4}, 10^{-5}, 10^{-6}, 0.0006\}$ for each figure separately (α in figure legend, $df = 27, t \approx \{4.17, 4.74, 5.45, 4.89\}$)) with no evidence of antagonism by the end of day three (rightmost figure). (b) The analogous figure to (a) above, but for the wild-type AG100 strain: antagonism is maintained on day three ($p < \{10^{-5}, 10^{-5}, 10^{-6}, 10^{-6}\}$ for each figure separately with $df = 27, t \approx \{-5.5, -6.12, -6.38, -6.38\}$).

claim that the smile-frown transition has occurred for this knockout strain, even though one of the combination therapies has now lead to the highest population density of all the treatments trialled.

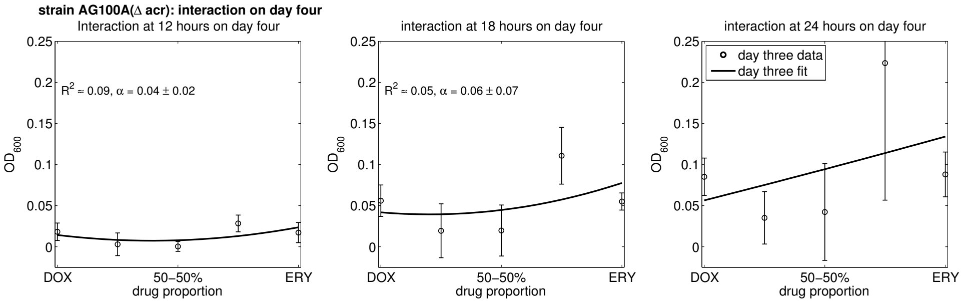

supplement fig. S19 − The α-test applied to day four data shows no statistically significant evidence of a synergistic or antagonistic interaction. However, the density of the population is maximised for a combination treatment, as can be seen in the middle pane at 18h.

7.3. **Dose-dependence: higher doses amplify the smile-frown transition.** In order to understand whether the smile-frown transition might be lost at different dosages or different strengths of synergy, we altered the model in equation (4) by redefining the interaction function $a(\theta)$ to be

$$a(\theta) = 1 + \lambda \cdot \theta(1 - \theta). \tag{24}$$

This coincides with the previous definition when $\lambda = 1$ and allows us to use λ as a parameter to alter the strength of synergy in this model. When we reproduce supplement fig. S1 for a ranges of λ values, we obtain supplement fig. S20 below. This indicates that the transition becomes more pronounced as the dose parameter λ increases.

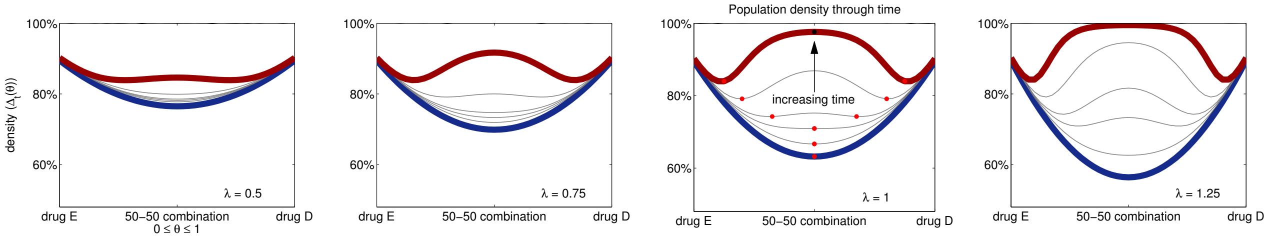

(a)

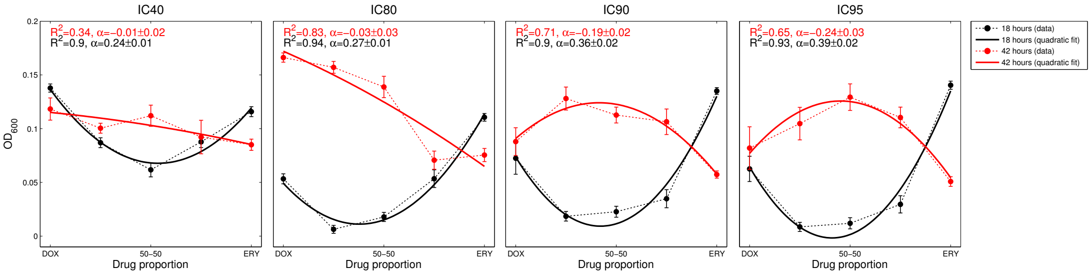

(b)

supplement fig. S20 − (a) When four different values of λ are used in the definition of a in (24) and this is implemented in the model defined by (4), the resulting computation indicates that larger values of this parameter produce a more pronounced smile-frown transition. (b) The analogy of this model prediction is observed in the experimental protocol executed at four dosages of increasingly inhibitory effect: IC40, IC80, IC90 and IC95 that are defined in the text. Each of these four datasets is consistent with the loss of synergy before the end of the second day of treatment.

In order to test this prediction we executed the evolutionary protocol at four different dosages using strain AG100, dosages that reduced growth relative to a zero-drug control by 40%, 80%, 90% and 95% (when measured at 18h on day one). As supplement fig. S21 shows, a correlation between dose, the strength of synergy and the strength of later antagonism can be seen in the empirical dataset. Indeed, when we plot the day one and

day two empirical drug interactions supplement fig. S21 shows a strongly negative correlation between the two. Moreover, supplement fig. S22 shows, in addition, that the smile-frown transition occurs earlier in the treatment as the dose is increased.

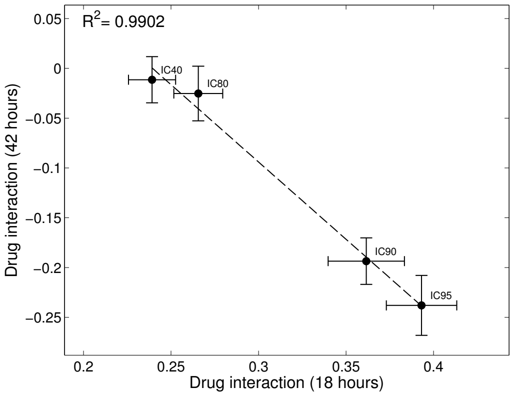

supplement fig. S21 − The experimental protocol executed at four dosages of increasingly inhibitory effect, IC40, IC80, IC90 and IC95, shows that the drug interaction on day one (18h) is negatively correlated with the interaction on day two (42h).

x

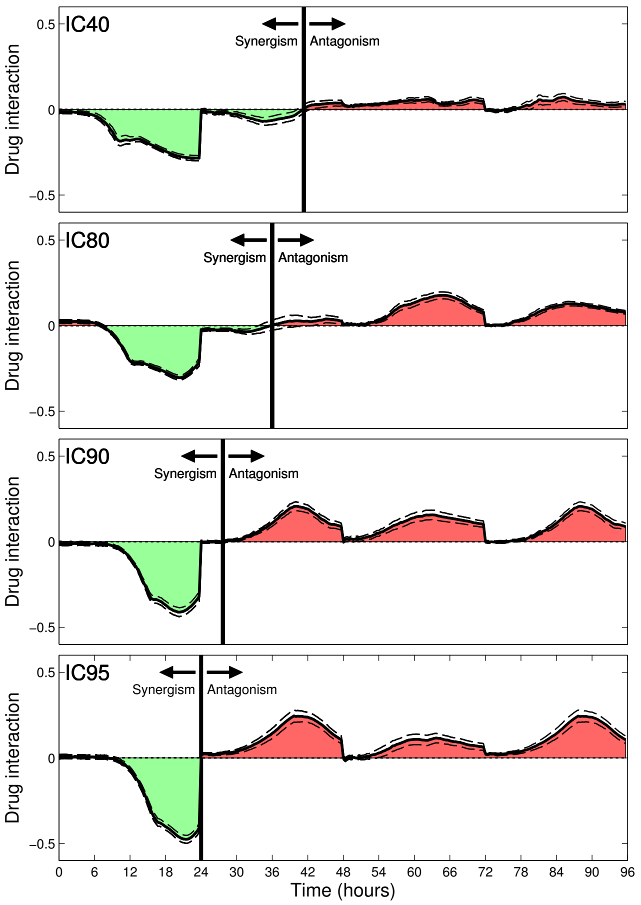

supplement fig. S22 − The value of $-\alpha$ from the α-test is shown on the y-axis (with s.e. plotted as dashed lines) and this indicates that the smile-frown transition occurs earlier as the antibiotic dose is increased.

7.4. **Colony forming units (CFUs) as a density measure.** In order to verify that optical density (at 600nm) also measures live cell densities we verified the existence of a linear correlation between OD_{600nm} and population density measured in CFUs. The resulting data is shown in supplement fig. S23(a) where the correlation is evident. As a result, when we sought to test the existence of a smile-frown transition in density units of CFUs, this was also observed within a period of two days, as can be seen in supplement fig. S23(b) which shows density in CFUs measured at 18h on day one and 42h on day two. The drug concentration was chosen so that a 50-50 combination therapy would achieve 95% growth inhibition when measured at 18h relative to a no-drug control culture.

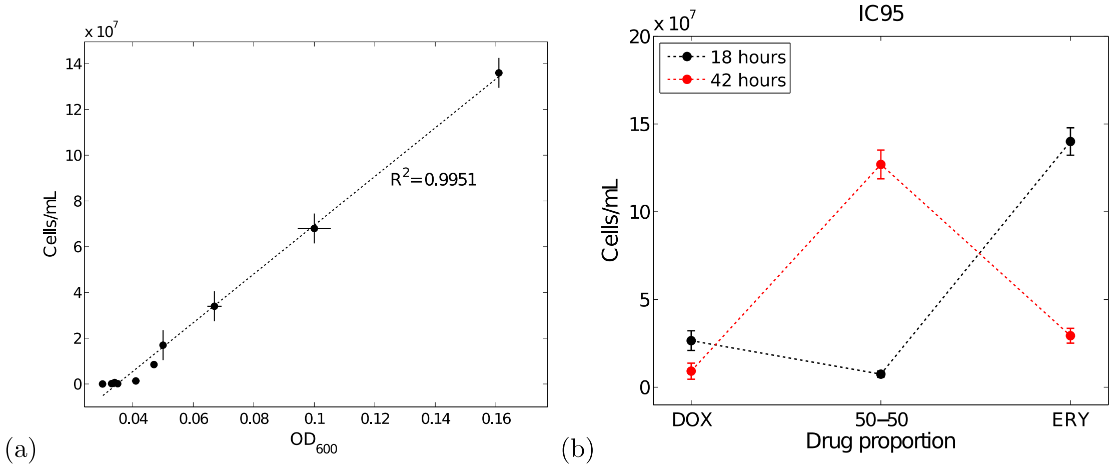

supplement fig. S23 – (a) Optical density (x-axis) is linearly correlated with population density as measured by colony forming units ('Cells/mL' on the y-axis). (b) As a result, we observe the existence of the smile-frown transition when measuring population densities in units of CFUs, here for three drug proportion parameters, $\theta = 0, 1/2$ and 1. In both cases we used basal drug concentrations so that 95% inhibition was achieved for the 50-50 combination treatment.

8. Optimal drug combinations are not constant: an analysis

Motivated by the result that both observed data and different mathematical models of drug deployment exhibit a change in optimal drug combination over time, we now seek mathematical results to understand the generality of this phenomenon. So, consider the following abstraction of a very large class of antibiotic-deployment models:

$$\frac{d}{dt}\boldsymbol{x} = T \cdot \mathcal{F}(\boldsymbol{x}, D, E) \tag{25}$$

where $\boldsymbol{x}(t)$, for $0 \le t \le 1$, is the state of the system encoding all the dynamical variables needed to described the dynamics of the system (we shall assume there is a finite number of such variables, call it n and so $\boldsymbol{x} \in \mathbb{R}^n$). This can be seen as a re-definition of equation (22a) but whereby the variable T that defines the length of each season in the experiment has been made explicit in (25) through a re-scaling of the time variable in (22a). We have also made basal concentrations of antibiotic explicit, D and E, for convenience below. Equation (25) is too general a mathematical problem with which to study drug combinations without imposing further structure, so we do that now.

The function $\mathcal{F}$ is assumed to be infinitely differentiable and we shall assume that there is a constant $\mathsf{C}(D, E)$ such that

$$\limsup_{T\to\infty} \sup_{0\le t\le 1} \|\boldsymbol{x}(t)\| \le \mathsf{C}(D, E), \tag{26}$$

where $\|\cdot\|$ denotes any finite-dimensional norm. Equation (26) is a dissipative condition that is commonly satisfied by physical systems for which a law of conservation of energy or biomass can be formulated. This bound states that solutions of (25) are eventually attracted to some ball that is independent of initial conditions but which may depend on other systemic parameters.

Suppose D is a constant that represents the extracellular input concentration of drug 'D' to the system and E is the analogous quantity for the other drug. Let us suppose without the loss of any generality that maximal concentrations of both drugs have been normalised to achieve equal effect, so that $0 \le D \le 1$ and $0 \le E \le 1$ and unity here represents the numerical value of that concentration.

As we have done throughout this manuscript, the general model class represented by equation (25) assumes that there are three dynamical systems that represent multiple drug use: $\frac{d}{dt}\boldsymbol{x} = T \cdot \mathcal{F}(\boldsymbol{x}, 0, 0)$ in the complete absence of drug use $\frac{d}{dt}\boldsymbol{x} = T \cdot \mathcal{F}(\boldsymbol{x}, D_0, 0)$ arises when we use D only and at concentration D_0, and $\frac{d}{dt}\boldsymbol{x} = T \cdot \mathcal{F}(\boldsymbol{x}, 0, E_0)$ is the analogous system but for drug 'E'. Given this formulation, we can now simulate the use of a fixed combination of both drugs with the equation

$$\frac{d}{dt}\boldsymbol{x} = T \cdot \mathcal{F}(\boldsymbol{x}, \theta D_0, (1-\theta)E_0). \tag{27}$$

The optimal drug combination is found by observing $\boldsymbol{x}(\cdot)$ over some period of time, T, and determining the drug combination that minimises this observation. Thus, we use the dimensionless parameter $\theta \in [0, 1]$ to represent the relative drug fraction and define the *drug interaction profile* at time T:

$$J(T, \theta) = \int_0^1 (\boldsymbol{w}, \boldsymbol{x}(t; \theta, T))dt \tag{28a}$$

where

$$\frac{d}{dt}\boldsymbol{x} = T \cdot \mathcal{F}(\boldsymbol{x}, \theta D_0, (1-\theta)E_0) \qquad \text{and} \qquad \boldsymbol{x}(0) = \boldsymbol{x}_0. \tag{28b}$$

We now seek the *optimal drug combination*, this is the number $\theta_{\text{opt}}(T)$ that satisfies

$$J(T, \theta_{\text{opt}}(T)) = \min\{J(T, \theta) : 0 \le \theta \le 1\}. \tag{28c}$$

The vector $\boldsymbol{w}$ that appears in the definition of J above is a weight vector whose entries sum to unity that attributes different weights to the components of $\boldsymbol{x}$.

The following restriction is not, in fact, used anywhere in the analysis below, but the idea that E and D represent growth-reducing drugs could be expressed, for example, by the abstract conditions that

$$\left(\boldsymbol{w}, \frac{\partial}{\partial D}\mathcal{F}(\boldsymbol{x}, D, E)\right) \le 0 \qquad \text{and} \qquad \left(\boldsymbol{w}, \frac{\partial}{\partial E}\mathcal{F}(\boldsymbol{x}, D, E)\right) \le 0$$

whenever $E \ge 0$ and $D \ge 0$. This pair of conditions means that growth rate is reduced in the components of $\boldsymbol{w}$ that contribute to the optimality condition whenever drug is increased in concentration.

8.1. **Mathematical definition of synergy.** Given this formulation, we now define exactly what we mean by synergy in mathematical terms.

Definition 5 (mathematical synergy)**.** *First define the 'interaction function'*

$$a(\theta) = (\boldsymbol{w}, \mathcal{F}(\boldsymbol{x}_0, \theta D_0, (1-\theta)E_0)$$

and then to ensure the basal drug concentrations, D_0 and E_0, are calibrated to have equal inhibitory effect, we assume that $a(0) = a(1)$. Strict drug synergy *is said to hold in (27) when a is* **convex***: $a''(\theta) > 0$ for all $\theta \in (0, 1)$.*

We define the most synergistic combination, $\theta_{syn} \in (0, 1)$, to be the value of θ for which the minimum of $a(\theta)$ occurs.

For completeness, we include the following definition.

Definition 6 (mathematical antagonism)**.** *Suppose that the basal drug concentrations D_0 and E_0 are calibrated to equal inhibitory effect: $a(0) = a(1)$.* Strict drug antagonism *is then said to hold in (27) when the function $a(\theta)$ is* **concave***: $a''(\theta) < 0$ for all $\theta \in (0, 1)$.*

8.2. **Generically, the optimal combination changes through time.** We now assume that the drugs are 'mathematically distinct', in the sense for example of having different modes of action or having different binding pockets on the same protein target. This allows us to quite reasonably impose inequalities on the structure of $\mathcal{F}$ that will hold in a generic sense. For instance, let us define the following, somewhat obscure, function whose meaning will become clear below,

$$b(\theta) = (\boldsymbol{w}, d_{\boldsymbol{x}}\mathcal{F}(\boldsymbol{x}_0, \theta D_0, (1-\theta)E_0)[\mathcal{F}(\boldsymbol{x}_0, \theta D_0, (1-\theta)E_0)]). \tag{29}$$

and assume the non-degeneracy condition that $b(\theta_{\text{syn}}) \neq 0$. The physical interpretation of this condition is not obvious, but it is the mathematical nature of this condition that allows us to prove that $\theta_{\text{opt}}(T)$ is not constant in T, even for very small T, as follows.

Theorem 1. *Assuming strict drug synergy is satisfied in (27) and that condition (29) holds, then there is a Taylor expansion*

$$\theta_{opt}(T) = \theta_{syn} - \overbrace{\left(\frac{b'(\theta_{syn})}{6a''(\theta_{syn})}\right)}^{\textit{divergence rate: } \boldsymbol{\rho}} T + O(T^2).$$

Therefore $\theta_{opt}(T)$ is approximately equal to optimal synergy for small T, but is not a constant function of T in general and the rate of divergence of these two quantities depends on the convexity of the interaction function, $a(\theta)$.

Proof. (sketch) As a result of (26), for each $T > 0$, the interval of existence of solutions of the differential equation (25) can be extended to the interval $[0, 1]$. Hence, (27) can be written as the zeros of a well-defined and smooth mapping, $\mathcal{G}(\boldsymbol{x}; \theta, T) = 0$, where $\mathcal{F} : C_0^1 \times [0, 1] \times [0, \infty) \to C^0$. Here, C_0^1 is the space of continuously differentiable functions, $\boldsymbol{x}(t)$, defined on the closed unit interval $[0, 1]$ taking values in $\mathbb{R}^n$ such that $\boldsymbol{x}(0) = \boldsymbol{x}_0$ where C^0 is the analogous space of continuous functions; both spaces are assumed to be supplied with appropriate C^1 and C^0 norms to make them Banach spaces. Explicitly, the nonlinear mapping $\mathcal{G}$ is given by

$$\mathcal{G}(\boldsymbol{x}; \theta, T) = -\frac{d}{dt}\boldsymbol{x} + T \cdot \mathcal{F}(\boldsymbol{x}, \theta D_0, (1-\theta)E_0).$$

We can trivially solve equation $\mathcal{G}(\boldsymbol{x}; \theta, T) = 0$ by setting $T = 0$ because $\mathcal{G}(\boldsymbol{x}_0; \theta, 0) \equiv 0$ where $\boldsymbol{x}_0 \in C_0^1$ is the constant function and $\theta \in [0, 1]$ is arbitrary. Now note that the partial $\boldsymbol{x}$-derivative of $\mathcal{G}$ satisfies $d_{\boldsymbol{x}}\mathcal{G}(\boldsymbol{x}_0; \theta, 0) = \frac{d}{dt}$. As the differential operator $\frac{d}{dt}$ is a bounded linear isomorphism from C_0^1 to C^0, we can apply the implicit function theorem (see [33] for a formulation appropriate to our needs) to locally solve $\mathcal{G}(\boldsymbol{x}; \theta, T) = 0$ for $\boldsymbol{x}$ as a function of (θ, T). Using (26) and the compactness of the interval $[0, 1]$, the resulting function $\boldsymbol{x}(\theta, T)$ can be extended smoothly using the implicit function theorem so that its domain encompasses all values of θ from 0 to 1, $[0, 1] \times [0, T_0)$ say for some $T_0 > 0$. Being a smooth function, the resulting function may then be expanded in powers of T as $\mathcal{F}$ was assumed to be infinitely differentiable. Hence,

$$\boldsymbol{x}(t; \theta, T) = X_0(t; \theta) + T \cdot X_1(t; \theta) + T^2 \cdot X_2(t; \theta) + O(T^3),$$

where the dependence of the expansion on the variable θ will be suppressed in the following for brevity.

From equation (25),

$$\frac{d}{dt}X_0 + T\frac{d}{dt}X_1 + T^2\frac{d}{dt}X_2 + O(T^3) = T \cdot \mathcal{F}\left(X_0 + TX_1 + T^2X_2 + O(T^3), \theta D_0, (1-\theta)E_0\right)$$

and we can also expand the latter in powers of T. Comparing powers of T in this expansion gives:

$O(T^0)$: $\frac{d}{dt}X_0 = 0, X_0(0) = \boldsymbol{x}_0$,
$O(T^1)$: $\frac{d}{dt}X_1 = \mathcal{F}(\boldsymbol{x}_0, \theta D_0, (1-\theta)E_0), X_1(0) = 0$ and
$O(T^2)$: $\frac{d}{dt}X_2 = \mathcal{F}_{\boldsymbol{x}}(\boldsymbol{x}_0, \theta D_0, (1-\theta)E_0)[X_1(t)], X_2(0) = 0$

whence

$$\boldsymbol{x}(t; \theta, T) = \boldsymbol{x}_0 + tT \cdot \mathcal{F}_0(\theta) + \frac{t^2T^2}{4}C_0(\theta)[\mathcal{F}_0(\theta)] + O(T^3)$$

where $\mathcal{F}_0(\theta) = \mathcal{F}(\boldsymbol{x}_0, \theta D_0, (1-\theta)E_0)$ and $C_0(\theta) = \mathcal{F}_{\boldsymbol{x}}(\boldsymbol{x}_0, \theta D_0, (1-\theta)E_0)$. As a result,

$$\begin{aligned}
J(\theta, T) &= \int_0^1 \left(\boldsymbol{w}, \boldsymbol{x}_0 + tT \cdot \mathcal{F}_0 + \frac{t^2T^2}{4} C_0[\mathcal{F}_0] + O(T^3) \right) dt \\
&= (\boldsymbol{w}, \boldsymbol{x}_0) + \frac{T}{2}(\boldsymbol{w}, \mathcal{F}_0(\theta)) + \frac{T^2}{12}(\boldsymbol{w}, C_0(\theta)[\mathcal{F}_0(\theta)]) + O(T^3) \\
&= (\boldsymbol{w}, \boldsymbol{x}_0) + \frac{T}{2} a(\theta) + \frac{T^2}{12} b(\theta) + O(T^3) \\
&= (\boldsymbol{w}, \boldsymbol{x}_0) + \frac{T}{2}\left(a(\theta) + \frac{T}{6} b(\theta) + O(T^2) \right). \qquad (30)
\end{aligned}$$

We define $j(\theta, T) = a(\theta) + \frac{T}{6} b(\theta) + O(T^2)$ so that $J(\theta, T) = (\boldsymbol{w}, \boldsymbol{x}_0) + \frac{T}{2} j(\theta, T)$.

From the strict drug synergy property, the minimum of $j(\theta, 0)$ occurs when $\theta = \theta_{\text{syn}} \in (0, 1)$. Moreover, at the minimum of j, there results $\frac{\partial j}{\partial \theta}(\theta, T) = 0$, provided T is small enough. Since $\frac{\partial^2 j}{\partial \theta^2}(\theta_{\text{syn}}, 0) = a''(\theta_{\text{syn}}) < 0$ which is a non-zero quantity by the assumption of strict drug synergy, we may solve $\frac{\partial j}{\partial \theta}(\theta, T) = 0$ for θ as a function of T by applying the implicit function theorem. At this solution $\theta = \theta_{\text{opt}}(T)$, say, and it follows by definition that $\theta_{\text{opt}}(0) = \theta_{\text{syn}}$. Hence, because $\frac{\partial j}{\partial \theta}(\theta_{\text{opt}}(T), T) \equiv 0$,

$$a'(\theta_{\text{opt}}(T)) + \frac{T}{6} b'(\theta_{\text{opt}}(T)) + O(T^2) \equiv 0$$

and, differentiating with respect to T, we establish

$$(\theta_{\text{opt}})'(T) \left(a''(\theta_{\text{opt}}(T)) + \frac{T}{6} b''(\theta_{\text{opt}}(T)) \right) + b'(\theta_{\text{opt}}(T))/6 + O(T) \equiv 0.$$

The result follows on setting $T = 0$ and expanding $\theta_{\text{opt}}(T)$ as a power series because $(\theta_{\text{opt}})'(0) a''(\theta_{\text{syn}}) + b'(\theta_{\text{syn}})/6 = 0$. □

Remark 3. *We end with the following observations.*

(1) *The expansion of the drug interaction profile, $J(\theta, T)$, in (30) might be described as having the structure*

$$\overbrace{\textit{initial population size}}^{(\boldsymbol{w}, \boldsymbol{x}_0)} + \overbrace{\textit{short-term synergy}}^{Ta(\theta)} + \overbrace{\textit{fastest adaptation}}^{T^2 b(\theta)} + \overbrace{\textit{slower adaptation}}^{O(T^3)}.$$

(2) *Do note that while this analysis is sufficient to show that optimal combinations based on short-term synergy are not likely to be stable to changes in population structure, the argument does not show that synergy is necessarily lost and replaced with an antagonistic drug interaction profile. Whether or not the smile-frown transition occurs is a system-specific property in the sense that it depends on the nature of $b(\theta)$, in particular whether or not it is a concave function, and how large T becomes over the course of the experiment.*

(3) *Note that the rate of divergence between synergy and the optimal therapy, ρ, in the statement of Theorem 1 depends explicitly on the drug synergy measure $a''(\theta)$.*

9. Final comment: single cell synergy and population synergy

It is stated in [34] that studies of drug interactions do not account for population heterogeneities:

> *'Much of drug interaction theory ... rests upon the assumption that the drug combination is acting upon a single, antibiotic-susceptible population of cells.'*

The present study indicates clear reasons why the study of growth inhibition of homogeneous populations of bacteria has the potential to provide misleading information in terms of how drugs interact for treatments of prolonged durations, meaning more than one day.

To be more precise, consider the growth inhibition function $G(S, D, E)$ in the model defined by equation (23). We have assumed that the interaction function

$$i(\theta) = G(S, \theta D_0, (1-\theta)E_0)$$

takes its minimum at a value of θ strictly between 0 and 1 for all possible dosages D_0 and E_0, thus representing synergy. Now, as a result, one could argue that any population of cells for which the growth rate of every subpopulation is described by a function of this synergistic form must also exhibit synergy when measuring

population densities, after all the growth rate reduction for each cell exhibits synergy. This seems almost obvious.

However, the results of this paper demonstrate that it is wrong to make this inference. The reason for this, at least in our modelling framework, is the nonlinearity of the system that results from accounting for the loss of essential metabolites, like carbon, from the environment as the cells grow. Only if carbon is not limiting, $S = \infty$ say, can this inference work. If it is limiting, our work shows this intuition can fail.

References

[1] Loewe S (1953) The problem of synergism and antagonism of combined drugs. Arzneimittelforschung 3: 285–290.

[2] Greco WR, Bravo G, Parsons JC (1995) The search for synergy: a critical review from a response surface perspective. Pharmacol Rev 47: 331–385.

[3] Cokol M, Chua HN, Tasan M, Mutlu B, Weinstein ZB, et al. (2011) Systematic exploration of synergistic drug pairs. Mol Syst Biol 7: 544.

[4] Ehrlich P (1913) Address in pathology on chemotherapeutics. The Lancet : 445-451.

[5] Hegreness M, Shoresh N, Damian D, Hartl D, Kishony R (2008) Accelerated evolution of resistance in multidrug environments. PNAS 105: 13977–13981.

[6] Bentley DR, Balasubramanian S, Swerdlow HP, Smith GP, Milton J, et al. (2008) Accurate whole human genome sequencing using reversible terminator chemistry. Nature 456: 53–59.

[7] Kelley DR, Schatz MC, Salzberg SL (2010) Quake: quality-aware detection and correction of sequencing errors. Genome Biol 11: R116.

[8] Cox MP, Peterson DA, Biggs PJ (2010) Solexaqa: At-a-glance quality assessment of illumina second-generation sequencing data. BMC Bioinformatics 11: 485.

[9] Ferenci T, Zhou Z, Betteridge T, Ren Y, Liu Y, et al. (2009) Genomic sequencing reveals regulatory mutations and recombinational events in the widely used MC4100 lineage of Escherichia coli K-12. J Bacteriol 191: 4025–4029.

[10] Li H, Durbin R (2009) Fast and accurate short read alignment with Burrows-Wheeler transform. Bioinformatics 24: 1754–1760.

[11] Wu TD, Nacu S (2010) Fast and snp-tolerant detection of complex variants and splicing in short reads. Bioinformatics 26: 873–881.

[12] Li H, Handsaker B, Wysoker A, Fennell T, Ruan J, et al. (2009) The sequence alignment/map format and samtools. Bioinformatics 25: 2078–2079.

[13] Quinlan A, Hall I (2010) Bedtools: A flexible framework for comparing genomic features. Bioinformatics 26.

[14] Wei Z, Wang W, Hu P, Lyon G, Hakonarson H (2011) Snver: a statistical tools for variant calling in analysis of pooled or individual next-generation sequencing data. Nucleic Acids research : 1–13.

[15] Koboldt D, Zhang Q, Larson D, Shen D, McLellan M, et al. (2012) Varscan 2: Somatic mutation and copy number alteration discovery in cancer by exome sequencing. Genome Research 22: 568–576.

[16] Chen K, Wallis J, McLellan M, Larson D, Kalicki J, et al. (2009) Breakdancer: an algorithm for high-resolution mapping of genomic structural variation. nature Methods 6: 677–684.

[17] Abyzov A, Urban AE, Snyder M, Gerstein M (2011) Cnvnator: An approach to discover, genotype, and characterize typical and atypical cnvs from family and population genomic sequencing. Genome Research 21: 974–984.

[18] Ye K, Schulz M, Long Q, Apweiler R, Ning Z (2009) Pindel: a pattern growth approach to detect break points of large deletions and medium sized insertions from paired-end short reads. Bioinformatics 25: 2865-2871.

[19] Cohen S, Haechler H, Levy S (1993) Resistance (mar) locus in Escherichia coli. Journal of bacteriology 175: 1484-1492.

[20] Ma D, MAlberti, Lynch C, Nikaido H, Hearst JE (1996) The local repressor AcrR plays a modulating role in the regulation of acrAB genes of Escherichia coli by global stress signals. Molecular microbiology 19: 101–112.

[21] Randall L, Woodward M (2002) The multiple antibiotic resistance (mar) locus and its significance. Research in Veterinary Science 72: 87–93.

[22] George AM, Levy SB (1983) Amplifiable resistance to tetracycline, chloramphenicol, and other antibiotics in Escherichia coli: involvement of a non-plasmid-determined efflux of tetracycline. J Bacteriol 155: 531–540.

[23] Martin R, Rosner J (2004) Transcriptional and translational regulation of the marRAB multiple antibiotic resistance operon in Escherichia coli. Molecular Microbiology 53: 183–191.

[24] Barbosa T, Levy S (2000) Differential expression of over 60 chromosomal genes in Escherichia coli by constitutive expression of MarA. Journal of bacteriology 182: 3467–3474.

[25] Pomposiello P, Bennik M, Demple B (2001) Genome-wide transcriptional profiling of the Escherichia coli responses to superoxide stress and sodium salicylate. Journal of Bacteriology 183: 3890–3902.

[26] Alekshun M, Levy S (1997) Regulation of chromosomally mediated multiple antibiotic resistance: the mar regulon. Antimicrobial agents and chemotherapy 41: 2067–2075.

[27] Ma D, Cook D, MAlberti, NGPon, Nikaido H, et al. (1995) Genes acrA and acrB encode a stress-induced efflux system of Escherichia coli. Molecular microbiology 16: 45–55.

[28] Okusu H, Ma D, Nikaido H (1996) AcrAB efflux pump plays a major role in the antibiotic resistance phenotype of Escherichia coli multiple-antibiotic-resistance (Mar) mutants. J Bacteriol 178: 306–308.

[29] McMurry L, Oethinger M, Levy S (1998) Overexpression of marA, sod, or acrAB produces resistance to triclosan in laboratory and clinical strains of Escherichia coli. FEMS Microbiology Letters 166: 305–309.

[30] Mazzariol A, Tokue Y, Kanegawa T, Cornaglia G, Nakaido H (2000) High-level fluoroquinolone-resistant clinical isolates of Escherichia coli overproduce multidrug efflux protein AcrA. Antimicrobial agents and chemotherapy 44: 3441–3443.

[31] Nicoloff H, Perreten V, McMurry LM, Levy SB (2006) Role for tandem duplication and lon protease in AcrAB-TolC-dependent multiple antibiotic resistance (Mar) in an Escherichia coli mutant without mutations in marRAB or acrRAB. J Bacteriol 188: 4413–4423.

[32] Nicoloff H, Perreten V, Levy S (2007) Increased genome instability in Escherichia coli lon mutants: Relation to emergence of multiple-antibiotic-resistant (Mar) mutants caused by insertion sequence elements and large tandem genomic amplifications. Antimicrobial Agents and Chemotherapy 51: 1293–1303.

[33] Ambrosetti A, Prodi G (1993) A Primer of Nonlinear Analysis. Cambridge University Press.

[34] Drusano GL, Liu W, Fregeau C, Kulawy R, Louie A (2009) Differing effects of combination chemotherapy with meropenem and tobramycin on cell kill and suppression of resistance of wild-type Pseudomonas aeruginosa PAO1 and its isogenic MexAB efflux pump-overexpressed mutant. Antimicrob Agents Chemother 53: 2266–2273.

Appendix A. Parameter values

Parameter values for core inhibition model of section 6.1 are given in Table S4. Parameter values for the drug efflux model of section 6.4 are given in Table S4. Simulations were conducted in Matlab using `ode15s` or in Python using a BDF method implemented in `odeint` from the `Scipy.integrate` package.

Parameter	*Description*	*Value*
$S(0)$	Glucose supply	$2000\,\mu g/ml$
$D(0)$	Basal concentration of drug D	$0.15\,\mu g/ml$
$E(0)$	Basal concentration of drug E	$9\,\mu g/ml$
$V_{\max}$	Maximal uptake rate	$2.6 \times 10^{-6}\mu g/\text{cell}/h$
K	Bacterial half-saturation constant	$0.0527\,\mu g/ml$
c	Resource conversion	1.851×10^{4} cell/μg
d	Drug D binding rate	$1.469 \times 10^{-9}\ \mu g$/cell
e	Drug E binding rate	$1.44 \times 10^{-9}\ \mu g$/cell
η	Dilution parameter	$\sim 1\%$ of volume

Table S3 − Parameter values used for the simulations of the model presented in section 6.1.

Parameter	*Description*	*Value*
S_0	Glucose supply	$2000\,\mu g/ml$
D_0	Basal concentration of drug D	$0.15\,\mu g/ml$
E_0	Basal concentration of drug E	$9\,\mu g/ml$
$V_{\max}$	Maximal uptake rate	$1139.6\,\mu g/\mathrm{OD}_{600nm}/h$
K	Bacterial half-saturation constant	$0.53882\,\mu g/ml$
c	Resource conversion	$0.000315\,\mathrm{OD}_{600nm}/\mu g$
$\kappa_e, \kappa_d, \kappa_m$	Drug inhibition parameters	$0.2ml/\mu g, 300ml/\mu g, 4000[ml/\mu g]^2$
v_e, k_e	Efflux parameters for drug E	$3987.3ml/\mathrm{OD}_{600nm}/h, 19.681$ (dimensionless)
v_d, k_d	Efflux parameters for drug D	$3999.1ml/\mathrm{OD}_{600nm}/h, 0.8$ (dimensionless)
φ_e, φ_d	Diffusion equilibration	$93.068ml/\mathrm{OD}_{600nm}/h, 0.041346ml/\mathrm{OD}_{600nm}/h$
δ, Δ	Gene copy rate, pump decay coefficient	0.0025 per gene/h, 18 (dimensionless)
g	pump gene transcription coefficient	0.5 (dimensionless)
d_D, d_E	drug decay parameters	in the interval $[\frac{1}{24}\ln(0.8), 0]$ per day
10^7 cells/ml (CFUs/ml)	cell density measure	0.13 ($\pm$0.02) $\mathrm{OD}_{600\mathrm{nm}}$

Table S4 − Model parameters used in the numerical simulations discussed in section 6.4

Appendix B. Genes annotated in the duplicated region

start_position	end_position	gene	annotation
**Antibiotic binding and resistance genes**			
297113	298270	ampH	beta-lactam binding protein AmpH
370854	372626	mdlA	putative multidrug transporter membrane/ATP-binding components
3383237	386386	acrB	multidrug efflux system protein
360871	363225	lon	DNA-binding ATP-dependent protease La
386409	387602	acrA	multidrug efflux system
387744	388391	acrR	regulates the acrAB operon which is involved in susceptibility to dephalothin and cephaloridineDNA-binding transcriptional repressor AcrR
72619	374400	mdlB	putative multidrug transporter membrane/ATP-binding components
405459	406679	fsr	putative fosmidomycin efflux system
470298	470630	emrE	member of the small MDR (multidrug resistance) family of transporters; in Escherichia coli this protein provides resistance against a number of positively charged compounds including ethidium bromide and erythromycin; proton-dependent secondary transporter which exchanges protons for compound translocationmultidrug efflux protein
564735	565946	dacA	penicillin-binding protein 5; removes C-terminal D-alanyl residues from sugar-peptide cell wall precursorsD-alanyl-D-alanine carboxypeptidase
567184	568296	mrdB	cell wall shape-determining protein
568299	570200	mrdA	penicillin-binding protein 2
**Transporter genes**			
22969	324342	proY	cryptic permease that may be involved in the transport of proline across the inner membrane
277442	278653	mhpT	putative 3-hydroxyphenylpropionic transporter
287215	288177	tauA	with TauB and TauC responsible for taurine uptake
288190	288957	tauB	Part of the ABC transporter complex tauABC
288954	289781	tauC	taurine transporter subunit
289778	290629	tauD	taurine metabolism
298622	299842	sbmA	involved in uptake of microcin J25
313280	314464	araJ	member of the major facilitator superfamily (MFS) of transporters; unknown function; may be associated with transport or processing of arabinose polymersMFS transport protein AraJ
329630	331477	secD	part of the preprotein secretory system; when complexed with proteins SecF and YajC, SecDFyajC stimulates the proton motive force-driven protein translocation, and appears to be required for the release of mature proteins from the extracytoplasmic side of the membranepreprotein translocase subunit SecD
331488	332459	secF	forms a complex with SecD and YajC; SecDFyajC stimulates the proton motive force-driven protein translocation
3354053	355528	ampG	peptidoglycan recycling; member of major facilitator superfamily MFS; inner membrane protein-muropeptide transporter
47285	348649	yajR	putative transporter
367595	369295	ybaE	putative transporter subunit: periplasmic-binding component of ABC superfamily
374949	376235	amtB	ammonium transporter
378431	378544	ffs	4.5S sRNA component of Signal Recognition Particle (SRP); co-translational protein translocation into and possibly through membranes
403545	405221	ybaL	member of the CPA-2 family of antiporters
410858	413362	copA	copper exporting ATPase
414559	415851	ybaT	putative transporter
417902	418579	ybbL	putative ABC transporter ATP-binding protein YbbL
421716	422402	ybbA	putative ABC transporter ATP-binding protein YbbA
495311	496753	cusS	sensor kinase CusS
496743	497426	cusR	response regulator in two-component regulatory system with CusS
497583	498956	cusC	with CusA, CusB and CusF is part of a cation efflux system that mediates resistance to copper and silvercopper/silver efflux system outer membrane protein CusC
499114	499446	cusF	copper-binding protein
499462	500685	cusB	with CusA, CusC and CusF is part of a cation efflux system that mediates resistance to copper and silvercopper/silver efflux system membrane fusion protein CusB
500697	503840	cusA	copper/silver efflux system, membrane component
503942	505318	pheP	phenylalanine transporter
586513	587238	gltL	glutamate and aspartate transporter subunit
587238	587912	gltK	glutamate and aspartate transporter subunit
587912	588652	gltJ	glutamate and aspartate transporter subunit
588822	589730	gltI	glutamate and aspartate transporter subunit
556566	557951	dcuC	responsible for the transport of C4-dicarboxylates during anaerobic growthC4-dicarboxylate transporter DcuC
**Transposons and integrases**			
273324	274340	insH	IS5 transposase and trans-activator
293722	294588	insF	IS3 element protein InsF
294585	294893	insE	IS3 element protein InsE
283334	283699	insC	IS2 OrfAB forms an overlapping reading frame with orfB to form fusion protein OrfAB due to ribosomal frameshifting
283657	284562	insD	IS2 OrfB
425244	429524	rhsD	rhsD element protein
466798	467961	intD	DLP12 prophage; putative integrase
468816	469124	insE	IS3 element protein InsE
469121	469987	insF	IS3 element protein InsF
470885	472411	ybcK	DLP12 prophage; putative recombinase
472876	473427	ybcL	DLP12 prophage; similar to PEBP/RK1P protein family in eukaryotes that inhibits MEK phosphorylation by Raf-1; crystal structure suggests similar properties but exact function is unknown-putative kinase inhibitor
473437	474234	ybcM	DLP12 prophage; putative DNA-binding transcriptional regulator

474449	474904	ybcN	DLP12 prophagehypothetical protein
474904	475074	ninE	DLP12 prophage; conserved protein similar to phage 82 and lambda proteinsprophage protein NinE
475067	475357	ybcO	DLP12 prophage protein
475354	475716	rusA	DLP12 prophage; Holliday junction resolvaseendodeoxyribonuclease RUS
475713	475853	ylcG	DLP12 prophage protein
475939	476322	quuD	DLP12 prophage; putative antitermination protein
476720	477736	insH	IS5 transposase and trans-activator
479381	479596	essD	DLP12 prophage; putative phage lysis protein
479596	480093	arrD	DLP12 prophage; putative lysozyme
480090	480551	rzpD	DLP12 prophage; putative murein endopeptidase
480310	480492	rzoD	DLP12 prophage; putative lipoprotein
480583	480876	borD	DLP12 prophage; putative lipoprotein
481167	481577	ybcV	DLP12 prophage protein
481863	482069	ybcW	DLP12 prophage protein
482234	482428	ylcI	hypothetical protein
482817	483362	nohB	DLP12 prophage; DNA packaging protein
485664	486413	appY	DLP12 prophage; DNA-binding transcriptional activator
510048	511160	insL	IS186/IS421 transposase
589980	590996	insH	IS5 transposase and trans-activator
**Metabolism related genes**			
274524	274907	mhpD	2-keto-4-pentenoate hydratase
274904	275854	mhpF	catalyzes formation of acetyl-CoA from acetalaldehydeacetaldehyde dehydrogenase
275851	276864	mhpE	catalyzes formation of pyruvate and acetaldehyde from 4-hydroxy-2-ketovaleric acid, degradation of phenylpropionate4-hydroxy-2-ketovalerate aldolase
278755	279294	yaiL	nucleoprotein-polynucleotide-associated enzyme
279518	280351	frmB	putative esterase
280445	281554	frmA	alcohol dehydrogenase
284722	285918	yaiP	putative glucosyltransferase
290736	291710	hemB	catalyzes formation of porphobilinogen from 5-aminolevulinatedelta-aminolevulinic acid dehydratase
301812	302906	ddl	D-alanine–D-alanine ligase
3305686	306801	adrA	catalyzes the conversion of 2 GTP into c-di-GMP
306818	307627	proC	catalyzes the formation of L-proline from pyrroline-5-carboxylatepyrroline-5-carboxylate reductase
303730	305145	phoA	alkaline phosphatase
312127	313035	mak	catalyzes phosphorylation of fructose; cytosolic enzymefructokinase
3324498	326315	malZ	maltodextrin glucosidase
326320	326901	acpH	Converts holo-ACP to apo-ACP by hydrolytic cleavage of the phosphopantetheine prosthetic group from ACPacyl carrier protein phosphodiesterase
326994	328064	queA	Synthesizes oQ from preQ1 in a single S-adenosylmethionine-requiring stepS-adenosylmethionine–tRNA ribosyltransferase-isomerase
328120	329247	tgt	Exchanges the guanine residue with 7-aminomethyl-7-deazaguanine in tRNAs with GU(N) anticodons (tRNA-Asp, -Asn, -His and -Tyr)queuine tRNA-ribosyltransferase
329270	329602	yajC	member of preprotein translocase; forms a heterotrimer with SecD and SecF; links the SecD/SecF/YajC/YidC complex with the SecY/SecE/SecG complexpreprotein translocase subunit YajC
3335438	336541	ribD	riboflavin biosynthesis protein
336630	337100	ribH	RibE; 6,7-diimethyl-8-ribityllumazine synthase; DMRL synthase; lumazine synthase; beta subunit of riboflavin synthase;
337120	337539	nusB	Regulates rRNA biosynthesis by transcriptional antiterminationtranscription antitermination protein NusB
337617	338594	thiL	catalyzes the formation of thiamine diphosphate from thiamine phosphate ant ATPthiamine monophosphate kinase
338572	339090	pgpA	hydrolyzes phosphatidylglycerophosphate to produce phosphatidylglycerol and phosphatephosphatidylglycerophosphatase A
339144	340118	yajO	2-carboxybenzaldehyde reductase
340298	342160	dxs	catalyzes the formation of 1-deoxy-D-xylulose 5-phosphate from pyruvate and D-glyceraldehyde 3-phosphate1-deoxy-D-xylulose-5-phosphate synthase
342185	343084	ispA	geranyltranstransferase
343084	343326	xseB	catalyzes the bidirectional exonucleolytic cleavage of DNAexodeoxyribonuclease VII small subunit
343532	344980	thiI	Required for the synthesis of the thiazole moietythiamine biosynthesis protein ThiI
345034	345624	yajL	DJ-1 family protein
345587	346498	panE	ketopantoate reductase
0357116	358414	tig	Tig; RopA; peptidyl-prolyl cis/trans isomerase
358660	359283	clpP	hydrolyzes proteins to small peptides; with the ATPase subunits ClpA or ClpX, ClpP degrades specific substratesATP-dependent Clp protease proteolytic subunit
359409	360683	clpX	binds and unfolds substrates as part of the ClpXP proteaseATP-dependent protease ATP-binding subunit ClpX
363434	363706	hupB	histone-like DNA-binding proteintranscriptional regulator HU subunit beta
363898	365769	ppiD	peptidyl-prolyl cis-trans isomerase
8388	308912	aroL	type II enzyme similar to type I but differentially regulated and with a lower Km; catalyzes the formation of shikimate 3-phosphate from shikimate in aromatic amino acid biosynthesisshikimate kinase
366835	367530	queC	quenosine biosynthesis
369395	370213	cof	thiamin pyrimidine pyrophosphate hydrolase
376284	377144	tesB	acyl-CoA thioesterase II
3393395	393946	apt	catalyzes a salvage reaction resulting in the formation of AMP
377965	378354	ybaZ	putative methyltransferase
374581	374919	glnK	indirectly regulates nitrogen metabolism
381350	381901	maa	maltose O-acetyltransferase
394075	396006	dnaX	catalyzes the DNA-template-directed extension of the 3'-end of a DNA strand;

399158	399802	adk	essential enzyme that recycles AMP in active cells
400038	401000	hemH	protoheme ferro-lyase
400997	401956	aes	acetyl esterase
402108	403412	gsk	inosine/guanosine kinase
406897	408549	ushA	catalyzes the degradation of periplasmic UDP-glucose to uridine, glucose-1-phosphate and inorganic phosphate
4420323	421132	ybbO	short chain dehydrogenase
421122	421748	tesA	multifunctional acyl-CoA thioesterase I/protease I/lysophospholipase L1
432115	433209	ybbB	catalyzes the selenophosphate-dependent transfer of selenium from selenophosphate for conversion of 2-thiouridine to 2-selenouridine at the wobble position in tRNAtRNA 2-selenouridine synthase
434434	434916	allA	catalyzes the formation of glyoxylate from (S)-ureidoglycolateureidoglycolate hydrolase
413624	414556	ybaS	catalyzes the formation of glutamate from glutamineglutaminase
435899	437680	gcl	catalyzes the formation of 2-hydroxy-3-oxopropanoate (tartronate semialdehyde) from two molecules of glyoxylateglyoxylate carboligase
437693	438469	hyi	hydroxypyruvate isomerase
438569	439447	glxR	tartronate semialdehyde reductase, NADH-dependent
439616	441070	ybbW	allantoin permease
441130	442491	allB	Plays a crucial role on both purine and pyrimidine metabolismallantoinase
442548	443849	ybbY	putative purine permease YbbY
4446040	447275	allC	allantoate amidohydrolase and N-carbamoyl-L-amino acid amidohydrolase are very similar; the allantoate amidohydrolase from Escherichia coli forms a dimer and binds zinc ions for catalytic activity and catalyzes the conversion of allantoate to (S)-ureidoglycolate and ammonia; carbamoyl amidohydrolase from Bacillus sp. converts N-carbamoyl amino acids to amino acids, ammonia, and carbon dioxideallantoate amidohydrolase
447297	448346	allD	ureidoglycolate dehydrogenase
43871	445016	glxK	glycerate kinase
452422	453315	ybcF	carbamate kinase
453510	454577	purK	With PurE catalyzes the conversion of aminoimidazole ribonucleotide to carboxyaminoimidazole ribonucleotide in the de novo purine nucleotide biosynthetic pathwayphosphoribosylaminoimidazole carboxylase ATPase subunit
454574	455083	purE	N5-carboxyaminoimidazole ribonucleotide mutase
455201	455923	lpxH	catalyzes the formation of 2,3=diacylglucosamine 1-phosphate from UDP-2,3=diacylglucosamineUDP-2,3-diacylglucosamine hydrolase
455926	456420	ppiB	peptidyl-prolyl cis-trans isomerase B
456594	457979	cysS	catalyzes a two-step reaction; charges a cysteine by linking its carboxyl group to the alpha-phosphate of ATP then transfers the aminoacyl-adenylate to its tRNAcysteinyl-tRNA synthetase
458858	459724	folD	catalyzes the formation of 5,10-methenyltetrahydrofolate from 5,10-methylenetetrahydrofolate and subsequent formation of 10-formyltetrahydrofolate from 5,10-methenyltetrahydrofolatebifunctional 5,10-methylene-tetrahydrofolate dehydrogenase/ 5,10-methylene-tetrahydrofolate cyclohydrolase
506754	507407	nfsB	catalyzes the reduction of nitroaromatic compounds such as nitrofurazone, quinones and the anti-tumor agent CB1954; NAD(P)H-dependent; oxygen insensitivedihydropteridine reductase
508248	509366	ybdK	ATP-dependent; carboxylate-amine ligase with weak glutamate–cysteine ligase activitycarboxylate-amine ligase
511442	512071	entD	phosphopantetheinyltransferase component of enterobactin synthase multienzyme complex
512237	514477	fepA	outer membrane receptor of ferric enterobactin and colicins B and D
514798	515922	fes	enterobactin/ferric enterobactin esterase
515925	516143	ybdZ	hypothetical protein
516140	520021	entF	with EntB, EntD, and EntE forms the multienzyme complex enterobactin synthase
520237	521370	fepE	part of the ferric enterobactin transport system
521367	522182	fepC	with FepBDE is involved in the transport of ferric enterobactiniron-enterobactin transporter ATP-binding protein
522179	523171	fepG	with FepBCD is involved in the transport of ferric enterobactiniron-enterobactin transporter permease
523168	524172	fepD	with FepBCG is involved in the transport of ferric enterobactiniron-enterobactin transporter membrane protein
524283	525533	entS	protein p43; inner membrane protein that exports enterobactin to the periplasmic space; member of the major facilitator superfamily (MFS) of transportersenterobactin exporter EntS
525537	526493	fepB	with FepCDG is involved in the transport of ferric enterobactiniron-enterobactin transporter periplasmic binding protein
526868	528043	entC	isochorismate synthase 1
528053	529663	entE	bifunctional 2,3-dihydroxybenzoate-AMP ligase/S-dihydroxybenzoyltransferase
529677	530534	entB	isochorismatase
530534	531280	entA	catalyzes the formation of 2,3-dihydroxybenzoate from 2,3-dihydro-2,3-dihydroxybenzoate; involved in the biosynthesis of siderophores, enterobactin, bacillibactin or vibriobactin2,3-dihydroxybenzoate-2,3-dehydrogenase
531877	533982	cstA	carbon starvation protein
534372	535460	ybdH	member of the iron-containing alcohol dehydrogenase family; unknown functionhypothetical protein
535569	536729	ybdL	catalyzes the transfer of an amino moietyputative aminotransferase
539810	540556	dsbG	Involved in disulfide bond formationdisulfide isomerase/thiol-disulfide oxidase
540928	541491	ahpC	with AhpF catalyzes the conversion of alkyl hydroperoxides to their corresponding alcohols; AhpC reduced the hydroperoxide substratealkyl hydroperoxide reductase subunit C
541736	543301	ahpF	alkyl hydroperoxide reductase, F52a subunit, FAD/NAD(P)-binding
544071	545309	ybdR	putative oxidoreductase, Zn-dependent and NAD(P)-binding
545540	545950	rnk	Regulates the synthesis of nucleoside triphosphates for nucleic acid synthesis, CTP for lipid synthesis, and GTP for protein elongationnucleoside diphosphate kinase regulator
546180	546986	rna	ribonuclease I
547100	548563	citT	citrate:succinate antiporter
548614	549492	citG	catalyzes the formation of 2'-(5"-triphosphoribosyl)-3'-dephospho-CoA from ATP and 3-dephospho-CoAtriphosphoribosyl-dephospho-CoA synthase

549467	550018	citX	2'-(5"-phosphoribosyl)-3'-dephospho-CoA transferase; holo-citrate lyase synthase; CitG forms the prosthetic group precursor 2'-(5"-triphosphoribosyl)-3'-dephospho-CoA which is then transferred to apo-ACP by CitX to produce holo-ACP and pyrophosphate2-(5"-triphosphoribosyl)-3'-dephosphocoenzyme-A synthase
550022	551554	citF	citrate lyase, citrate-ACP transferase (alpha) subunit
551565	552473	citE	citrate lyase, citryl-ACP lyase (beta) subunit
552470	552766	citD	acyl carrier protein; with CitE and CitF catalyzes the formation of oxaloacetate from citratecitrate lyase subunit gamma
552781	553839	citC	citrate lyase synthetase
558540	559100	pagP	catalyzes the transfer of palmitate to lipid Apalmitoyl transferase
561234	562199	lipA	catalyzes the radical-mediated insertion of two sulfur atoms into an acyl carrier protein (ACP) bound to an octanoyl group to produce a lipoyl grouplipoyl synthase
562408	563361	ybeF	putative DNA-binding transcriptional regulator
563620	564261	lipB	lipoyl/octanoyltransferase
585460	586395	rihA	Hydrolyzes with equal efficiency cytidine or uridine to ribose and cytosine or uracil, respectively; pyrimidine-specificribonucleoside hydrolase 1
**Other genes**			
281589	281864	frmR	formaldehyde-induced negative regulator of the frmRAB operonregulator protein FrmR
282052	282825	yaiO	hypothetical protein
286042	286599	yaiS	hypothetical protein
296444	297112	yaiV	putative DNA-binding transcriptional regulator
299855	300949	yaiW	putative DNA-binding transcriptional regulator
301008	301316	yaiY	putative inner membrane protein
301576	301788	yaiZ	putative inner membrane protein
303369	303629	iraP	hypothetical protein
305264	305584	psiF	hypothetical protein
3308962	309153	yaiA	hypothetical protein
309411	310088	aroM	hypothetical protein
310160	310444	yaiE	hypothetical protein
307747	308205	yaiI	hypothetical protein
31091	312002	rdgC	Required for efficient pilin antigenic variationrecombination associated protein
317733	318935	sbcD	with SbcC cleaves DNA hairpin structure, also has 5' single-strand endonuclease activityexonuclease subunit SbcD
319125	319814	phoB	two component response regulator for the phosphate regulon; PhoR phosphorylates PhoBtranscriptional regulator PhoB
319872	321167	phoR	membrane-associated histidine protein kinase, part of the two-component phosphate regulatory system phoR/phoBphosphate regulon sensor protein
321574	322893	brnQ	putative branched chain amino acid transporter (LIV-II)
31332588	332935	yajD	hypothetical protein
3314590	317736	sbcC	with SbcD cleaves DNA hairpin structures; also has 5' single-strand endonuclease activityexonuclease subunit SbcC
333112	333996	tsx	nucleoside channel phage T6/colicin K receptor
334295	334834	yajI	hypothetical protein
334985	335434	nrdR	transcriptional regulator NrdR
3365920	366291	ybaV	hypothetical protein
3349700	350029	cyoD	cytochrome o ubiquinol oxidase subunit IV
3378655	379008	ybaA	hypothetical protein
346666	347157	yajQ	putative nucleotide binding property based on structural studies of Haemophilus influenzae crystallized protein in PDB Accession Number 1IN0 and NMR studies of Escherichia coli YajQ; the YajQ protein from Pseudomonas synringae appears to play a role in activation of bateriophage phi6 segment L transcriptionputative nucleotide-binding protein
348798	349688	cyoE	converts protoheme IX and farnesyl diphosphate to heme Oprotoheme IX farnesyltransferase
350029	350643	cyoC	cytochrome o ubiquinol oxidase subunit III
350633	352624	cyoB	cytochrome o ubiquinol oxidase subunit I
352646	353593	cyoA	cytochrome o ubiquinol oxidase subunit II
366385	366783	ybaW	hypothetical protein
355572	356150	yajG	hypothetical protein
356455	356772	bolA	positive transcriptional regulator of morphogenetic pathway; controlling several genes involved in oxidative stress, acid stress, heat shock, osmotic shock, and carbon-starvation stresstranscriptional regulator BolA
370366	370824	ybaO	putative DNA-binding transcriptional regulator
377362	377934	ybaY	putative outer membrane lipoprotein
379050	380600	ylaB	hypothetical protein
380764	381234	ylaC	putative inner membrane protein
382073	382291	hha	with Hns involved in transcriptional regulation of hemolysin
382317	382691	ybaJ	hypothetical protein
388519	391881	kefA	small mechanosensitive ion channel (MscS) that opens in response to stretch forces in the membrane lipid bilayer; maintains cell turgor through accumulation and release of potassium; large protein class of MscSpotassium efflux protein KefA
392093	392254	ybaM	hypothetical protein
392268	392795	priC	PriC; protein involved in DNA replication; part of the primosome, a protein complex required to restart stalled replication forks;
392865	393242	ybaN	hypothetical protein
396059	396388	ybaB	hypothetical protein
396388	396993	recR	involved in a recombinational process of DNA repair, independent of the recBC complexrecombination protein RecR
397103	398977	htpG	molecular chaperoneheat shock protein 90
408586	409065	ybaK	hypothetical protein
409187	409268	sroB	Novel sRNA, function unknown
409269	410063	ybaP	hypothetical protein
410201	410542	ybaQ	putative DNA-binding transcriptional regulator

415976	416383	cueR	activator of copper-responsive regulon genes, DNA-binding transcriptional regulator CueR
416384	416842	ybbJ	hypothetical protein
4418566	419345	ybbM	putative inner membrane protein
419408	420262	ybbN	putative thioredoxin domain-containing protein
1422399	424813	ybbP	putative inner membrane protein
429564	429932	ybbC	hypothetical protein
416839	417756	qmcA	putative protease, membrane anchored
433278	434204	allS	activator of the allDC-ylbA operon involved in allantoin utilizationDNA-binding transcriptional activator AllS
434994	435809	allR	regulates operons involved in the utilization of allantoinDNA-binding transcriptional repressor AllR
445244	446029	ylbA	hypothetical protein
448663	450330	fdrA	multicopy suppressor of dominant negative ftsH mutationsmembrane protein FdrA
451610	452425	ylbF	hypothetical protein
458015	458536	ybcI	hypothetical protein
458644	458856	ybcJ	hypothetical protein
460195	460737	sfmA	putative fimbrial-like adhesin protein
460957	461649	sfmC	pilin chaperone, periplasmic
461680	464283	sfmD	putative outer membrane export usher protein
464319	465302	sfmH	putative fimbrial-like adhesin protein
465313	465828	sfmF	putative fimbrial protein
465831	466463	fimZ	activates the production of the major fimbrae protein FimAtranscriptional regulator FimZ
466706	466782	argU	tRNA-Arg
474351	474452	ylcH	hypothetical protein
486663	487616	ompT	outer membrane protease; involved in virulence in many organisms; OmpT; IcsP; SopA; Pla; PgtE; omptin; in Escherichia coli OmpT can degrade antimicrobial peptides;
488040	488084	pauD	Xaa-tRNA
488130	488891	envY	DNA-binding transcriptional activator of porin biosynthesis
489074	489964	ybcH	hypothetical protein
489965	492937	nfrA	bacteriophage N4 receptor, outer membrane subunit
492924	495161	nfrB	bacteriophage N4 adsorption protein B
505399	506646	ybdG	putative mechanosensitive channel
507501	507869	ybdF	hypothetical protein
507934	508182	ybdJ	putative inner membrane protein
509819	509971	hokE	toxic polypeptide, small
—- 531283	531696	ybdB	hypothetical protein
534165	534362	ybdD	hypothetical protein
536730	537359	ybdM	hypothetical protein
537332	538552	ybdN	hypothetical protein
538699	539601	ybdO	putative DNA-binding transcriptional regulator
543422	543850	uspG	universal stress protein UP12
554218	555876	dpiB	sensory histidine kinase in two-component regulatory system with citB
555845	556525	dpiA	regulates the expression of citAB in citrate fermentationtwo-component response regulator DpiA
559275	559484	cspE	member of the CspA family;
559538	559921	crcB	may be involved in chromosome condensation
560930	561133	tatE	TatE; similar to TatA and found in some proteobacteria; part of system that translocates proteins with a conserved twin arginine motif across the inner membrane
564362	564625	ybeD	hypothetical protein
566085	567173	rlpA	rare lipoprotein A
570231	570698	ybeA	SPOUT methyltransferase family protein; crystal structure shows homodimer; in Escherichia coli this protein methylates pseudouridine at position 1915 of the 23S ribosomal RNArRNA large subunit methyltransferase
570702	571019	ybeB	hypothetical protein
571279	571890	cobC	putative alpha-ribazole-5'-P phosphatase
571914	572555	nadD	transfers an adenyl group from ATP to NaMN to form nicotinic acid adenine dinucleotide (NaAD)
572557	573588	holA	required for the assembly and function of the DNAX complex which is required for the assembly of the beta subunit onto primed DNADNA polymerase III subunit delta
573588	574169	rlpB	rare lipoprotein B; involved in the assembly of LPS in the outer membraneLPS-assembly lipoprotein RlpB
574184	576766	leuS	leucine–tRNA ligase
577001	577483	ybeL	hypothetical protein
577553	578530	ybeQ	hypothetical protein
578694	579401	ybeR	hypothetical protein
579398	580825	djlB	putative chaperone
580835	581389	ybeT	hypothetical protein
581491	582198	ybeU	putative tRNA ligase
582195	583646	djlC	Hsc56 co-chaperone of HscC
583706	585376	hscC	Hsp70 family chaperone Hsc62, binds to RpoD and inhibits transcription
